# Supported Palladium Nanoparticles Catalyzed Intermolecular Carbopalladation of Nitriles and Organoboron Compounds

**DOI:** 10.3389/fchem.2022.855850

**Published:** 2022-05-09

**Authors:** Xin Liu, Dan Liu, Tegshi Muschin, Agula Bao, Chaolumen Bai, Yong-Sheng Bao

**Affiliations:** Inner Mongolia Key Laboratory of Green Catalysis, College of Chemistry and Environmental Science, Inner Mongolia Normal University, Hohhot, China

**Keywords:** supported palladium nanoparticles, aromatic ketones, nitriles, carbopalladation, green chemistry

## Abstract

The first heterogeneous catalyzed example for the direct synthesis of aromatic ketones via intermolecular carbopalladation of aliphatic nitriles and organoboron compounds was developed. This mild method proceeds with a supported palladium nanoparticles catalyst that could be reused and recycled five times. The fresh and used catalysts were characterized by XPS and TEM. The XPS analysis indicated that Pd^0^ was the active species for the reaction. This methodology provides a mild and cost-effective strategy for the efficient synthesis of ketones.

## Introduction

Palladium (Pd) catalysis has emerged as an efficient platform to construct various C–C bonds and C–X (X = N and O) bonds over the past decade (Chen et al., 2009; McGlacken and Bateman, 2009; Ruiz-Castillo and Buchwald, 2016; Biffis et al., 2018), while there are strict restrictions for the level of viable trace Pd impurities in food and pharmaceutical owing to its toxicity (Gandeepan et al., 2019). As a result, typical tedious workup procedures are necessary to isolate the product from the reaction mixture, which undoubtedly hindered the further applications of Pd. With the development of state-of-the-art technology, heterogeneous catalysis employing reusable and recyclable supported Pd nanoparticles (PdNPs) has developed as an efficient strategy that carries the promise of industrialized and sustainable syntheses of high-value chemicals (Djakovitch and Felpin, 2014; Pla and Gómez, 2016; Dhakshinamoorthy et al., 2019; Hong et al., 2020). The Pd/γ-Al_2_O_3_ as a promising heterogeneous catalyst has been utilized to construct various C–C and C–X bonds (Suhelen et al., 2014; Chen et al., 2013; Bao et al., 2016a; Zhang et al., 2015).

**SCHEME 1:**
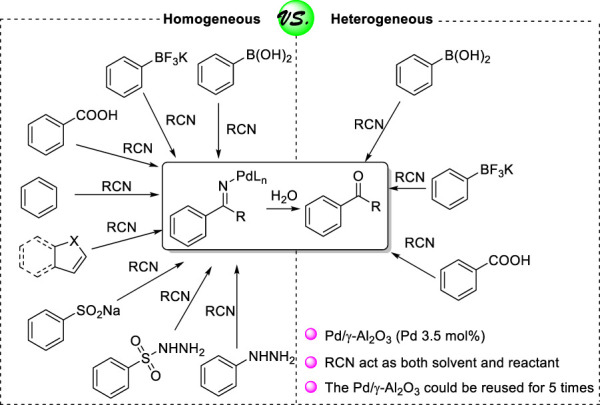
General methods to synthesize ketone via carbopalladation of nitrile.

The ketone moiety, a common structural element, occurring in numerous pharmaceutical molecules, natural products, and other biological function molecules, was regarded as an important building block and useful synthons in organic synthesis and natural products (Surburg and Panten, 2006). In view of the importance of ketone structure, it is at first glance remarkable that the construction of this omnipresent skeleton has been extensively studied, such as the oxidation of secondary alcohols and Friedel–Crafts acylation of aromatic compounds in the presence of an acid, while the addition of oxidants or acid result in functional group tolerance problem (Olah, 1973; Tojo and Fernandez, 2006; Sartori and Maggi, 2009; Dobereiner and Crabtree, 2010). Transition-metal-catalyzed reactions provided a blueprint to efficiently synthesize ketones under mild reaction conditions with a simple operation (Takemiya and Hartwig, 2006; Ruan et al., 2008; Desai et al., 2010). Thereinto, carbopalladation of nitriles with various coupling partners offered a particularly powerful approach to assembling ketones via the ketamine intermediate because nitriles, cheap and commercially available feedstocks, are abundant surrogates for organic synthesis. In 1999, seminal studies from Larock presented that the use of a palladium catalyst can indeed produce a variety of ketones from the corresponding arenes or arylboronic acids with nitrile (Larock et al., 1999). Following this pioneering work, a number of related works were reported, and a broad range of coupling partners could react with nitriles via intermolecular carbopalladation, such as potassium aryltrifluoroborates (Chen et al., 2013; Wang et al., 2013), benzoic acid (Lindh et al., 2010), heteroarene (Jiang and Wang, 2013; Jiang and Wang, 2014), sodium arylsulfinates (Liu et al., 2011; Skillinghaug et al., 2014), arylhydrazines (Cheng et al., 2017), and arylsulfonyl hydrazides (Meng et al., 2018) (Scheme 1). Inspired by our previous work that supported PdNPs as suitable catalysts for pyridine synthesis via tandem carbopalladation/cyclization of acetonitrile, arylboronic acids, and aldehydes (Bai et al., 2021), we recently questioned whether supported PdNPs could be an ideal alternative heterogeneous catalyst for the construction of ketone under mild condition. To the best of our knowledge, general methods for employing heterogeneous catalysts to synthesize ketone via carbopalladation of nitrile are currently unknown.

Herein, we prepared a variety of supported PdNPs catalysts that were found to be efficient heterogeneous catalysts for the construction of ketone via intermolecular carbopalladation of nitrile with various coupling partners, which could be reused and recycled five times. The TEM characterization showed that the mean diameter of PdNPs for the heterogeneous catalyst, supported Pd/γ-Al_2_O_3_, is 3.00 nm, and no conspicuous agglomeration of PdNPs was observed during the reaction. The XPS revealed that this reaction proceeds via the Pd^0^/Pd^II^ catalytic cycle.

## Materials and Methods

### Catalyst Preparation

The PdNPs on *γ*-Al_2_O_3_ were prepared by a modified impregnation-reduction method. (Bao et al., 2016a; Bao et al., 2016b): γ-Al_2_O_3_ powder (0.97 g) was suspended in distilled water (50 ml), followed by the addition of aqueous PdCl_2_ solution (0.01 M, 28.2 ml), and stirred at room temperature. Then, aqueous L-lysine solution (0.03 M, 1 ml) was added to the aforementioned mixture. Subsequently, NaOH aqueous solution (0.1 M) was added to the mixture to adjust the pH to 7. Whereafter, aqueous NaBH_4_ solution (0.35 M, 4.5 ml) was added dropwise within 10 min while vigorously stirring. The mixture was aged for 24 h, and the solid was separated by centrifugation, washed with distilled water four times and ethanol once, and dried at 80°C. The dried solid was used directly as the catalyst.

### Activity Test

The reaction was carried out in a 25 ml dry reaction tube. 4-Methylphenylboronic acid 1a (27.2 mg, 0.2 mmol), acetonitrile (1 ml), 3 wt.% Pd/γ-Al_2_O_3_ (25 mg), L_1_ (6.5 μL, 20 mol%), and H_2_O (200 μL) were added in turn to the reaction tube and reflux at 120°C for 48 h. After the reaction is complete, the reaction is cooled to room temperature. The product was purified by thin-layer chromatography.

### Catalyst Recycle Experiment

After each reaction cycle, the mixture is layered through a centrifuge, and the separated 3 wt.% Pd/γ-Al_2_O_3_ was washed thoroughly with distilled water until pH = 7 and then washed twice with anhydrous ethanol followed by centrifugal separation and drying at 40°C for 12 h. The recovered catalyst is used for the next cycle.

### Gram-Scale Synthesis

4-Methylphenylboronic acid 1a (1.00 g, 7.35 mmol), CH_3_CN:H_2_O = 5:1 (44 ml), 3 wt.% Pd/γ-Al_2_O_3_ (0.918 g), and L_1_ (229.5 μL, 1.47 mmol) were added in a round-bottom flask, and the reaction was carried out at 120°C for 48 h in an oil bath under air condition. The resulting mixture was cooled to room temperature and evaporated in vacuo. The residue was purified by flash column chromatography (silica gel, ethyl acetate/petroleum ether = 1:10 as an eluent) to afford the desired product 2a (0.561 g, 57% yield).

## Results and Discussion

We started our investigation by subjecting 4-methylphenylboronic acid 1a in the presence of a Pd catalyst and ligand in MeCN and H_2_O (Table 1, and Supplementary Tables S1, S2). Firstly, we investigated Pd(OAc)_2_ or Pd(PPh_3_)_4_ as a homogeneous catalyst and 6-methyl-2,2′-bipyridine (L_1_) as a ligand that was an efficient ligand in Larhed works, and the product was formed in 63% or 57% yield, respectively (entries 1 and 2) (Lindh et al., 2010). Due to the success of our previous works, we considered whether supported PdNPs could act as an alternative catalyst and found that 3 wt.% Pd/γ-Al_2_O_3_ afforded the desired product in 92% yield (entry 3). Regretfully, commercially available 5 wt.% Pd/C gave 2a in the yield of 53% (entry 8). Next, different ligands were tested, and L_1_ was proved to be optimal for this reaction (entries 5–12). Any fluctuation in the Pd loading from 3 wt.% resulted in a decrease in reaction yields (entries 13–16). Notably, the reaction proceeded in the absence of water with a significant decrease in yield (entry 17). This indicated that water is critical to the success of the reaction, which may be attributed to the fact that water could increase the solubility of phenylboronic acid and contribute to the hydrolysis of ketamine intermediate.

**TABLE 1:** The reaction optimization of 4-methylphenylboronic acid^a^ ^,^ ^b^.

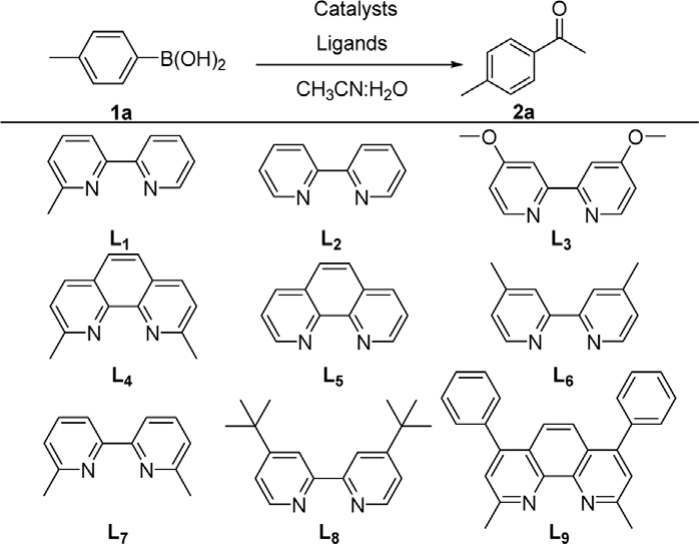			
**Entry**	**Pd source**	**Ligands**	**Yield (%)** ^b^
1^c^	Pd(OAc)_2_	L_1_	63
2^c^	Pd(PPh_3_)_4_	L_1_	57
3	3 wt.% Pd/γ-Al_2_O_3_	L_1_	92
4	5 wt.% Pd/C	L_1_	53
5	3 wt.% Pd/γ-Al_2_O_3_	L_2_	45
6	3 wt.% Pd/γ-Al_2_O_3_	L_3_	57
7	3 wt.% Pd/γ-Al_2_O_3_	L_4_	52
8	3 wt.% Pd/γ-Al_2_O_3_	L_5_	57
9	3 wt.% Pd/γ-Al_2_O_3_	L_6_	46
10	3 wt.% Pd/γ-Al_2_O_3_	L_7_	30
11	3 wt.% Pd/γ-Al_2_O_3_	L_8_	72
12	3 wt.% Pd/γ-Al_2_O_3_	L_9_	30
13	1 wt.% Pd/γ-Al_2_O_3_	L_1_	49
14	2 wt.% Pd/γ-Al_2_O_3_	L_1_	61
15	4 wt.% Pd/γ-Al_2_O_3_	L_1_	60
16	5 wt.% Pd/γ-Al_2_O_3_	L_1_	56
17^d^	3 wt.% Pd/γ-Al_2_O_3_	L_1_	18

a Reaction conditions: 1a (0.2 mmol), catalyst (25 mg), ligand (20 mol%), solvent (CH3CN: H_2_O = 5:1, 1.2 ml), and 120°C, 48 h.

b Isolated yield.

c Catalyst (10 mol%).

d Without water.

Having established the optimized reaction conditions for Pd/γ-Al_2_O_3_ catalyzed intermolecular carbopalladation of nitrile with arylboronic acids to synthesize ketone, we next explored the scope of arylboronic acids (Table 2). Electron-rich and electron-deficient arylboronic acids were tolerated under this reaction condition, affording the desired product in moderate to excellent yield (2a–2k). In general, substrates containing electron-donating groups worked better than those with electron-withdrawing groups (2k). Unfortunately, substrates with a strong electron-withdrawing group (such as 4-F) failed to afford the product. Sterically demanding substrates were found to be significantly less active for this protocol, providing products in diminished yield (2m and 2n). To our delight, naphthalen-1-yl boronic acid and (1*H*-indol-4-yl) boronic acid served as suitable substrates for this reaction.

**TABLE 2:** Scope of phenylboronic acid^a^ ^,^ ^b^.

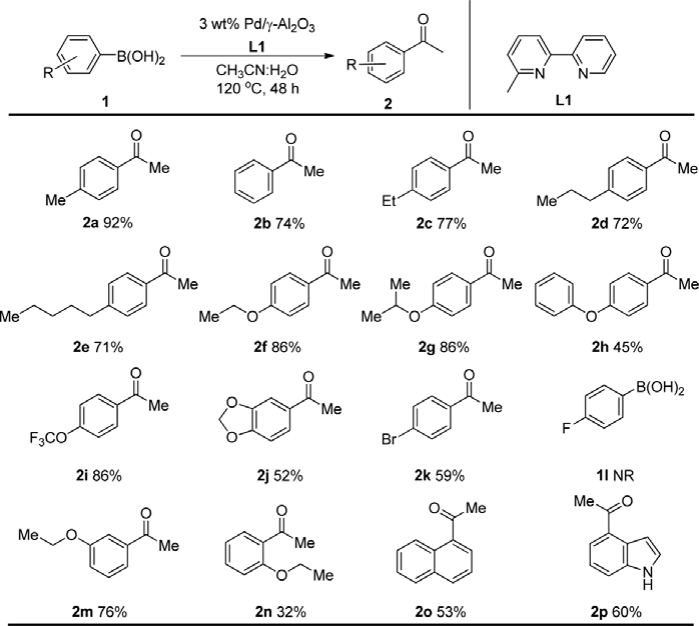

a Reaction conditions: 1a (0.2 mmol), 3 wt.% Pd/γ-Al_2_O_3_ (25 mg), L_1_ (20 mol%), CH_3_CN: H_2_O = 5:1 (1.2 ml), and 120 °C, 48 h.

b Isolated yield.

**FIGURE 1:**
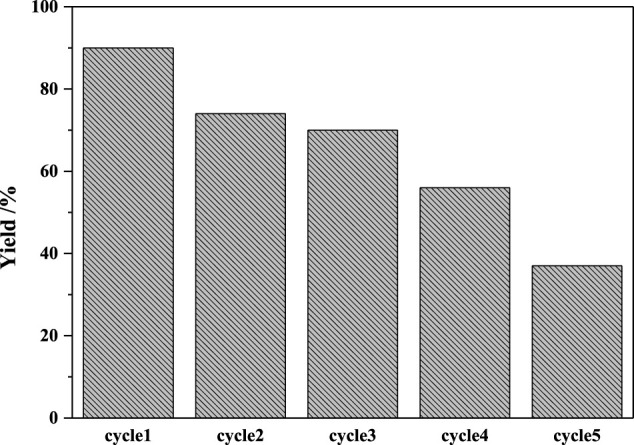
Recyclability of catalysts.

The recyclability of the catalyst was examined in intermolecular carbopalladation of nitriles with 4-methylphenylboronic acid under the optimal reaction condition. As illustrated in Figure 1, Pd/γ-Al_2_O_3_ could be recovered by centrifugation and reused five times with somewhat deactivation of the catalyst. The ICP-MS analysis revealed 19.08 ppm Pd leaching in the reaction solution, which may lead to the decrease in yield.

This protocol appeared to be performed mainly by heterogeneous pathways, which were demonstrated by a hot filtration test. After filtering the 3 wt.% PdNPs/γ-Al_2_O_3_ in the middle of the reaction, no further reaction was observed precluding the possibility of homogeneous catalysis, which was confirmed by the yield of 1a, as shown in Table 3.

**TABLE 3:** The results of the hot filtration test.

Time (hour)	Yield (%)^*a*^
0	0
2	15.4
4 (after hot filtration)	15.9

Building on our strategy to synthesize ketone employing Pd/γ-Al_2_O_3_, we sought to realize the intermolecular carbopalladation reaction of acetonitrile with potassium trifluoro (*p*-tolyl)borate. As shown in Table 4, a broad range of potassium phenyltrifluoroborate that exhibit diverse electronic properties readily participate in this reaction. This reaction of potassium trifluoro (*p*-tolyl)borate is tolerant to substrates that incorporate various electron-donating substituents in different sites, such as alkyl, alkoxy (entries 1–7, 2a-2b, and 2q-2u), and -OH (entry 8, 2v). A preference for the electron-donating groups on the potassium phenyltrifluoroborate was observed for this reaction. Pleasingly, compared with arylboronic acids, potassium phenyltrifluoroborate with 4-F could also give a corresponding product in a moderate yield of 37%. Gratifyingly, the substrate with naphthalene was suitable for this reaction, delivering products in 37% and 51% yield, respectively (entries 14 and 15, 2y, and 2o).

**TABLE 4:** Scope of potassium phenyltrifluoroborate^a^ ^,^ ^b^.

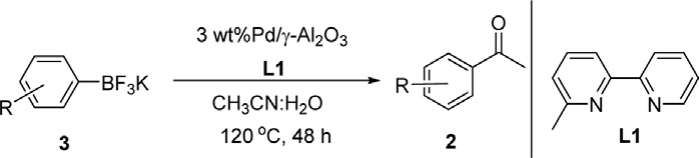			
**Entry**	**Substrate**	**Product**	**Yield (%)**
1	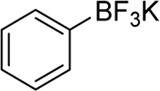 3a	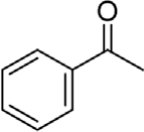 2b	70
2	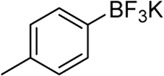 3b	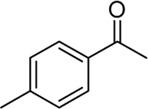 2a	90
3	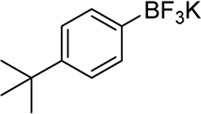 3c	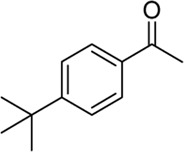 2q	55
4	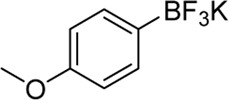 3d	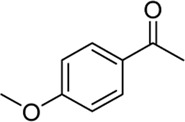 2r	81
5	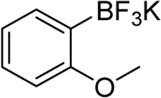 3e	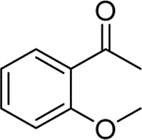 2s	61
6	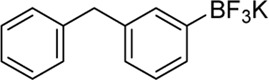 3f	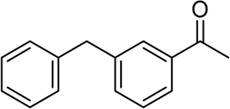 2t	56
7	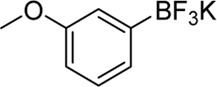 3g	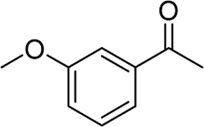 2u	70
8	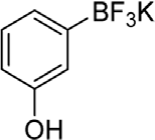 3h	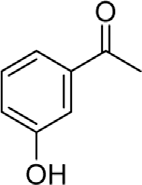 2v	79
9	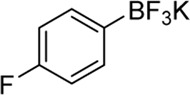 3i	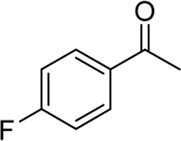 2w	37
10	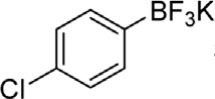 3j	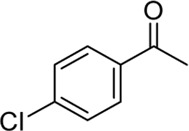 2x	39
11	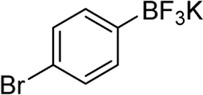 3k	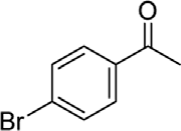 2m	44
12	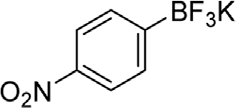 3l		NR
13	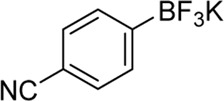 3m		NR
14	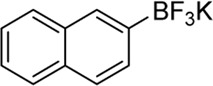 3n	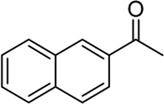 2y	37
15	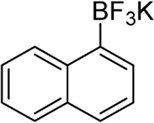 3o	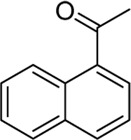 2o	51

a Reaction conditions: 3a (0.2 mmol), 3 wt.% Pd/γ-Al_2_O_3_ (25 mg), L_1_ (20 mol%), CH_3_CN: H_2_O = 5:1 (1.2 ml), and 120°C, 48 h.

b Isolated yield.

**SCHEME 2:**
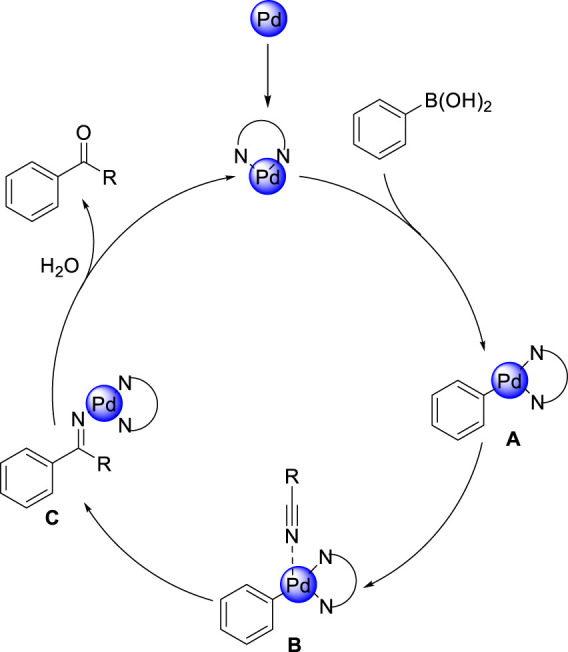
The proposed mechanism.

To gain some insight into the mechanism of the present carbopalladation of nitrile, the reaction solution of 4-methylphenylboronic acid and potassium trifluoro (*p*-tolyl)borate was detected by GC-MS after the end of the reactions, respectively (see Supplementary Material). A small amount of 4,4′-dimethyl-biphenyl was found as a byproduct both in those two reactions, which demonstrated that the transmetalation of the palladium catalyst and aryboronic acid occurred firstly. Based on the literature and our previous work (Chen et al., 2013; Wang et al., 2013; Bai et al., 2021), the proposed mechanism of the present reaction is presented in Scheme 2. The reaction began with transmetalation of the palladium catalyst and aryboronic acid to form intermediate A. Subsequently, the cyano group coordinated with intermediate A followed by the 1,2-carbopalladation of the cyano group to furnish the intermediate C that was hydrolyzed to deliver the desired ketone.

**SCHEME 3:**
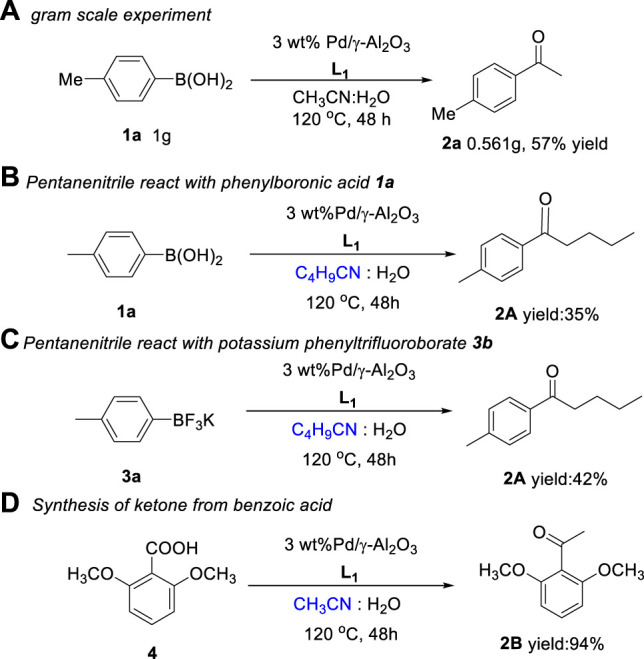
Synthetic application.

In order to investigate the practical application of this newly heterogeneous catalytic reaction in organic synthesis, we carried out a scaleup experiment and isolated desired ketone with a slight decrease in yield (Scheme 3A). Gratifyingly, pentanenitrile was found to be compatible under this reaction condition, which could react with both arylboronic acid and potassium phenyltrifluoroborate to give the corresponding products 3A in 35% and 42% yield, respectively (Scheme 3B,C). Perhaps even more notable, the use of benzoic acid with steric hindrance has enabled the synthesis of sterically demanding disubstituted ketone, which further extends the scope of ketones (Scheme 3D).

**FIGURE 2:**
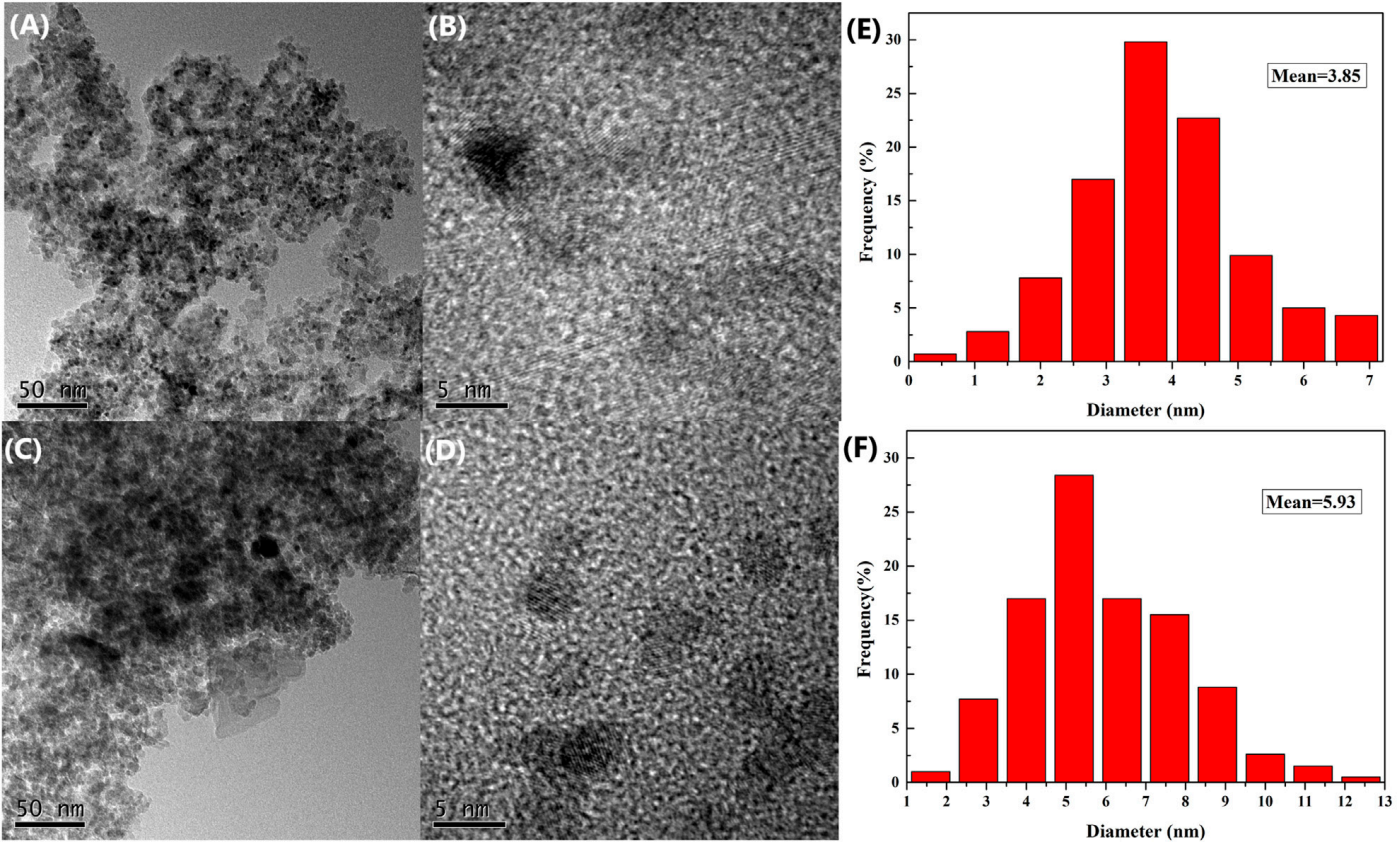
TEM images of 3 wt.% Pd/γ-Al_2_O_3_: **(A,B)** fresh catalyst; **(C,D)** used (after first recycling) catalyst; **(E,F)** PdNP size distributions of fresh and used catalysts, respectively.

To obtain a better understanding of the catalyst of the reaction, the fresh and used catalysts were analyzed by transmission electron microscopy (TEM) and X-ray photoelectron spectroscopy (XPS). The transmission electron microscopy (TEM) images of the fresh and used 3 wt.% Pd/γ-Al_2_O_3_ catalysts are given in Figure 2. The PdNPs are distributed uniformly on the surface of γ-Al_2_O_3_ (see Figures 2A–D). The TEM showed the Pd particles 3.23 and 3.74 nm in diameter for fresh and used catalysts, respectively (Figures 2E,F). Although the used Pd particle size is slightly larger, the PdNPs were still distributed evenly, and no apparent agglomeration was observed.

**FIGURE 3:**
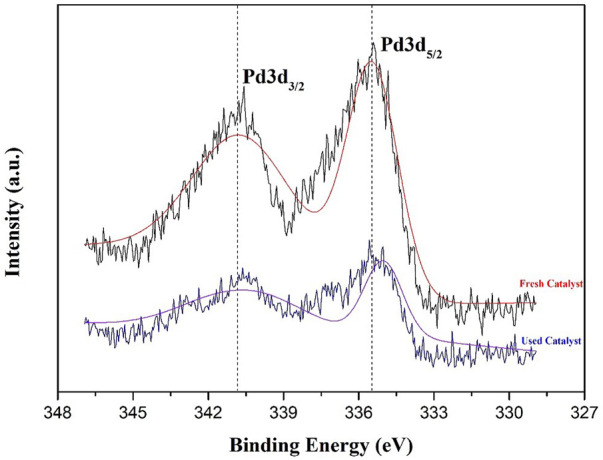
The XPS analysis of fresh (red) and used (blue) supported PdNPs.

The valence state of fresh and used supported PdNPs was confirmed by the X-ray photoelectron spectroscopy (XPS) analysis, which confirm that Pd exists in the metallic state on the γ-Al_2_O_3_ supports before and after the reaction. As shown in Figure 3, the binding energies of Pd 3d_5/2_ and Pd 3d_3/2_ for the fresh catalyst are 335.43 and 340.58 eV, respectively, and those for the used catalyst (after first recycling) are 335.03 and 340.63 eV, respectively, which is in line with the literature. It is shown that Pd^0^ is the active center in the catalytic cycle.

## Conclusion

In conclusion, we have reported the first example of the direct synthesis of aromatic ketones via intermolecular carbopalladation of aliphatic nitriles with organoboron compound employing Pd/γ-Al_2_O_3_ as a heterogeneous catalyst. This catalyst represents excellent catalytical activity and could be reused and recycled five times. The XPS analysis indicated that Pd^0^ was active species for this reaction. Potassium phenyltrifluoroborate and benzoic acid were also viable substrates for this protocol. This methodology provides a mild and cost-effective strategy for the efficient synthesis of ketones.

## Data Availability

The original contributions presented in the study are included in the article/Supplementary Material, further inquiries can be directed to the corresponding author.
