# Supplementary material for: Supported Palladium Nanoparticles Catalyzed Intermolecular Carbopalladation of Nitriles and Organoboron Compounds
Source: Front Chem. 2022 May 9;10:855850. doi: 10.3389/fchem.2022.855850 (PMC9124771; doi:10.3389/fchem.2022.855850)

Supplementary Material

Supported Palladium Nanoparticles Catalyzed Intermolecular Carbopalladation of Nitriles and Organoboron Compound

Xin Liu,[1†] Dan Liu,[1†] Tegshi Muschin,[1] Agula Bao,[1] Chaolumen Bai,[1] Yong-Sheng Bao*[1]

[1] Inner Mongolia Key Laboratory of Green catalysis, Inner Mongolia Normal University, College of Chemistry and Environmental Science, Hohhot, China

Correspondence:
Yong-Sheng Bao

sbbys197812@163.com

Contents

1 General Information 2

2 Optimization of reaction conditions and the data analysis of yields 2

3 GC-MS analysis of the reaction of 1a 5

4 GC-MS analysis of the reaction of 3a 6

5 Characterization Data for the Products....................................8

6 ^{1}H NMR and ^{13}C NMR Spectra of the Products 15

1 General Information

All the reagents were purchased from Aladdin and Alfa without further purification. Thin layer chromatography (TLC) was performed on pre-coated silica gel GF254 plates. The ^{1}H NMR and ^{13}C NMR spectra were measured on a 600 MHz Bruker Avance III nuclear magnetic resonance spectrometer, using $CDCl_3$ as the solvent with tetramethylsilane (TMS) as the internal standard. Chemical shifts (δ) are expressed in ppm. The structures of known compounds were further corroborated by comparing their ^{1}H NMR data with those of literature. The GC-MS analysis was detected on a Thermo DSQ-II with a DB-5 column. The TEM images were recorded on a JEM-2100 transmission electron microscope employing an accelerating voltage of 200 kV. The samples were suspended in ethanol and dried on holey carbon-coated Cu grids. The X-ray photoelectron spectroscopy (XPS) was recorded on a Kratos Amicus of British equipped with taper anode Mg Kα radiation. The C1s hydrocarbon peak at 284.60 eV was used as an internal standard for the correction of binding energies. ICP-MS analysis was conducted at the Agilent 7700ce ICP-MS spectrometer.

2 Optimization of reaction conditions and the data analysis of yields

Table S1. Screening of the Reaction Temperature

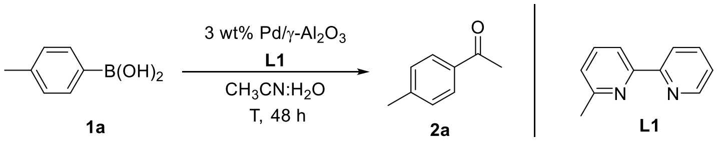

Entry	Temperature	Yield/% [a,b]
1	80℃	43
2	90℃	50
3	100℃	70
4	110℃	82
5	120℃	92
6	130℃	75

[a] Reaction conditions: **1a** (0.2 mmol), Catalyst (25 mg), Ligand (20 mol%), Solvent (CH_3CN: H_2O = 5:1, 1.2 mL), 120 °C, 48 h. [b] isolated yield

Table S2. Screening of the Reaction Time

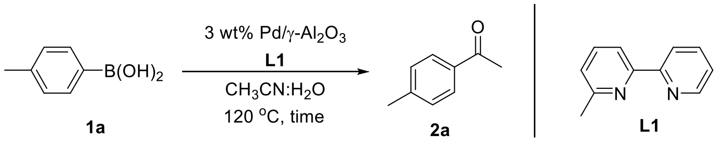

Entry	Time (h)	Yield (%) [a,b]
1	12	18
2	24	48

3	36	50
4	48	92
5	72	60

[a] Reaction conditions: **1a** (0.2 mmol), Catalyst (25 mg), Ligand (20 mol%), Solvent (CH_3CN: H_2O = 5:1, 1.2 mL), 120 °C. [b] isolated yield

Table S3 Repeated experiments of phenylboronic acid

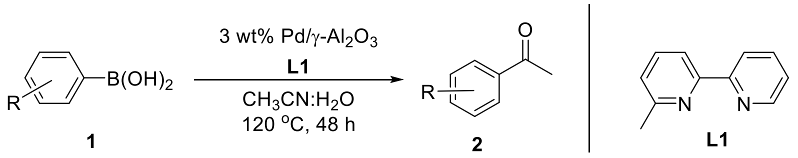

Product	Number of repetitions	Yield [a,b]
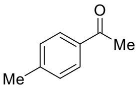 **2a**	10	88.9-94.8%
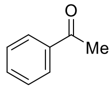 **2b**	10	72.9-75.8%
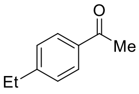 **2c**	2	77.1%，77.6%
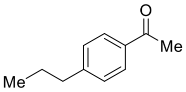 **2d**	2	72.0%，72.8%
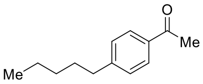 **2e**	2	70.8%，71.3%
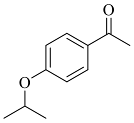 **2g**	2	86.3%，85.9%
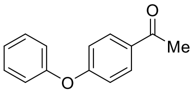 **2h**	2	45.8%，44.9%
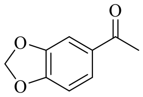 **2j**	2	52.8%，51.0%
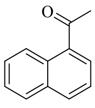 **2o**	2	53.5%，51.8%
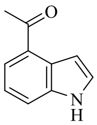 **2p**	2	60.7%，60.2%

[a] Reaction conditions: **1a** (0.2 mmol), 3 wt% Pd/ γ -Al_2O_3 (25 mg), **L₁** (20mol%), CH_3CN : H_2O = 5:1 (1.2 mL), 120 °C, 48 h. [b] isolated yield.

Table S4. The t-test analysis of the carbopalladation reaction of phenylboronic acid

Product	entry	Yield	entry	Yield	P
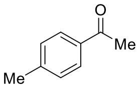 **2a**	1	90.2	6	93.4	0.30284
	2	89.5	7	90.9	
	3	91.3	8	91.8	
	4	88.9	9	92.7	
	5	94.8	10	92.1	
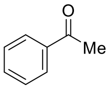 **2b**	1	73.6	6	75.8	0.514986
	2	73.3	7	74.1	
	3	74.7	8	72.9	
	4	73.8	9	75	
	5	74.4	10	73.9	

Table S5 Repeated experiments of phenyltrifluoroborate

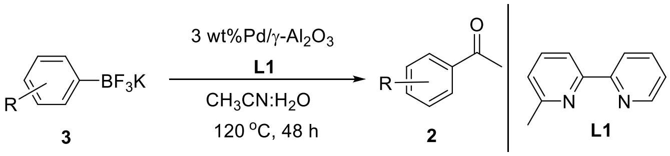

Product	Number of repetitions	Yield [a, b]
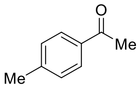 **2a(3a)**	8	87.8-91.6%
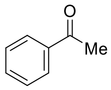 **2b(3b)**	10	67.6-73.5%
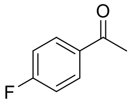 **2w(3i)**	2	37.8%，37.0%
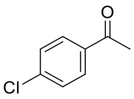 **2x(3j)**	2	39.2%，38.9%
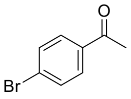 **2m(3k)**	2	43.5%，43.8%
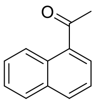 **2o(3o)**	2	51.7%，51.1%
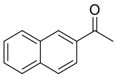 **2y(3n)**	2	37.9%，36.9%

[a] Reaction conditions: **1a** (0.2 mmol), 3 wt% Pd/ γ -Al_2O_3 (25 mg), **L1** (20mol%), CH_3CN : H_2O = 5:1 (1.2 mL), 120 °C, 48 h. [b] isolated yield.

Table S6. The t-test analysis of the the carbopalladation reaction of phenyltrifluoroborate

Product	entry	Yield	entry	Yield	P
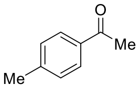 **2a**	1	87.8	5	91.2	0.36511138
	2	91.6	6	90.6	
	3	89.2	7	91	
	4	90.6	8	90.1	
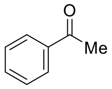 **2b**	1	70	6	70.3	0.153502
	2	71.2	7	69.8	
	3	71.3	8	70.5	
	4	69.6	9	67.6	
	5	73.5	10	70.5	

3 GC-MS analysis of the reaction of 1a

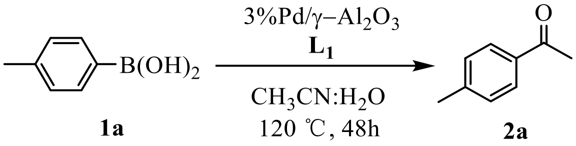

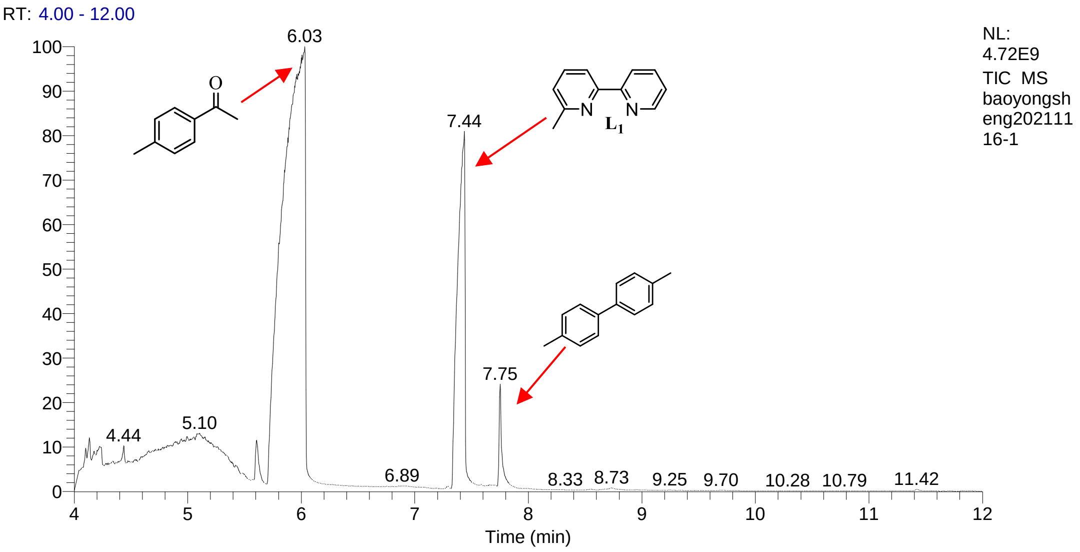

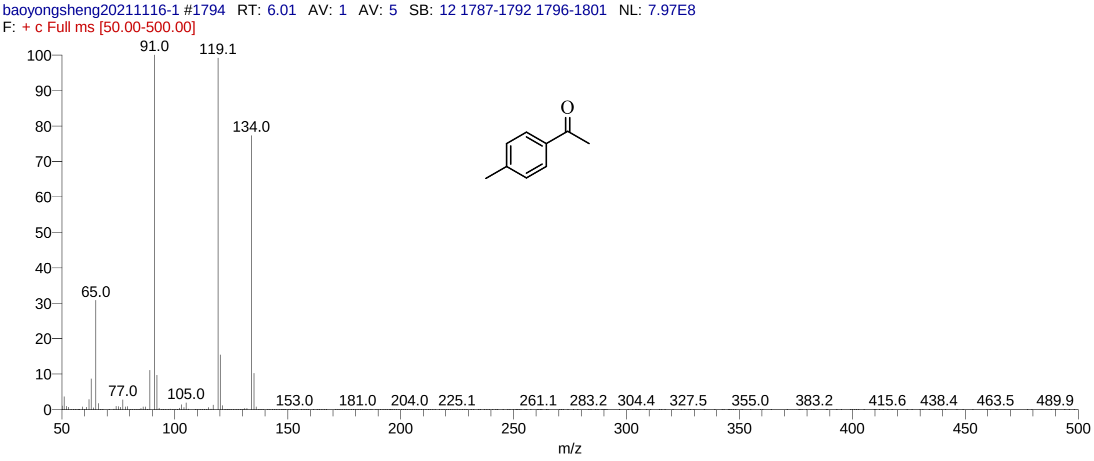

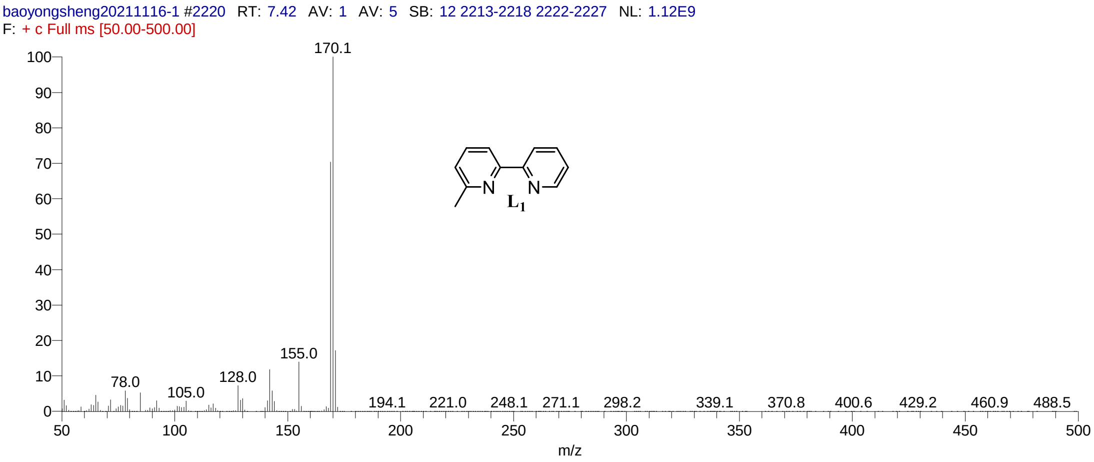

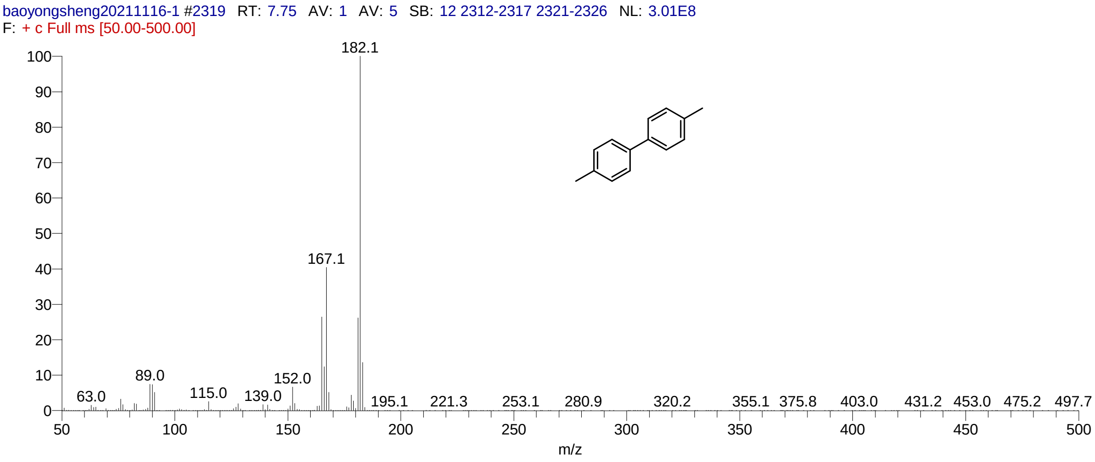

4 GC-MS analysis of the reaction of 3a

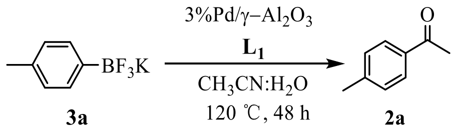

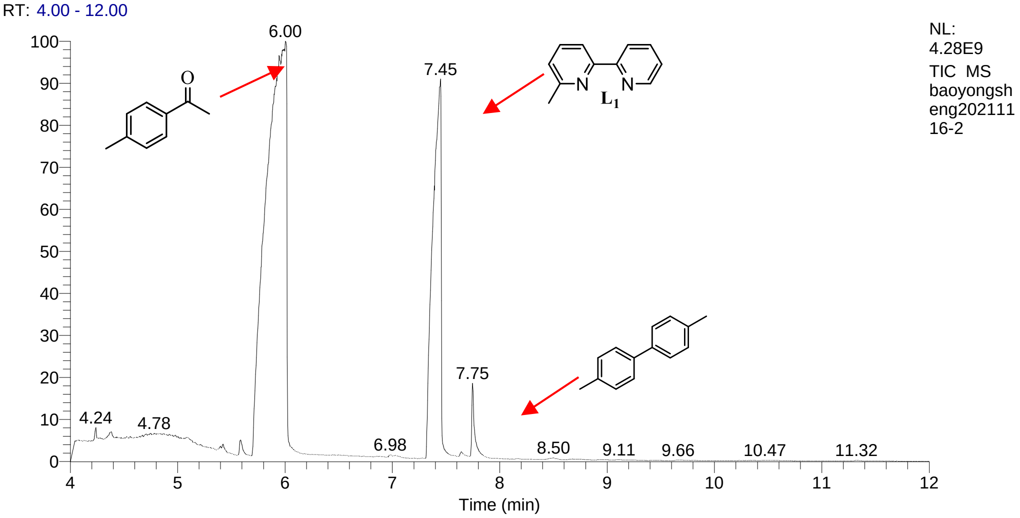

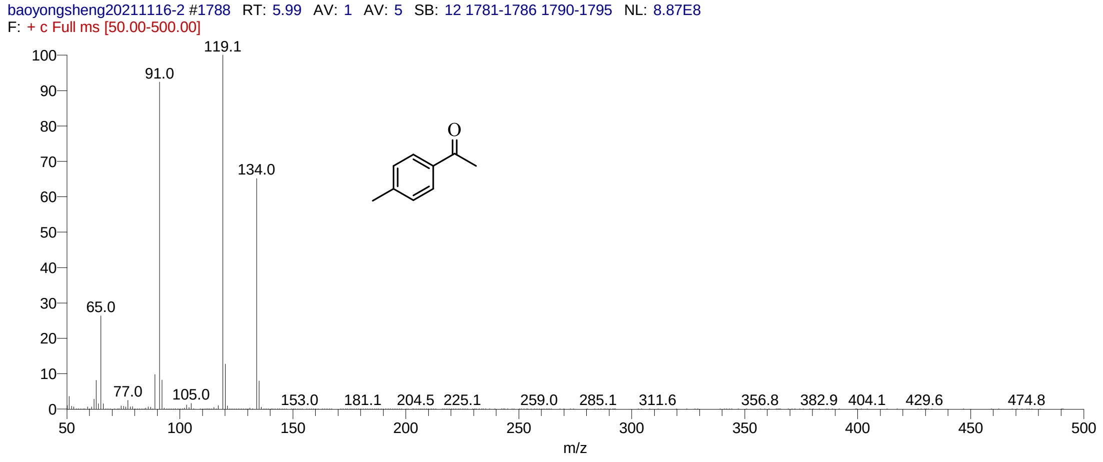
baoyongsheng20211116-2 #1788 RT: 5.99 AV: 1 AV: 5 SB: 12 1781-1786 1790-1795 NL: 8.87E8
F: + c Full ms [50.00-500.00]
119.1
91.0
134.0
65.0
77.0
105.0
153.0 181.1 204.5 225.1 259.0 285.1 311.6 356.8 382.9 404.1 429.6 474.8
O
m/z

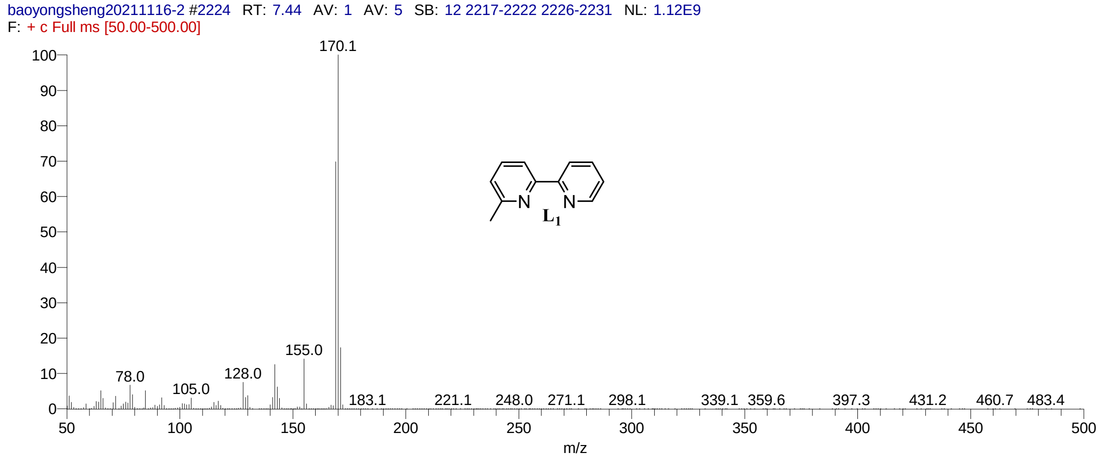
baoyongsheng20211116-2 #2224 RT: 7.44 AV: 1 AV: 5 SB: 12 2217-2222 2226-2231 NL: 1.12E9
F: + c Full ms [50.00-500.00]
170.1
155.0
128.0
105.0
78.0
183.1 221.1 248.0 271.1 298.1 339.1 359.6 397.3 431.2 460.7 483.4
N
N
L_1
m/z

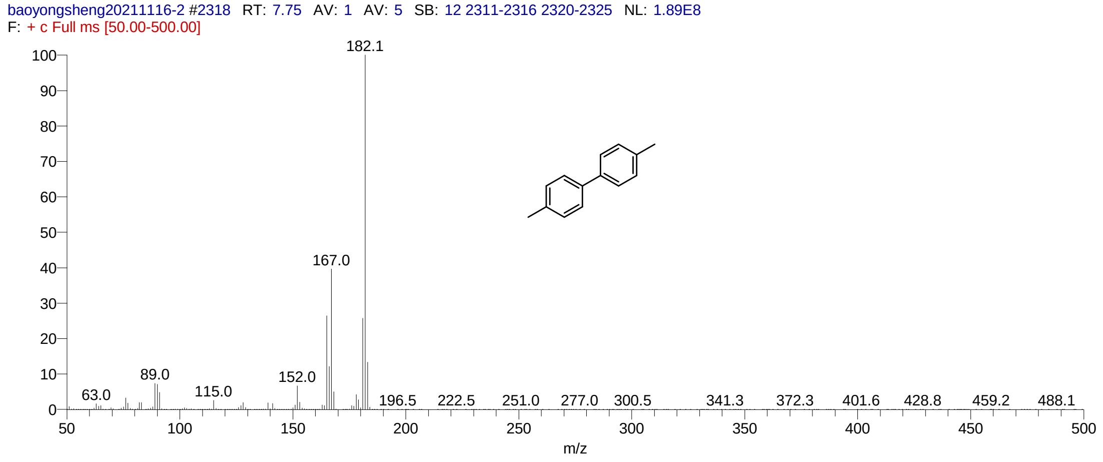
baoyongsheng20211116-2 #2318 RT: 7.75 AV: 1 AV: 5 SB: 12 2311-2316 2320-2325 NL: 1.89E8
F: + c Full ms [50.00-500.00]
182.1
167.0
152.0
89.0
115.0
63.0
196.5 222.5 251.0 277.0 300.5 341.3 372.3 401.6 428.8 459.2 488.1
m/z

5 Characterization Data for the Products

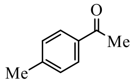

1-(p-tolyl)ethan-1-one **2a**. (Li et al., 2020). . Yield: 92% (M=134.18, 24.6 mg). ^{1}H NMR (600 MHz, $CDCl_3$) δ 7.86 (d, *J* = 7.9 Hz, 2H), 7.26 (d, *J* = 7.9 Hz, 2H), 2.58 (s, 3H), 2.41 (s, 3H); ^{13}C NMR (151 MHz, $CDCl_3$) δ 197.9, 143.9, 134.7, 129.2, 128.5, 26.5, 21.6.

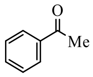

Acetophenone **2b**. (Wang et al., 2013). Yield: 74% (M=120.15, 17.8 mg). ^{1}H NMR (600 MHz, $CDCl_3$) δ 7.96 (d, *J* = 7.5 Hz, 2H), 7.57 (t, *J* = 7.4 Hz, 1H), 7.47 (t, *J* = 7.7 Hz, 2H), 2.61 (s, 3H); ^{13}C NMR (151 MHz, $CDCl_3$) δ 198.2, 137.2, 133.1, 128.6, 128.3, 26.6.

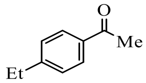

1-(4-ethylphenyl)ethan-1-one **2c**. (Hu et al., 2019). Yield: 77% (M=148.20, 22.8 mg). ^{1}H NMR (600 MHz, $CDCl_3$) δ 7.89 (d, *J* = 7.7 Hz, 2H), 7.28 (d, *J* = 7.9 Hz, 2H), 2.71 (q, *J* = 7.6 Hz, 2H), 2.58 (s, 3H), 1.26 (t, *J* = 7.6 Hz, 3H); ^{13}C NMR (151 MHz, $CDCl_3$) δ 197.9, 150.1, 134.9, 128.6, 128.1, 28.9, 26.6, 15.2.

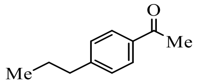

1-(4-propylphenyl)ethan-1-one **2d**. (Suta and Turks, 2018). Yield: 72% (M=162.23 23.4 mg). ^{1}H NMR (600 MHz, $CDCl_3$) δ 7.88 (d, *J* = 8.0 Hz, 2H), 7.26 (d, *J* = 8.0 Hz, 2H), 2.64 (t, *J* = 7.6 Hz, 2H), 2.58 (s, 3H), 1.66 (m, 2H), 0.95 (t, *J* = 7.3 Hz, 3H); ^{13}C NMR (151 MHz, $CDCl_3$) δ 198.0, 148.6, 135.0, 128.7, 128.5, 38.0, 26.6, 24.2, 13.8.

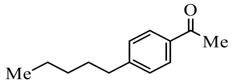

1-(4-pentylphenyl)ethan-1-one **2e**. (Sun et al., 2019). Yield: 71% (M=190.29, 27.0 mg). ^{1}H NMR (600 MHz, $CDCl_3$) δ 7.88 (d, *J* = 8.0 Hz, 2H), 7.26 (d, *J* = 8.0 Hz, 2H), 2.67-2.64 (t, *J*=7.7 Hz, 2H), 2.58 (s, 3H), 1.66 – 1.60 (m, 2H), 1.38 – 1.28 (m, 4H), 0.89 (t, *J* = 6.8 Hz, 3H); ^{13}C NMR (151 MHz, $CDCl_3$) δ 197.9, 148.8, 134.9, 128.6, 128.5, 36.0, 31.4, 30.8, 26.5, 22.5, 14.0.

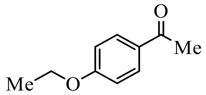

1-(4-ethoxyphenyl)ethan-1-one **2f**. (Thiruvengetam et al., 2019). Yield: 86% (M=164.20, 28.2 mg). ^{1}H NMR (600 MHz, $CDCl_3$) δ 7.92 (d, *J* = 8.7 Hz, 2H), 6.91 (d, *J* = 8.7 Hz, 2H), 4.10 (q, *J* = 7.0 Hz, 2H), 2.55 (s, 3H), 1.44 (t, *J* = 7.0 Hz, 3H);^{13}C NMR (151 MHz, $CDCl_3$) δ 196.8, 162.9, 130.6, 130.2, 114.1, 63.8, 26.3, 14.8.

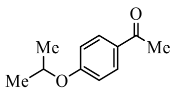

1-(4-isopropoxyphenyl)ethan-1-one **2g**. (Qin et al., 2020). Yield: 86% (M=178.23, 30.7 mg). ^{1}H NMR (600 MHz, $CDCl_3$) δ 7.92 (d, *J* = 7.7 Hz, 2H), 6.90 (d, *J* = 7.8 Hz, 2H), 4.67 – 4.61 (m, 1H), 2.54 (s, 3H), 1.36 (d, *J* = 6.1Hz, 6H); ^{13}C NMR (151 MHz, $CDCl_3$) δ 196.7, 162.0, 130.6, 129.9, 115.0, 70.1, 26.3, 21.9.

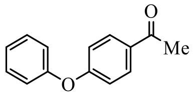

1-(4-phenoxyphenyl)ethan-1-one **2h**. (Bhatt et al., 2020). Yield: 45% (M=212.24, 19.0 mg). ^{1}H NMR (600 MHz, $CDCl_3$) δ 7.94 (d, *J* = 8.8 Hz, 2H), 7.39 (t, *J* = 7.9 Hz, 2H), 7.20 (t, *J* = 7.4 Hz, 1H), 7.09 – 7.05 (m, 2H), 7.00 (d, *J* = 8.7 Hz, 2H), 2.57 (s, 3H); ^{13}C NMR (151 MHz, $CDCl_3$) δ 196.73, 162.00, 155.5, 131.9, 130.6, 130.1, 124.6, 120.2, 117.3, 26.5.

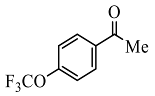

1-(4-(trifluoromethyl)phenyl)ethan-1-one **2i**. (Xie et al., 2021). Yield: 86% (M=188.15, 15.1 mg). ^{1}H NMR (600 MHz, $CDCl_3$) δ 8.01 (d, *J* = 8.6 Hz, 2H), 7.29 (d, *J* = 8.6 Hz, 2H), 2.61 (s, 3H); ^{13}C NMR (151 MHz, $CDCl_3$) δ 196.5, 152.7, 135.4, 130.3, 120.3(q, *J* = 258.8 Hz, 1C), 26.6.

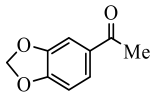

1-(benzo[d][1,3]dioxol-5-yl)ethan-1-one **2j**. (Wang et al., 2019). Yield: 52% (M=164.16, 18.2 mg). ^{1}H NMR (600 MHz, $CDCl_3$) δ 7.55 (dd, *J* = 8.1, 1.0 Hz, 1H), 7.44 (s, 1H), 6.85 (d, *J* = 8.1 Hz, 1H), 6.05 (s, 2H), 2.54 (s, 3H); ^{13}C NMR (151 MHz, $CDCl_3$) δ 196.2, 151.8, 148.2, 132.1, 124.8, 108.0, 107.8, 101.8, 26.5.

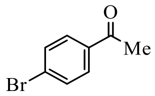

1-(4-bromophenyl)ethan-1-one **2k**. (Hao et al., 2019). Yield: 59% (M=199.04, 23.5 mg). ^{1}H NMR (600 MHz, $CDCl_3$) δ 7.82 (d, *J* = 8.4 Hz, 2H), 7.61 (d, *J* = 8.4 Hz, 2H), 2.59 (s, 3H);^{13}C NMR (151 MHz, $CDCl_3$) δ 197.0, 135.8, 131.9, 129.8, 128.3, 26.5.

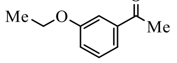

1-(3-ethoxyphenyl)ethan-1-one **2m**. (Toshikazu et al., 1991). Yield: 76% (M=164.20, 24.9 mg). ^{1}H NMR (600 MHz, $CDCl_3$) δ 7.54 – 7.50 (m, 1H), 7.48 (s, 1H), 7.36 (t, *J* = 7.9 Hz, 1H), 7.12 – 7.08 (m, 1H), 4.09 (q, *J* = 6.9 Hz, 2H), 2.59 (s, 3H), 1.43 (t, *J* = 7.0 Hz, 3H); ^{13}C NMR (151 MHz, $CDCl_3$) δ 198.0, 159.2, 138.5, 129.5, 121.0, 120.0, 113.1, 63.7, 26.7, 14.8.

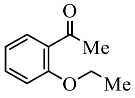

1-(2-ethoxyphenyl)ethan-1-one **2n**. (Guerrero and Rivero, 2008). Yield: 32% (M=164.20, 10.5 mg). ^{1}H NMR (600 MHz, $CDCl_3$) δ 7.74 (dd, *J* = 7.7, 1.7 Hz, 1H), 7.45 – 7.41 (m, 1H), 6.98 (t, *J* = 7.5 Hz, 1H), 6.94 (d, *J* = 8.3 Hz, 1H), 4.14 (q, *J* = 7.0 Hz, 2H), 2.64 (s, 3H), 1.48 (t, *J* = 7.0 Hz, 3H); ^{13}C NMR (151 MHz, $CDCl_3$) δ 200.0, 158.4, 133.6, 130.4, 128.4, 120.4, 112.3, 64.1, 32.01,14.8.

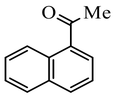

1-(naphthalen-1-yl)ethan-1-one **2o**. (Liao et al., 2020). Yield:53% (M=170.20, 18.0mg). ^{1}H NMR (600 MHz, $CDCl_3$) δ 8.75 (d, *J* = 8.6 Hz, 1H), 7.99 (d, *J* = 8.2 Hz, 1H), 7.93 (d, *J* = 7.2 Hz, 1H), 7.87 (d, *J* = 8.2 Hz, 1H), 7.62 – 7.58 (m, 1H), 7.55 – 7.51 (m, 1H), 7.51 – 7.48 (m, 1H), 2.74 (s, 3H); ^{13}C NMR (151 MHz, $CDCl_3$) δ 201.8, 135.5, 134.0, 133.0, 130.17, 128.7, 128.4, 128.1, 126.5, 126.0, 124.3, 30.0.

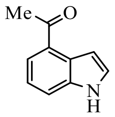

1-(1H-indol-4-yl)ethan-1-one **2p**. (He et al., 2008). Yield:60% (M=159.07, 19.0mg). ^{1}H NMR (600 MHz, $CDCl_3$) δ 8.80 (s, 1H), 8.35-8.32 (m, 1H), 7.88 (dd, *J* = 8.6, 1.6 Hz, 1H), 7.41 (d, *J* = 8.6 Hz, 1H), 7.31 – 7.27 (m, 1H), 6.68 – 6.65 (m, 1H), 2.68 (s, 3H); ^{13}C NMR (151 MHz, $CDCl_3$) δ 198.7, 138.6, 130.0, 127.5, 125.9, 123.1, 122.2, 111.1, 104.2, 26.7.

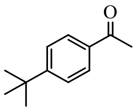

1-(4-(tert-butyl)phenyl)ethan-1-one **2q**. (Zhu et al., 2020). Yield: 55% (M= 176.25, 19.4 mg). ^{1}H NMR (600 MHz, $CDCl_3$) δ 7.90 (d, *J* = 8.3 Hz, 2H), 7.48 (d, *J* = 8.3 Hz, 2H), 2.58 (s, 3H), 1.34 (s, 9H); ^{13}C NMR (151 MHz, $CDCl_3$) δ 197.9, 156.8, 134.6, 128.3, 125.5, 35.1, 31.1, 26.6.

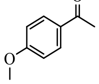

1-(4-methoxyphenyl)ethan-1-one **2r**. (Liao et al., 2020). Yield: 81% (M=150.18, 24.2 mg). ^{1}H NMR (600 MHz, $CDCl_3$) δ 7.94 (d, *J* = 8.4 Hz, 2H), 6.93 (d, *J* = 8.5 Hz, 2H), 3.87 (s, 3H), 2.55 (s, 3H); ^{13}C NMR (151 MHz, $CDCl_3$) δ 196.8, 163.5, 130.6, 130.4, 113.7, 55.5, 26.4.

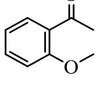

1-(2-methoxyphenyl)ethan-1-one **2s**. (Liao et al., 2020). Yield: 61% (M= 150.18, 18.3 mg). ^{1}H NMR (600 MHz, $CDCl_3$) δ 7.73 (dd, *J* = 7.7, 1.5 Hz, 1H), 7.48 – 7.44 (m, 1H), 7.00 (d, *J* = 7.5 Hz, 1H), 6.99

– 6.96 (m, 1H), 3.91 (s, 3H), 2.61 (s, 3H); ^{13}C NMR (151 MHz, $CDCl_3$) δ 199.9, 158.9, 133.7, 130.37, 128.3, 120.6, 111.6, 55.5, 31.8.

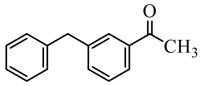

1-(3-benzylphenyl)ethan-1-one **2t**. (Denis et al., 2014). Yield: 56% (M= 210.10, 23.5 mg). Yield: 56% (M= 246, 23.5 mg). ^{1}H NMR (600 MHz, $CDCl_3$) δ 7.57 (s, 1H), 7.54 (d, *J* = 7.6 Hz, 1H), 7.44 (d, *J* = 7.4 Hz, 2H), 7.39 (t, *J* = 6.9 Hz, 2H), 7.36 (d, *J* = 7.8 Hz, 1H), 7.33 (t, *J* = 7.3 Hz, 1H), 7.17 (dd, *J* = 8.2, 2.5 Hz, 1H), 5.10 (s, 2H), 2.58 (s, 3H); ^{13}C NMR (151 MHz, $CDCl_3$) δ 197.9, 159.0, 138.5, 136.5, 129.7, 128.7, 128.2, 127.6, 121.4, 120.3, 113.6, 70.2, 26.8.

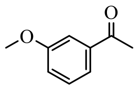

1-(3-methoxyphenyl)ethan-1-one **2u**. (Zhu et al., 2020). Yield:70%(M=150.18, 21.1 mg). ^{1}H NMR (600 MHz, $CDCl_3$) δ 7.54 (d, *J* = 7.6 Hz, 1H), 7.50 – 7.48 (m, 1H), 7.37 (t, *J* = 7.9 Hz, 1H), 7.13 – 7.10 (m, 1H), 3.86 (s, 3H), 2.60 (s, 3H); ^{13}C NMR (151 MHz, $CDCl_3$) δ 197.9, 159.8, 138.5, 129.6, 121.1, 119.6, 112.4, 55.4, 26.7.

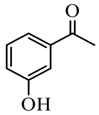

1-(3-hydroxyphenyl)ethan-1-one **2v**. (Song et al., 2020). Yield: 79% (M= 136.15, 21.4 mg). ^{1}H NMR (600 MHz, $CDCl_3$) δ 7.52 (d, *J* = 7.4 Hz, 1H), 7.46 (s, 1H), 7.35 (t, *J* = 7.9 Hz, 1H), 7.07 (dd, *J* = 8.1, 2.5 Hz, 1H), 5.37 (s, 1H), 2.59 (s, 3H); ^{13}C NMR (151 MHz, $CDCl_3$) δ 198.3, 156.1, 138.6, 129.9, 121.1, 120.5, 114.7, 26.8.

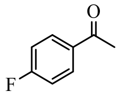

1-(4-fluorophenyl)ethan-1-one **2w**. (Zhu et al., 2020). Yield: 37% (M= 138.1,10.1 mg). ^{1}H NMR (600 MHz, $CDCl_3$) δ 7.99 (dd, *J* = 8.3, 5.6 Hz, 2H), 7.13 (t, *J* = 8.5 Hz, 2H), 2.59 (s, 3H); ^{13}C NMR (151 MHz, $CDCl_3$) δ 196.5, 165.8 (d, *J* = 254.6 Hz, 1C), 133.6 (d, *J* = 2.9 Hz, 1C), 131.0 (d, *J* = 9.3 Hz, 1C), 115.7 (d, *J* = 21.9 Hz, 1C), 26.6.

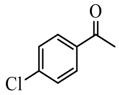

1-(4-chlorophenyl)ethan-1-one **2x**. (Liao et al., 2020). Yield:39% (M= 154.59, 12.0mg). ^{1}H NMR (600 MHz, $CDCl_3$) δ 7.90 (d, *J* = 8.5 Hz, 2H), 7.44 (d, *J* = 8.5 Hz, 2H), 2.59 (s, 3H); ^{13}C NMR (151 MHz, $CDCl_3$) δ 196.8, 139.6, 135.5, 129.7, 128.9, 26.6.

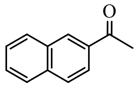

1-(naphthalen-2-yl)ethan-1-one **2y**. (Liao et al., 2020).Yield: 37% (M= 170.2, 12.6 mg). ^{1}H NMR (600 MHz, $CDCl_3$) δ 8.45 (s, 1H), 8.03 (dd, *J* = 8.6, 1.7 Hz, 1H), 7.95 (d, *J* = 8.1 Hz, 1H), 7.87 (t, *J* = 8.5 Hz, 2H), 7.61 – 7.57 (m, 1H), 7.57 – 7.52 (m, 1H), 2.72 (s, 3H); ^{13}C NMR (151 MHz, $CDCl_3$) δ 198.1, 135.6, 134.5, 132.6, 130.2, 129.6, 128.5, 128.4, 127.8, 126.8, 123.9, 26.7.

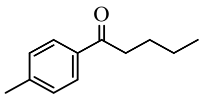

1-(p-tolyl)pentan-1-one **2A**. (Hu et al., 2018).Yield: 42% (M= 176.26, 15.0mg). ^{1}H NMR (600 MHz, $CDCl_3$) δ 7.86 (d, *J* = 8.1 Hz, 2H), 7.25 (d, *J* = 8.6 Hz, 2H), 2.94 (t, *J* = 7.5 Hz, 2H), 2.41 (s, 3H), 1.74 – 1.69 (m, 2H), 1.44 – 1.37 (m, 2H), 0.95 (t, *J* = 7.4 Hz, 3H); ^{13}C NMR (151 MHz, $CDCl_3$) δ 200.3, 143.6, 134.7, 129.2, 128.2, 38.3, 26.7, 22.5, 21.6, 14.0.

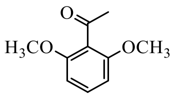

1-(2,6-dimethoxyphenyl)ethan-1-one **2B**. (Lindh et al., 2010). Yield: 94% (M= 180.00, 33.8 mg). ^{1}H NMR (600 MHz, $CDCl_3$) δ 7.27 – 7.24 (m, 1H), 6.55 (d, *J* = 8.4 Hz, 2H), 3.80 (s, 6H), 2.48 (s, 3H);^{13}C NMR (151 MHz, $CDCl_3$) δ 202.8, 156.7, 130.6, 120.6, 104.0, 55.9, 32.3.

References:

Li, F., Tang, S., Tang, Z. L., Ye, L. J., Li, H. H., Niu, F. F., et al. (2020). Synergistic Catalytic Effect of *N*-Hydroxyphthalimide/Cobalt Tetraamide Phthalocyanine and Its Application for Aerobic Oxidation of Hydrocarbons and Alcohols. *Catal Letters.* 151(1), 17-26. doi: 10.1007/s10562-020-03283-z.

Wang, X. Y., Liu, M. C., Xu, L., Wang, Q. Z., Chen, J. X., Ding. J. C., et al. (2013). Palladium-Catalyzed Addition of Potassium Aryltrifluoroborates to Aliphatic Nitriles: Synthesis of Alkyl Aryl Ketones, Diketone Compounds, and 2-Arylbenzo[b]furans. *J. Org. Chem.* 78(11), 5273-5281. doi: 10.1021/jo400433m.

Hu, W. K., Shan, L., Ma, F. D., Zhang, Y. L.,Yang, Y. C., and Wang, D. W. (2019). The synthesis of methyl triazole-4-carboxylate gold(I) complex and application on allene synthesis and alkyne hydration. *Inorg Chem Commun. 109*, 107564. doi:10.1016/j.inoche.2019.107564.

Suta, K., and Turks, M. (2018). In(III) and Hf(IV) Triflate-Catalyzed Hydration and Catalyst-free Hydrohalogenation of Aryl Acetylenes in Liquid Sulfur Dioxide. *ACS Omega.* 12(3), 18065-18077. doi: 10.1021/acsomega.8b01630.

Sun, Y. N., Wang, E. D., Ren, Y. J., Xiao, K., Liu, X., Yang, D., et al. (2019). The Evolution in Catalytic Activity Driven by Periodic Transformation in the Inner Sites of Gold Clusters. *Adv. Funct. Mater.* 29(38), 1904242-1904249. doi: 10.1002/adfm.201904242.

Thiruvengetam, P., Chakravarthy, R., and Chand, D. (2019). A molybdenum based metallomicellar catalyst for controlled and chemoselective oxidation of activated alcohols in aqueous medium. *J. Catal. 376*, 123-133. doi:10.1016/j.jcat.2019.06.013.

Qin, Y. Z., Martindale, B. M., Rieth, A., and Nocera, D. (2020). Solar-driven tandem photoredox nickel-catalysed cross-coupling using modified carbon nitride *Chem. Sci.* 11(28), 7456-7461. doi:10.1039/D0SC02131H.

Bhatt, R., Sharma, A., Himanshi., Bhuvanesh, N., and Joshi, H. (2020). Palladium complexes of chalcogenoethanamine (S/Se) bidentate ligands: Applications in catalytic arylation of C-H and O-H bonds. *Polyhedron*. 185, 114597. doi:10.1016/j.poly.2020.114597.

Xie, P., Xue, C., Luo, J. F., Shi, S. S., and Du, D. D. (2021). Decatungstate-mediated solar photooxidative cleavage of CvC bonds using air as an oxidant in water. *Green Chem.* 23(16), 5936-5943. doi: 10.1039/d1gc01234g.

Wang, J.-T., Peng, J.-G., Zhang, J.-Q., Wang, Z.-X., Zhang, Y., Zhou, X.-R., et al. (2019). Novel berberine-based derivatives with potent hypoglycemic activity. *Bioorg Med Chem Lett.* 29(23), 126709. doi: 10.1016/j.bmcl.2019.126709.

Hao, Z. Q., Liu, K., Feng, Q., Dong, Q., Ma, D. Z., Han, Z. G., et al. (2019). Ruthenium(II) complexes bearing Schiff base ligands for the efficient acceptorless dehydrogenation of secondary alcohols. *Chin. J. Chem.* 39(1), 121-128. doi: 10.1002/cjoc.202000363.

Toshikazu, H., Satoshi, M., Makoto,M., and Yoshiki, O. (1991). NOVEL ALLYLW OXIDATION OF CONJUGATE KETONES WITH VO(OR)C12. *Teirahcdron letters.* 32(14), 1741-1744. doi:10.1016/S0040-4039(00)74318-8.

Guerrero, L., and Rivero, I. (2008). 1,2-Dimethylimidazole (DMI) and microwaves in the alkylation of carboxylic acids and phenols with dimethyl and diethyl carbonates. *ARKIVOC.* (11), 295-306. doi:10.3998/ark.5550190.0009.b29.

Liao, S. F., Liu, J.G., Yan, L., Liu, Q. Y., Chen, G. H., and Ma, L. L. (2020). 2-Bromoanthraquinone as a highly efficient photocatalyst for the oxidation of sec-aromatic alcohols: experimental and DFT study *RSC Adv. 10(61)*, 37014-37022. doi: 10.1039/d0ra06414a.

He, T. X., Tao, X. C., Wu, X. Y., Cai, L. S., and Pike, V. (2008). Acetylation of *N*-Heteroaryl Bromides via PdCl2/(*o*-tolyl)3P Catalyzed Heck Reactions. *Synthesis. (6)*, 887-890. doi:10.1055/s-2008-1032193.

Zhu, X. J., Liu, C., Liu, Y., Yang, H. J., and Fu, H. (2020). A sodium trifluoromethanesulfinate-mediated photocatalytic strategy for aerobic oxidation of alcohols. *Chem Commun.* 56(82), 12443-12446. doi:10.1039/D0CC05799A.

Liao, S. F., Liu, J.G., Yan, L., Liu, Q. Y., Chen, G. H., and Ma, L. L. (2020). 2-Bromoanthraquinone as a highly efficient photocatalyst for the oxidation of sec-aromatic alcohols: experimental and DFT study. *RSC Adv*. 10(61), 37014-37022. doi: 10.1039/d0ra06414a.

Liao, S. F., Liu, J.G., Yan, L., Liu, Q. Y., Chen, G. H., and Ma, L. L. (2020). 2-Bromoanthraquinone as a highly efficient photocatalyst for the oxidation of sec-aromatic alcohols: experimental and DFT study. *RSC Adv*. 10(61), 37014-37022. doi: 10.1039/d0ra06414a.

Denis, J. S., Scully, C. G., Lee, C. F., and Yudin, A. k. (2014). Development of the Direct Suzuki−Miyaura Cross-Coupling of Primary B-Alkyl MIDA-boronates and Aryl Bromides. *Org. Lett.* 16(5), 6017-6116. doi:10.1021/ol500057a.

Zhu, X. J., Liu, C., Liu, Y., Yang, H. J., and Fu, H. (2020). A sodium trifluoromethanesulfinate-mediated photocatalytic strategy for aerobic oxidation of alcohols. *Chem Commun.* 56(82), 12443-12446. doi:10.1039/D0CC05799A.

Song, Z.-Q., Wang, D.-H. (2020). Palladium-Catalyzed Hydroxylation of Aryl Halides with Boric Acid. *Org Lett.* 22(21), 8470-8474. doi:10.1021/acs.orglett.0c03069.

Zhu, X. J., Liu, C., Liu, Y., Yang, H. J., and Fu, H. (2020). A sodium trifluoromethanesulfinate-mediated photocatalytic strategy for aerobic oxidation of alcohols. *Chem Commun.* 56(82), 12443-12446. doi:10.1039/D0CC05799A.

Liao, S. F., Liu, J.G., Yan, L., Liu, Q. Y., Chen, G. H., and Ma, L. L. (2020). 2-Bromoanthraquinone as a highly efficient photocatalyst for the oxidation of sec-aromatic alcohols: experimental and DFT study. *RSC Adv*. 10(61), 37014-37022. doi: 10.1039/d0ra06414a.

Liao, S. F., Liu, J.G., Yan, L., Liu, Q. Y., Chen, G. H., and Ma, L. L. (2020). 2-Bromoanthraquinone as a highly efficient photocatalyst for the oxidation of sec-aromatic alcohols: experimental and DFT study. *RSC Adv*. 10(61), 37014-37022. doi: 10.1039/d0ra06414a.

Hu, Y. L, Wu, Y. P., and Lu, M. (2018). Co (II)-C12 alkyl carbon chain multi-functional ionic liquid immobilized on nano-SiO2 nano-SiO2@CoCl3-C12IL as an efficient cooperative catalyst for C–H activation by direct acylation of aryl halides with aldehydes. *Appl Organometal Chem.* 32(2), 4096-4103. doi: 10.1002/aoc.4096.

Lindh, J., Sjoberg, P. R., and Larhed, M. (2010). Synthesis of Aryl Ketones by Palladium(II)-Catalyzed Decarboxylative Addition of Benzoic Acids to Nitriles. *Angew. Chem. In.t Ed.* 49(42), 7733-7737. doi:10.1002/anie.201103009.

6 ^{1}H NMR and ^{13}C NMR Spectra of the Products

^{1}H NMR of 1-(p-tolyl)ethan-1-one **2a**

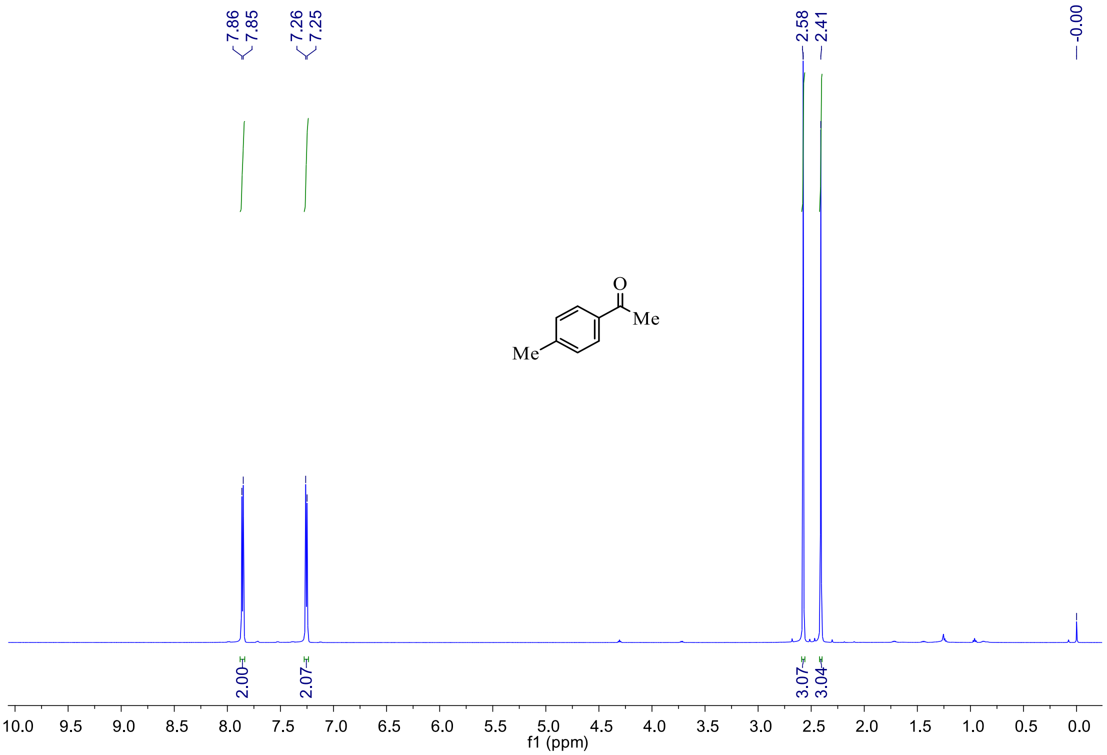

^{13}C NMR of 1-(p-tolyl)ethan-1-one **2a**

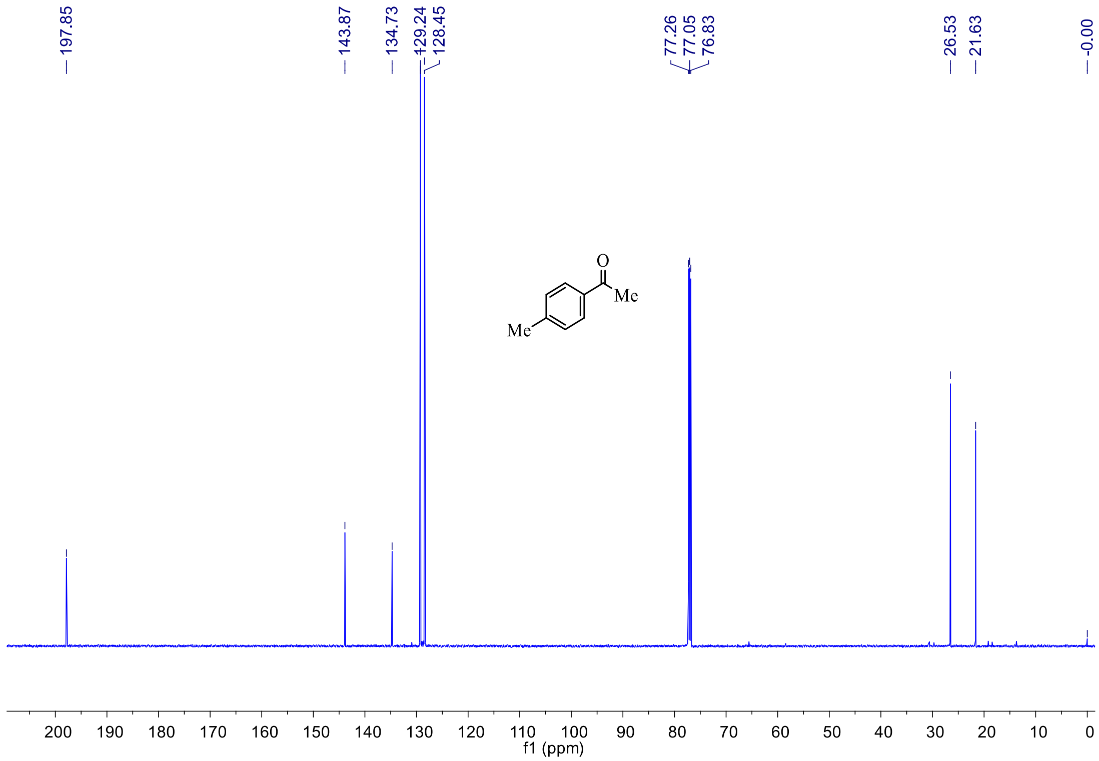

^{1}H NMR of Acetophenone **2b**

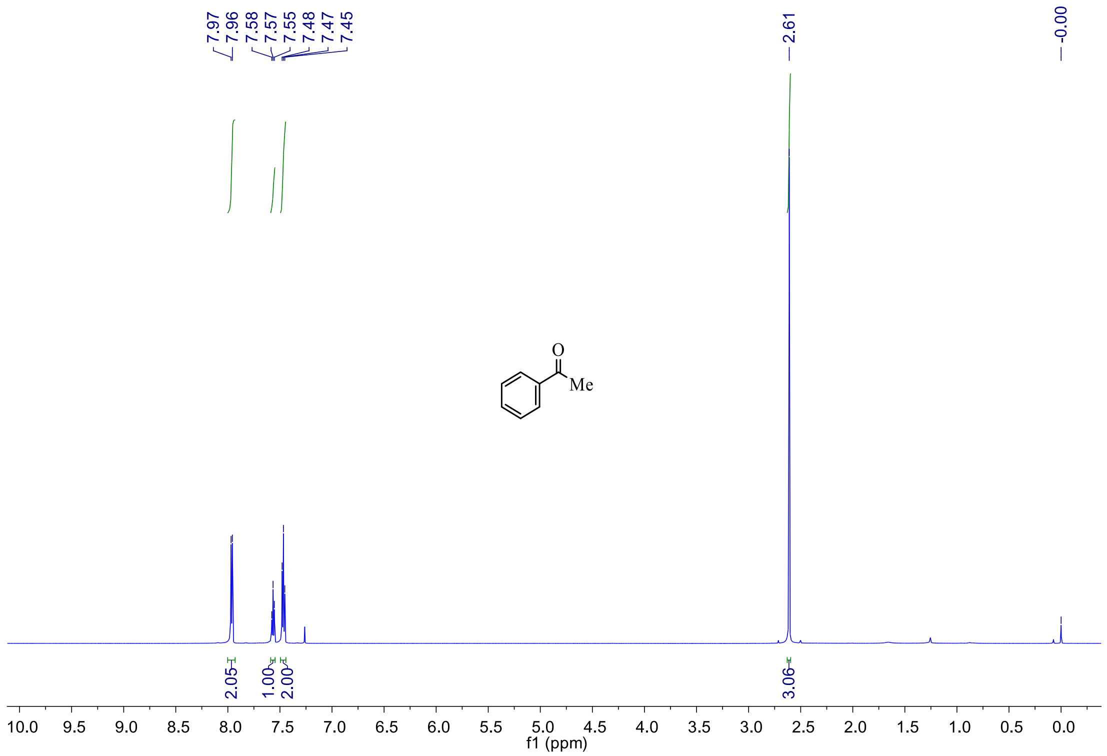

^{13}C NMR of Acetophenone **2b**

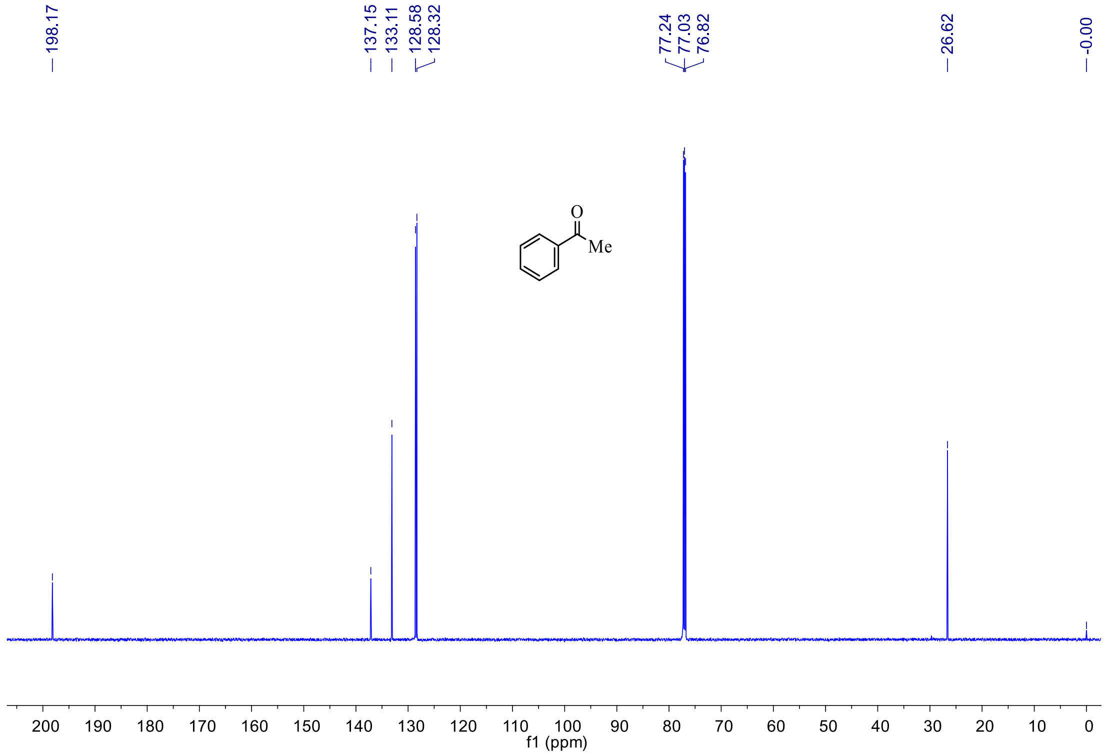

^{1}H NMR of 1-(4-ethylphenyl)ethan-1-one **2c**

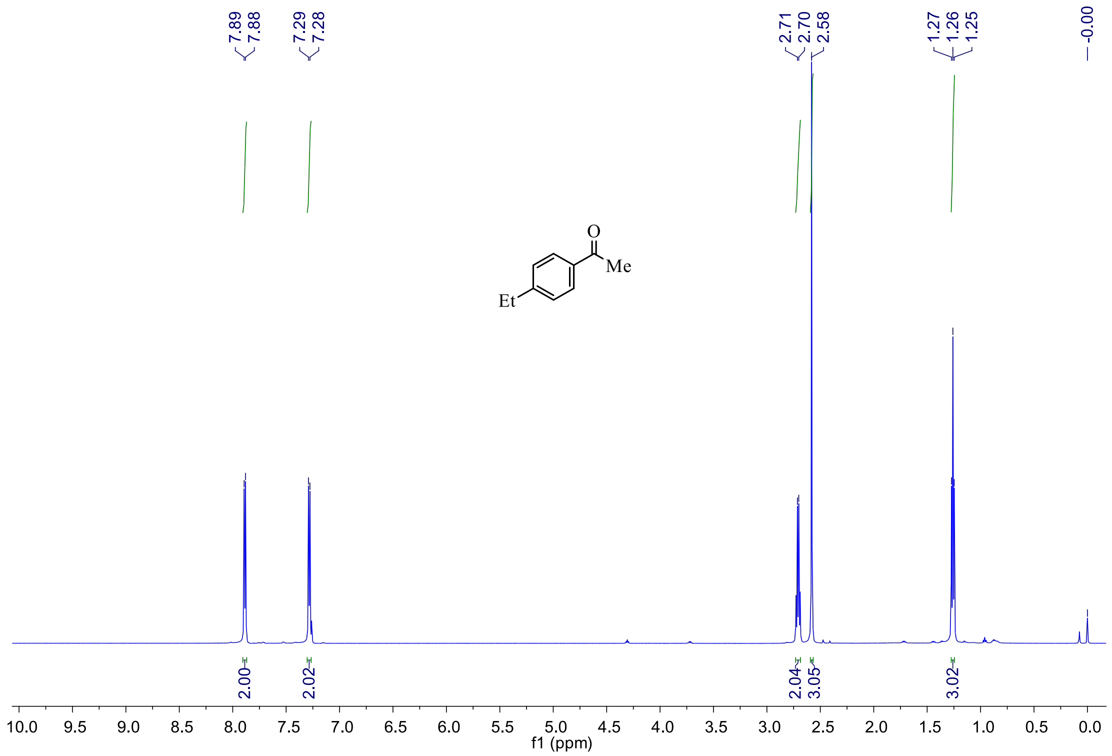

^{13}C NMR of 1-(4-ethylphenyl)ethan-1-one **2c**

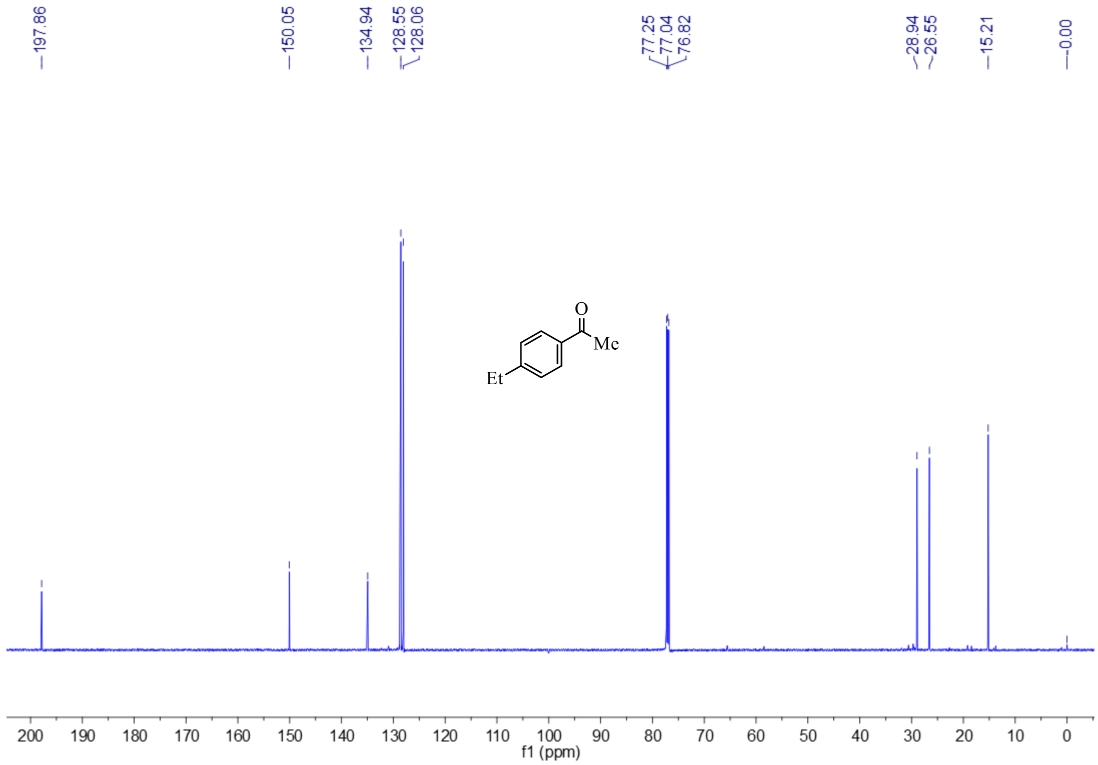

^{1}H NMR of 1-(4-propylphenyl)ethan-1-one **2d**

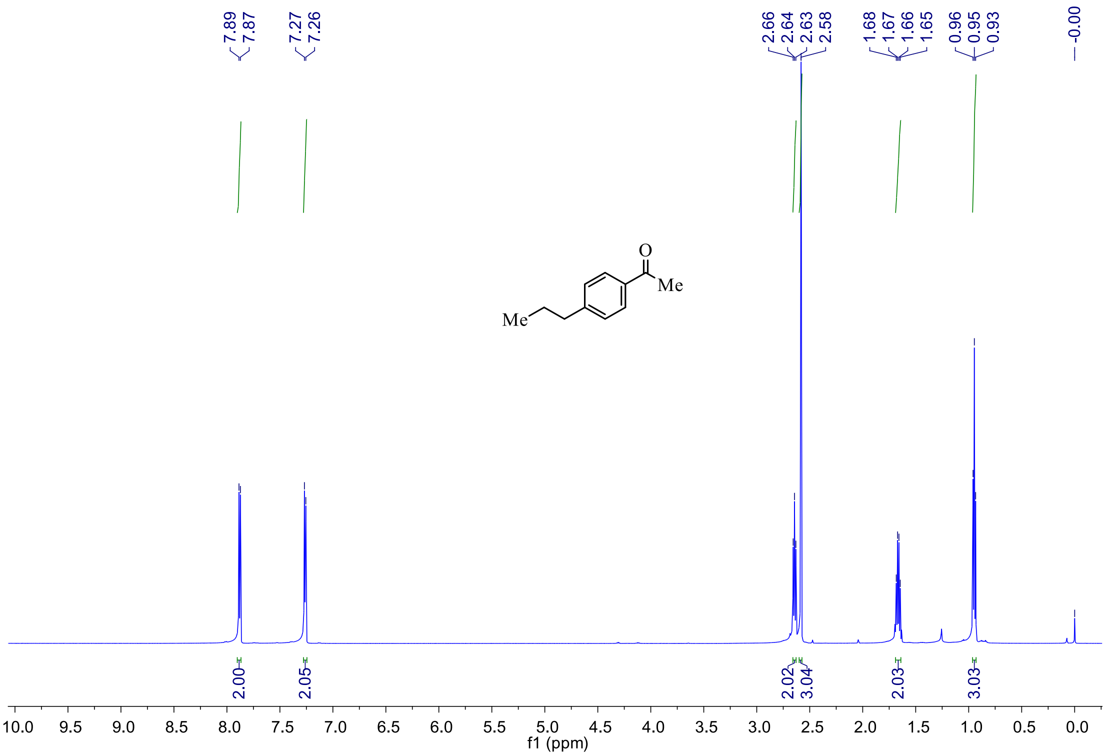

^{13}C NMR of 1-(4-propylphenyl)ethan-1-one **2d**

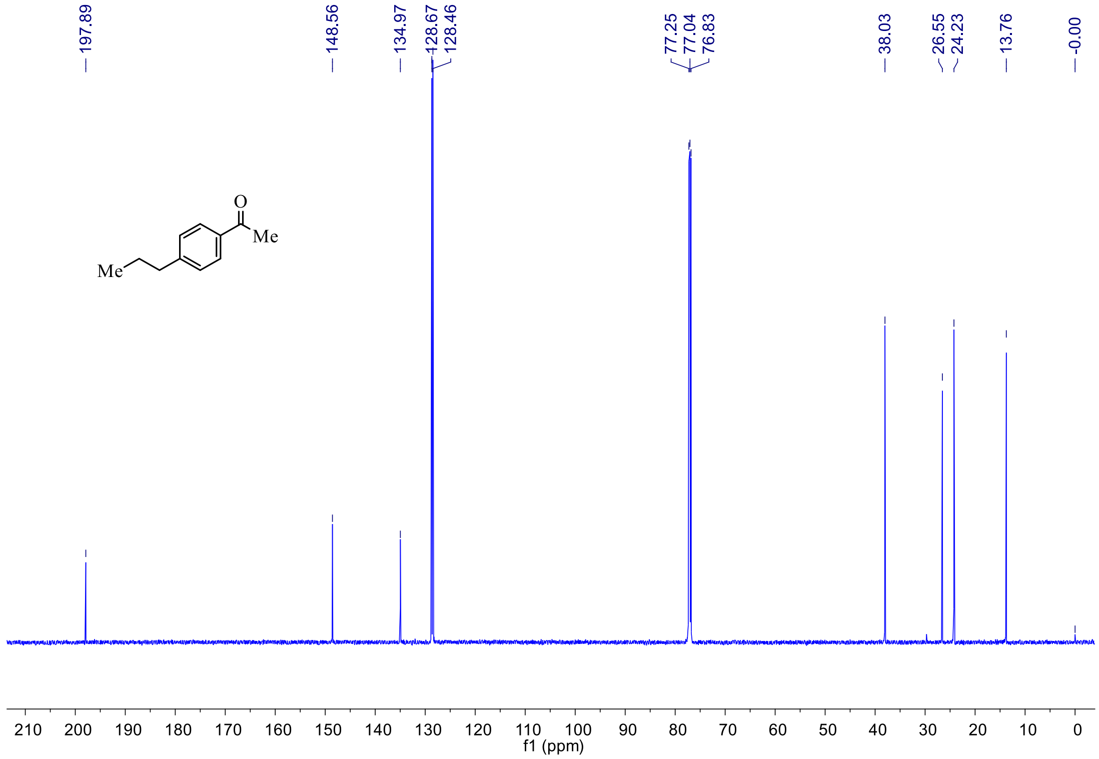

^{1}H NMR of 1-(4-pentylphenyl)ethan-1-one **2e**

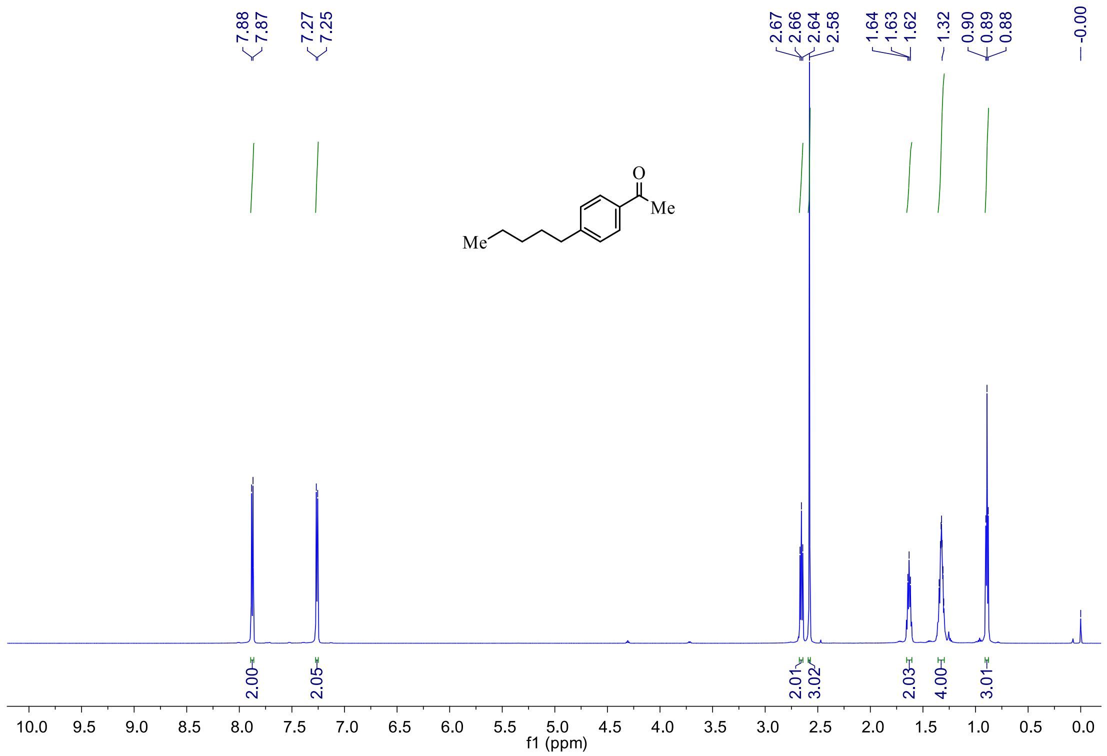

^{13}C NMR of 1-(4-pentylphenyl)ethan-1-one **2e**

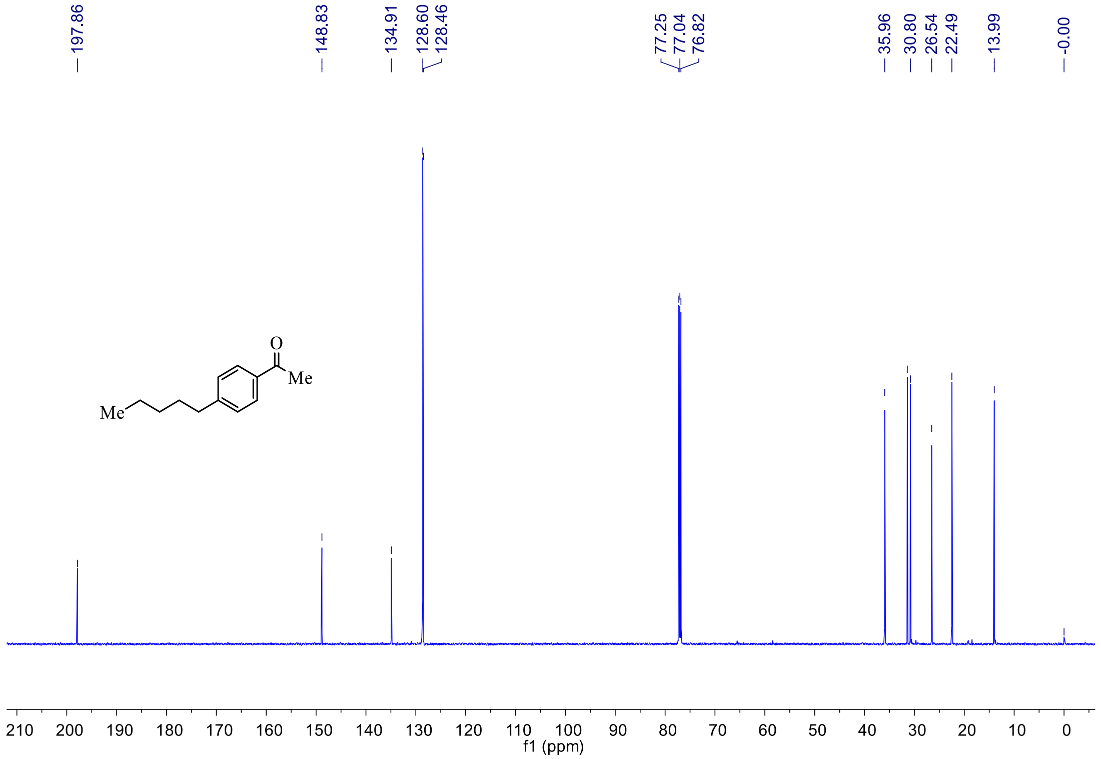

^{1}H NMR of 1-(4-ethoxyphenyl)ethan-1-one **2f**

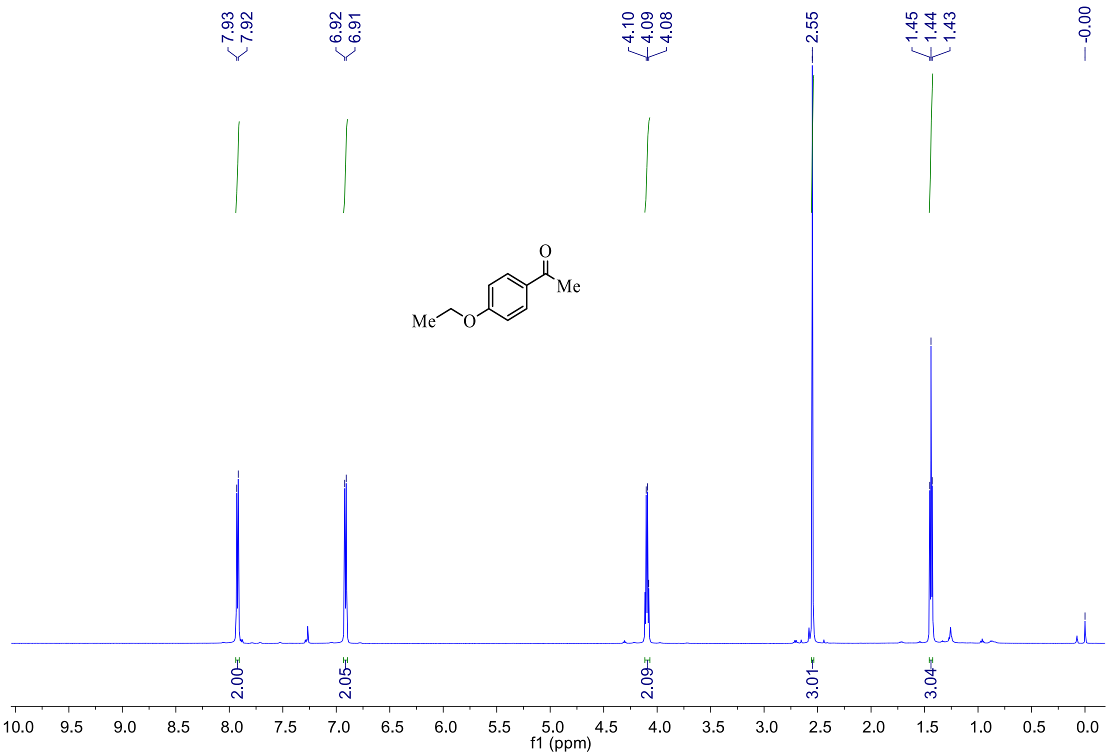

^{13}C NMR of 1-(4-ethoxyphenyl)ethan-1-one **2f**

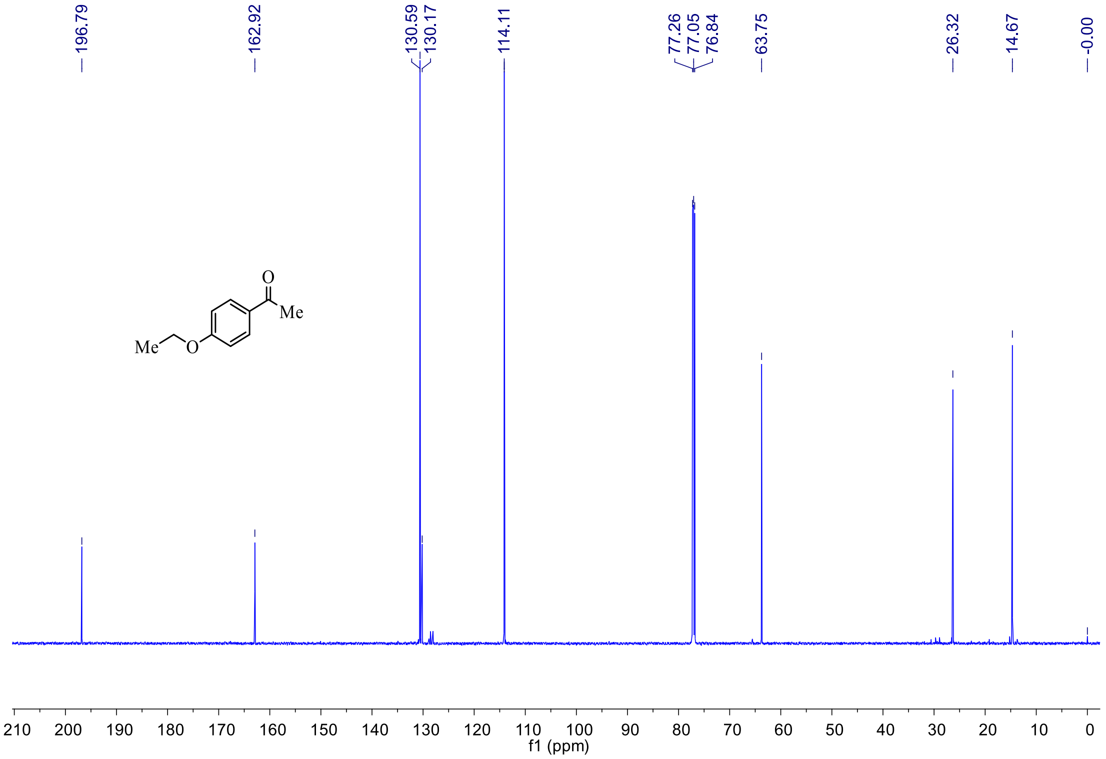

^{1}H NMR of 1-(4-isopropoxyphenyl)ethan-1-one **2g**

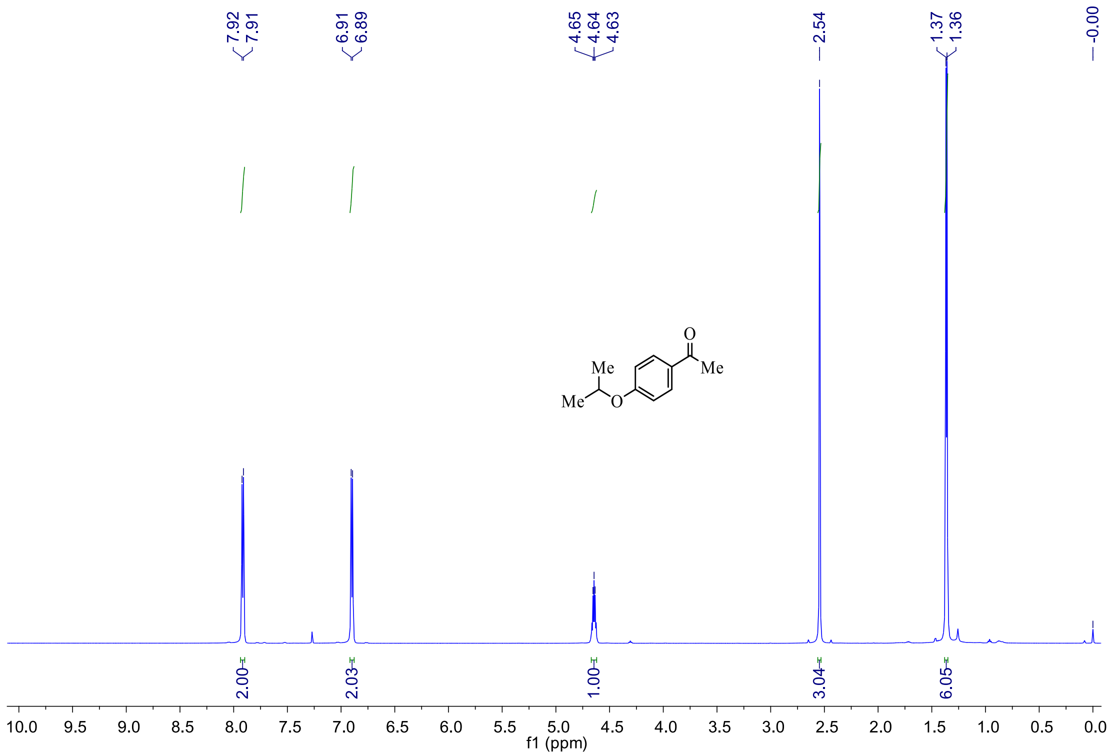

^{13}C NMR of 1-(4-isopropoxyphenyl)ethan-1-one **2g**

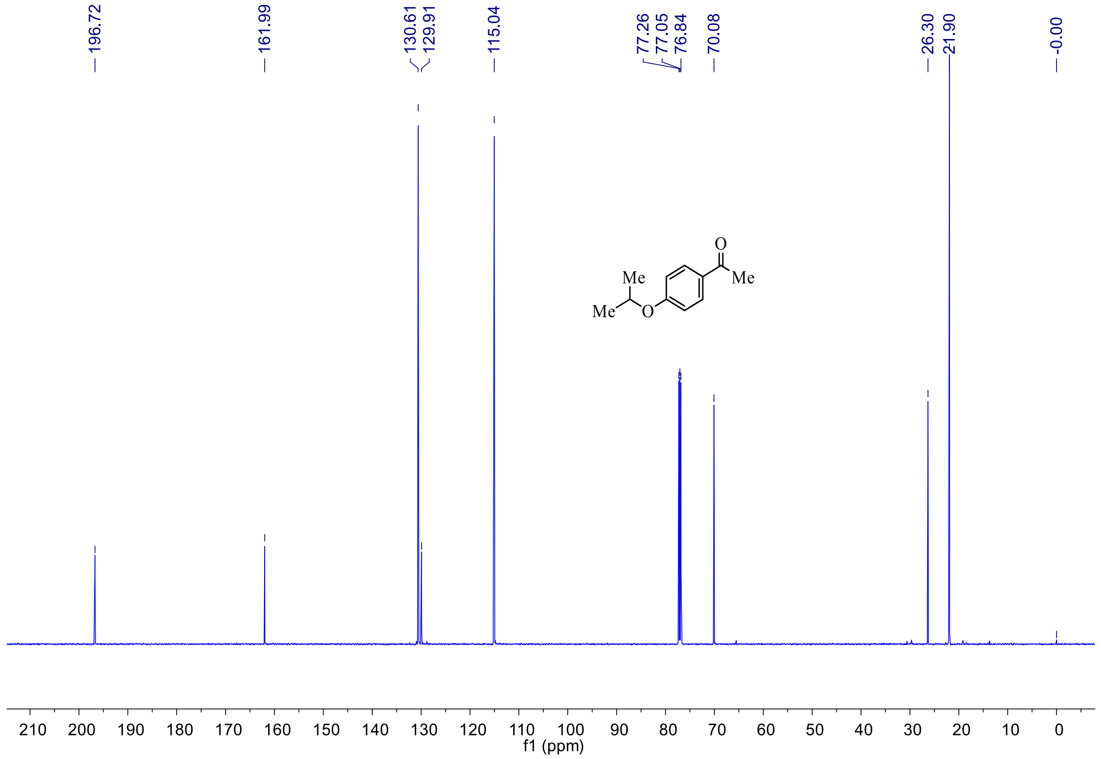

^{1}H NMR of1-(4-phenoxyphenyl)ethan-1-one **2h**

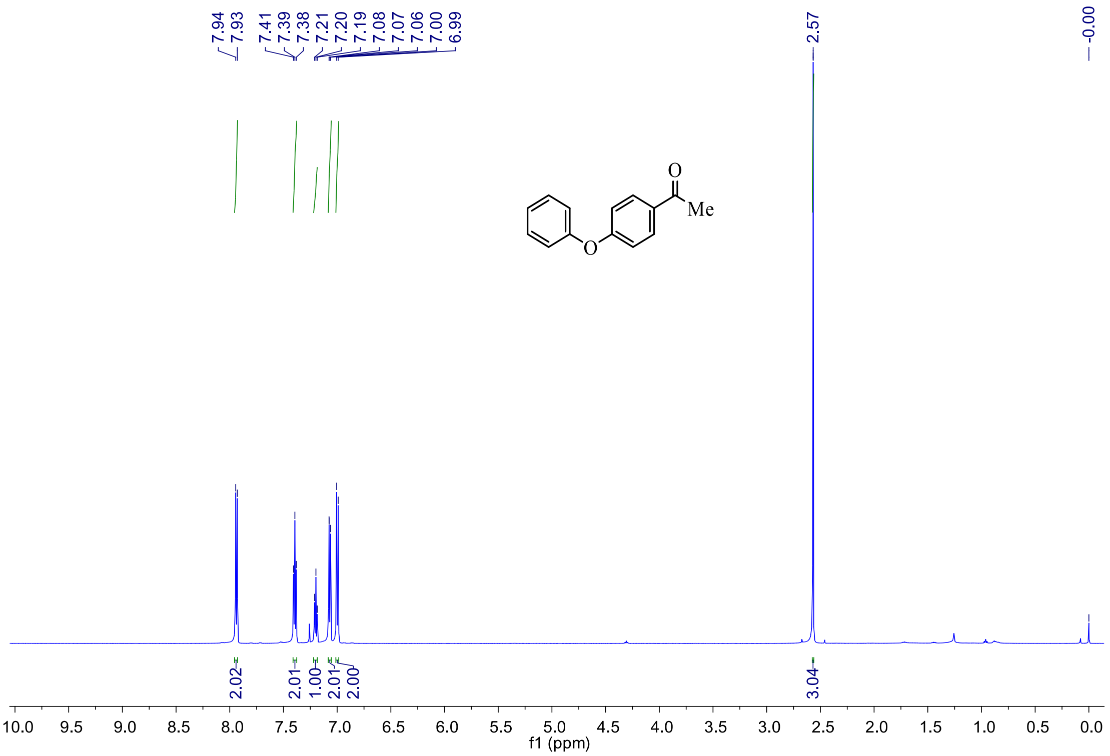

^{13}C NMR of1-(4-phenoxyphenyl)ethan-1-one **2h**

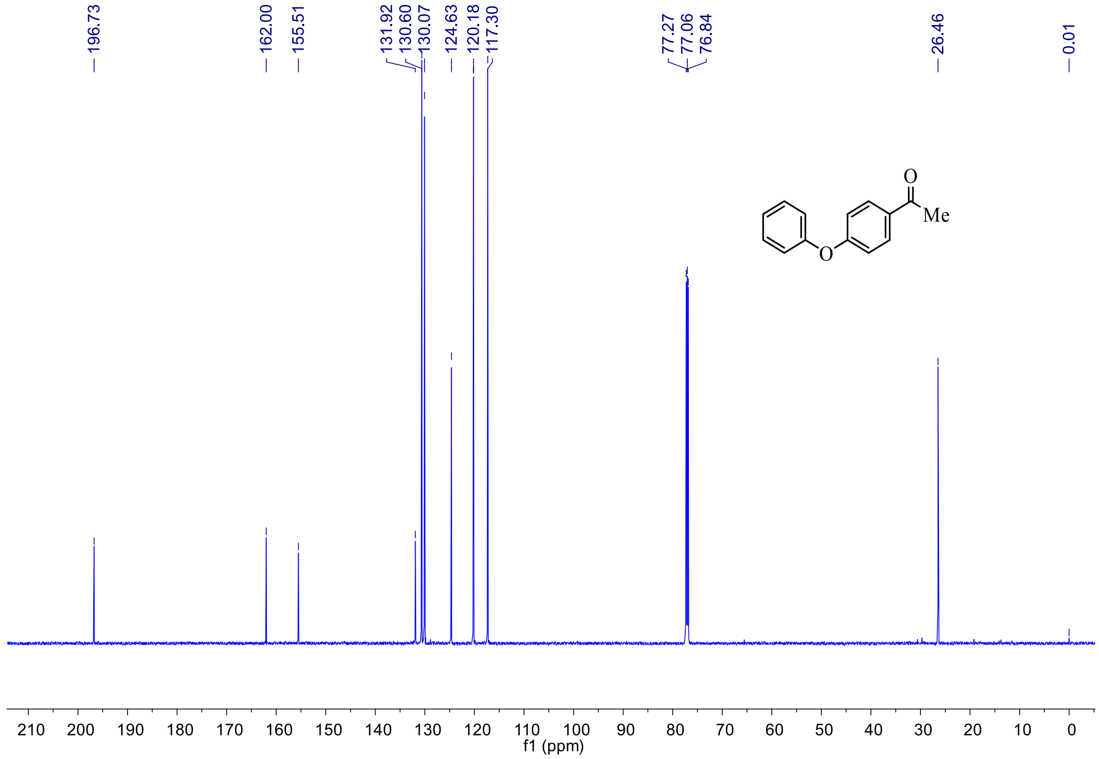

^{1}H NMR of 1-(4-(trifluoromethyl)phenyl)ethan-1-one **2i**

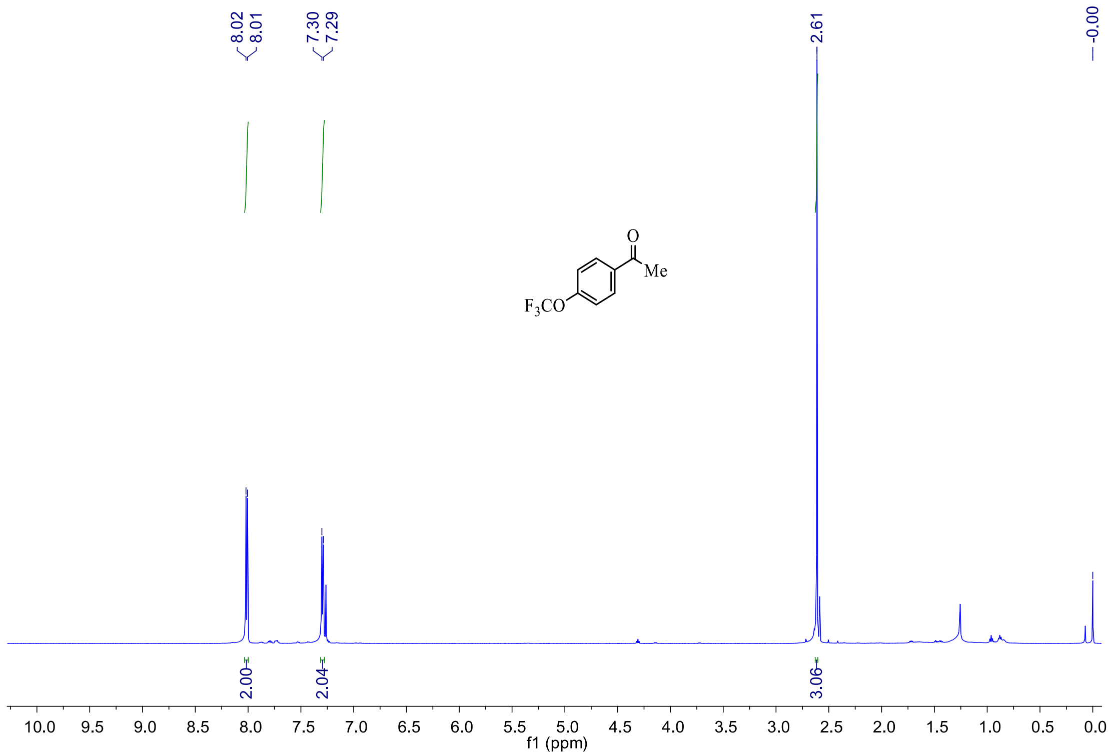

^{13}C NMR of 1-(4-(trifluoromethyl)phenyl)ethan-1-one **2i**

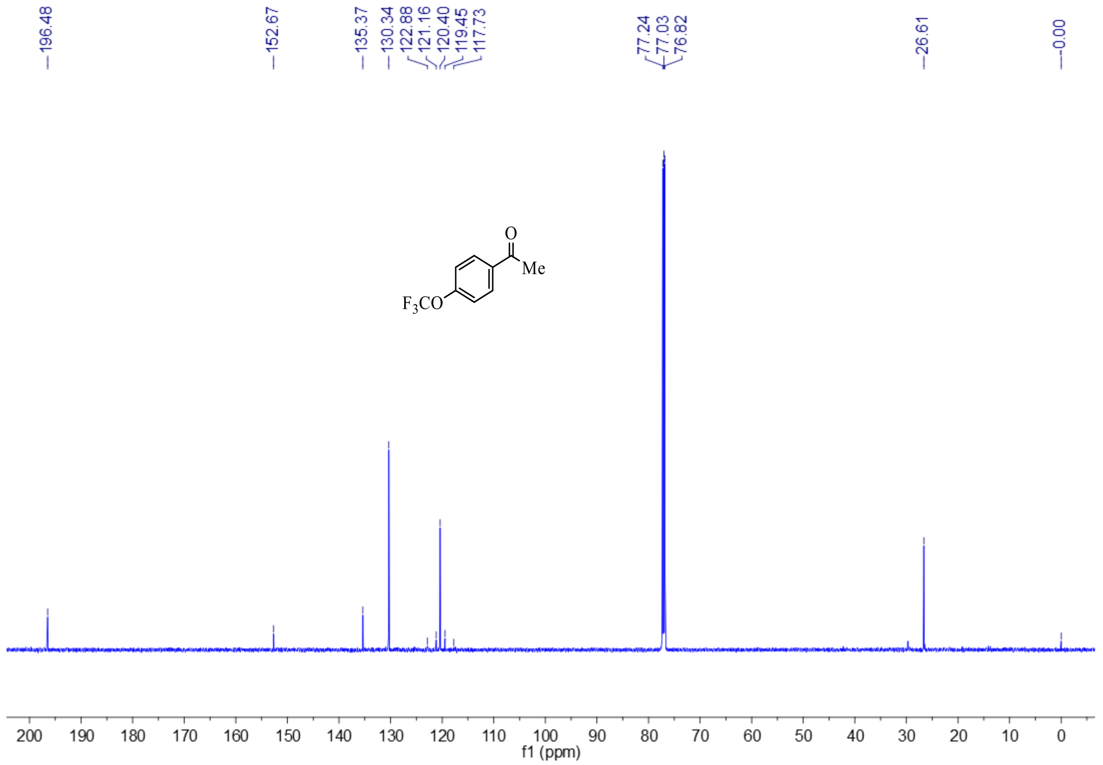

^{1}H NMR of1-(benzo[d][1,3]dioxol-5-yl)ethan-1-one **2j**

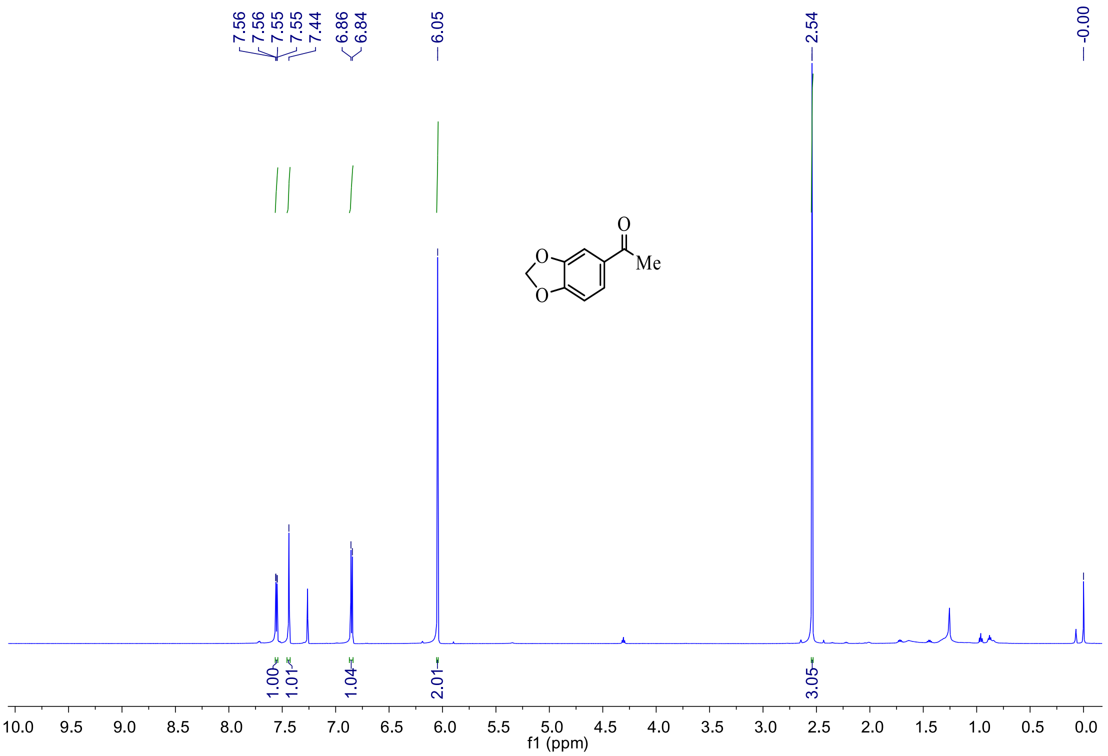

^{13}C NMR of 1-(benzo[d][1,3]dioxol-5-yl)ethan-1-one **2j**

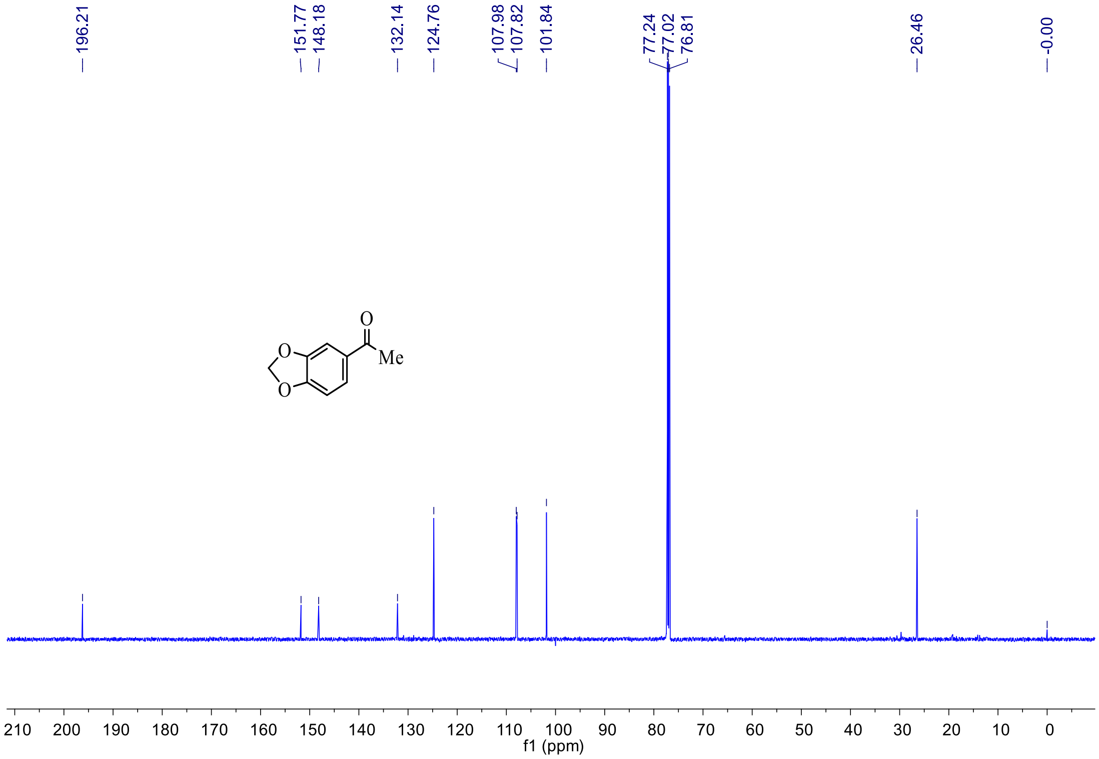

^{1}H NMR of 1-(4-bromophenyl)ethan-1-one **2k**

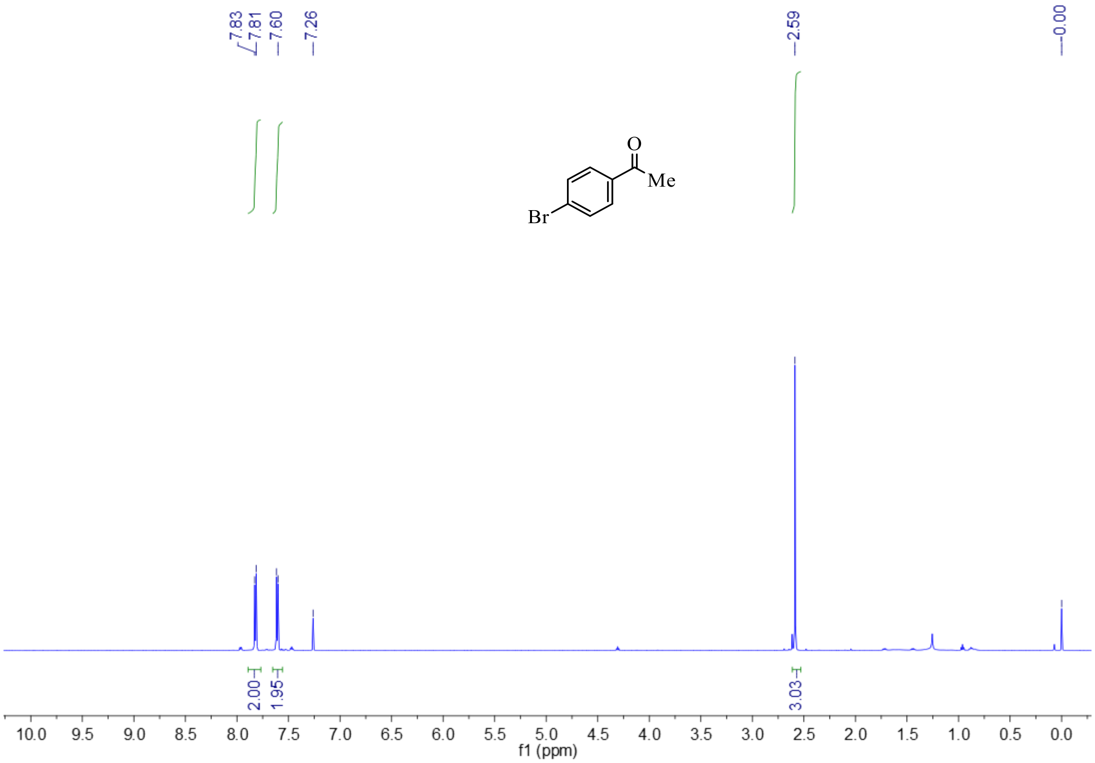

^{13}C NMR of 1-(4-bromophenyl)ethan-1-one **2k**

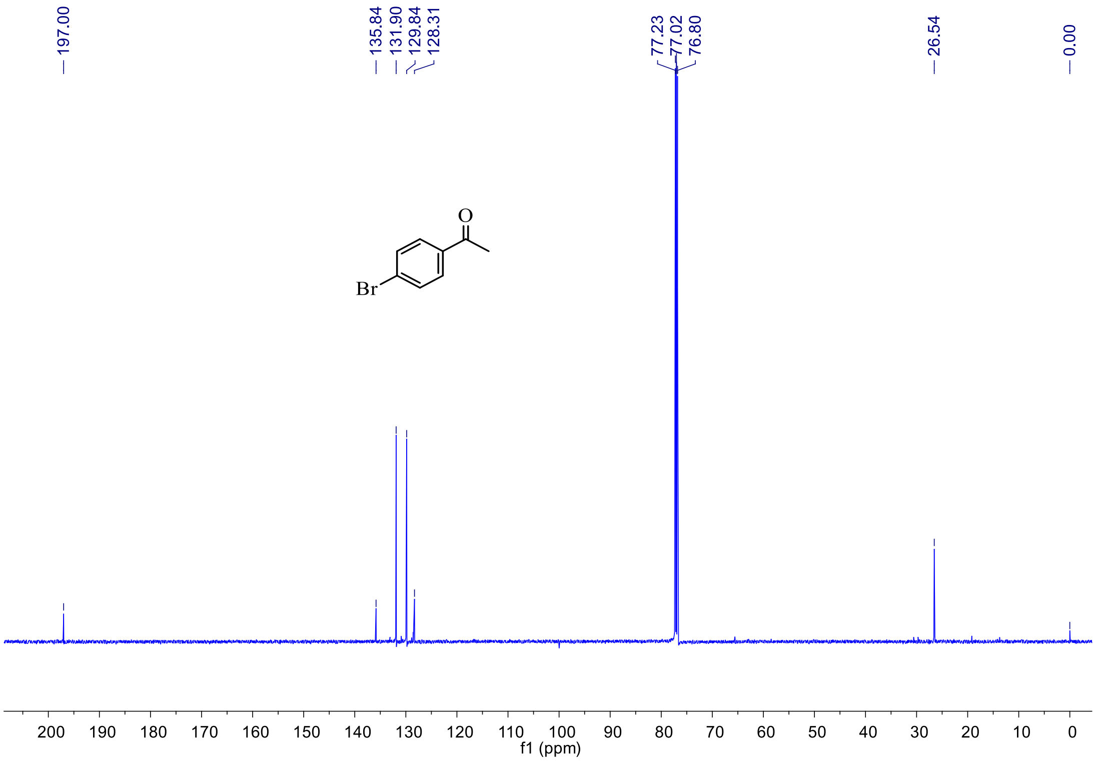

^{1}H NMR of 1-(3-ethoxyphenyl)ethan-1-one **2m**

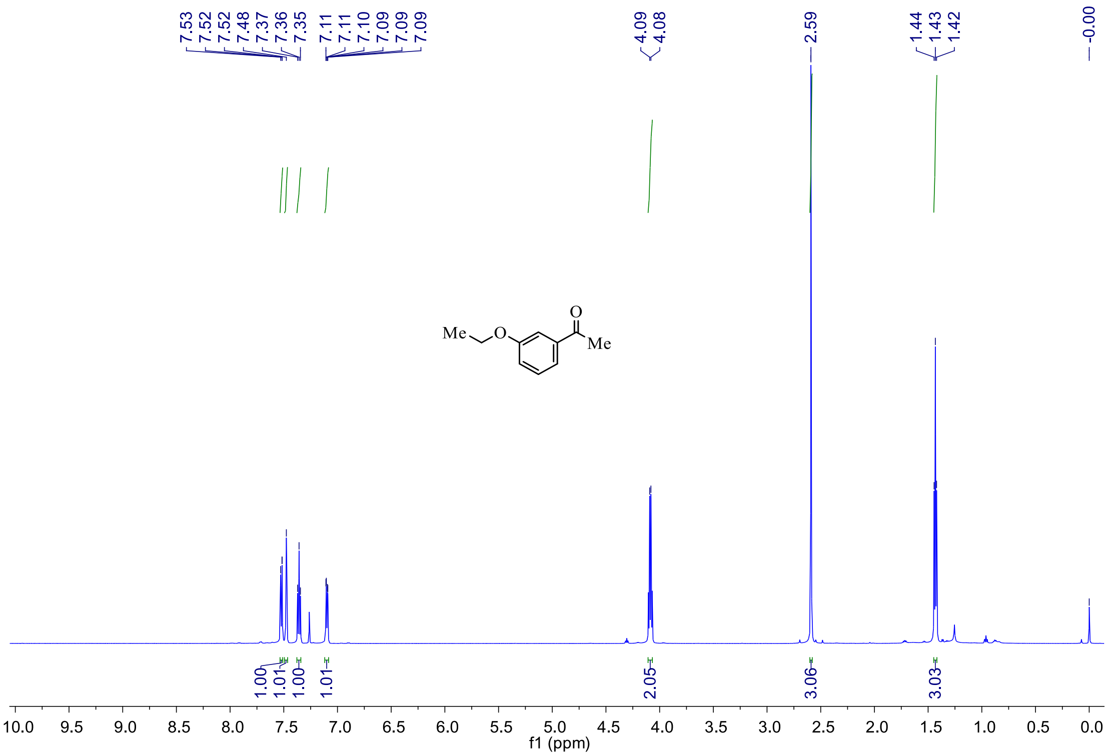

^{13}C NMR of 1-(3-ethoxyphenyl)ethan-1-one **2m**

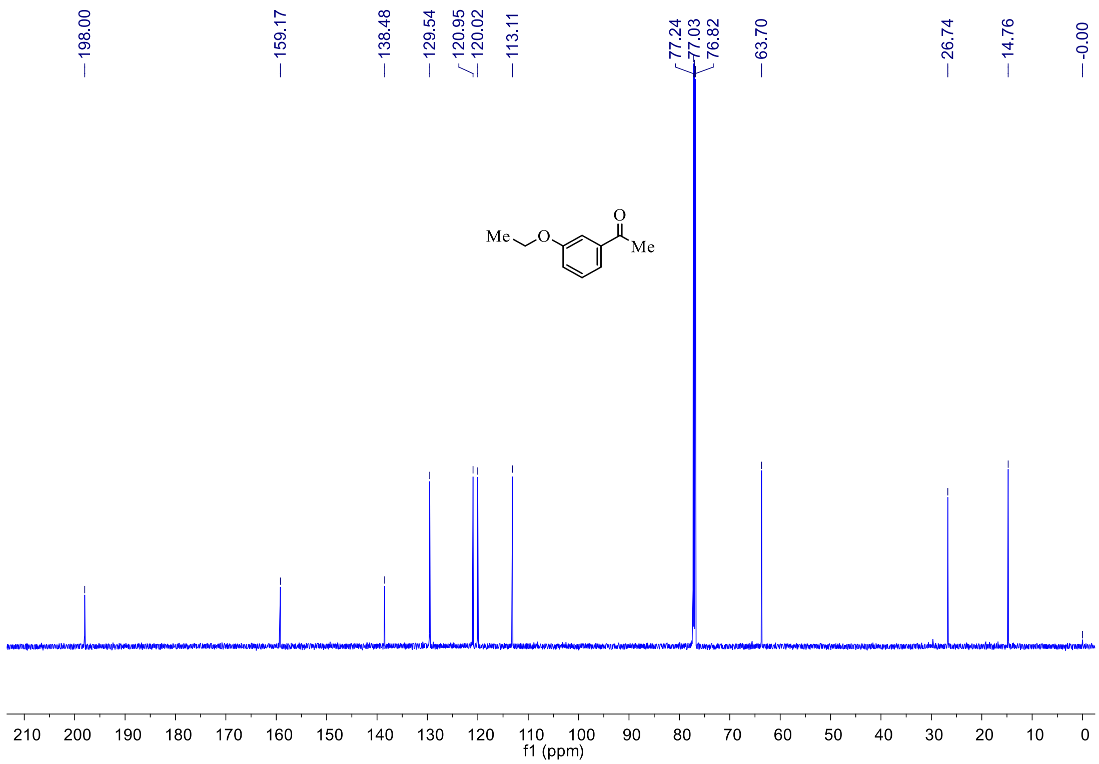

^1H NMR of 1-(2-ethoxyphenyl)ethan-1-one **2n**

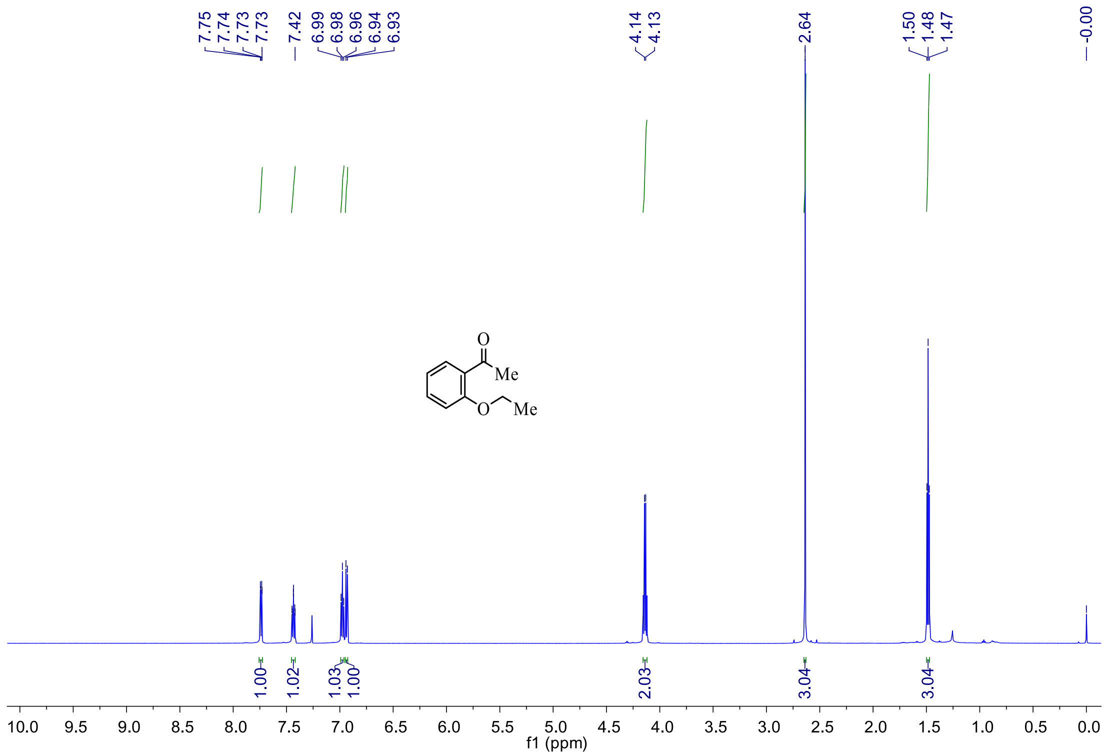

^{13}C NMR of 1-(2-ethoxyphenyl)ethan-1-one **2n**

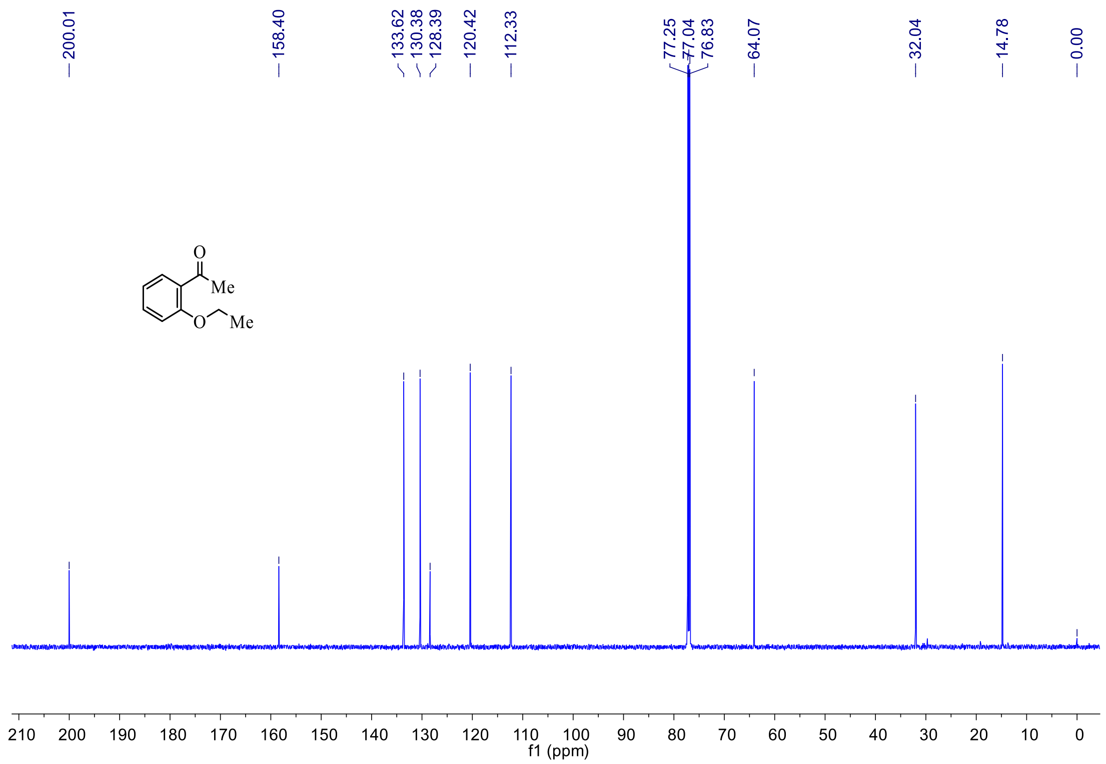

^{1}H NMR of 1-(naphthalen-1-yl)ethan-1-one **2o**

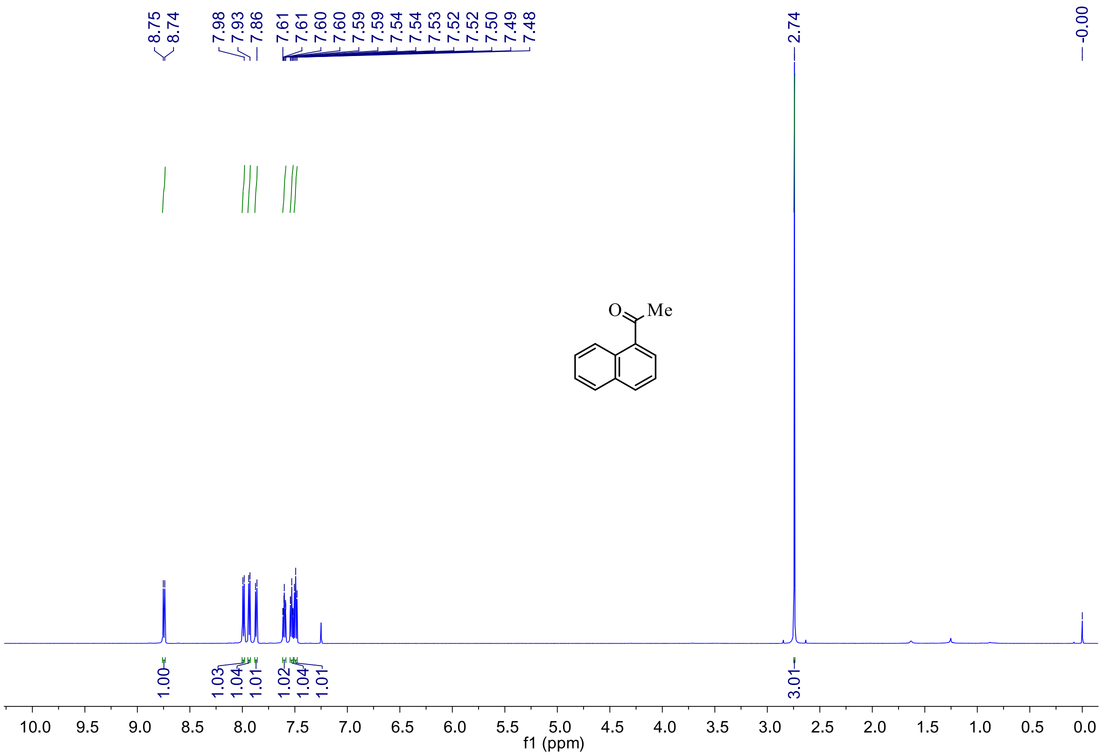

^{13}C NMR of 1-(naphthalen-1-yl)ethan-1-one **2o**

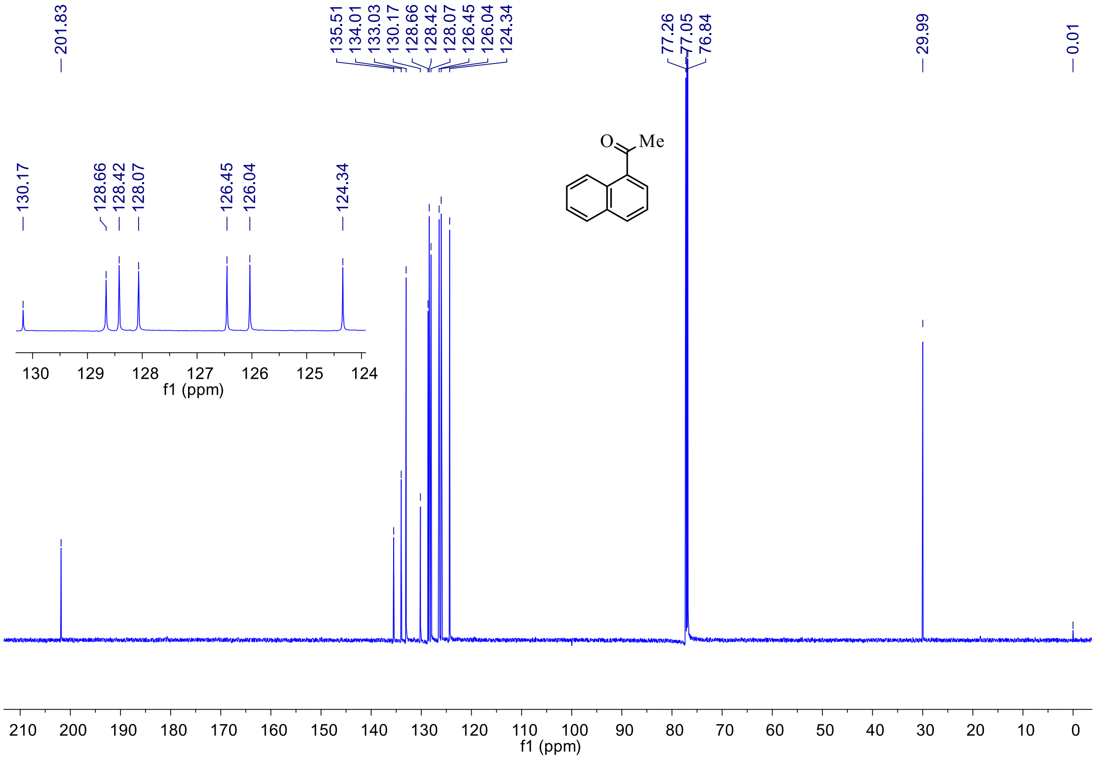

^{1}H NMR of 1-(1H-indol-4-yl)ethan-1-one **2p**

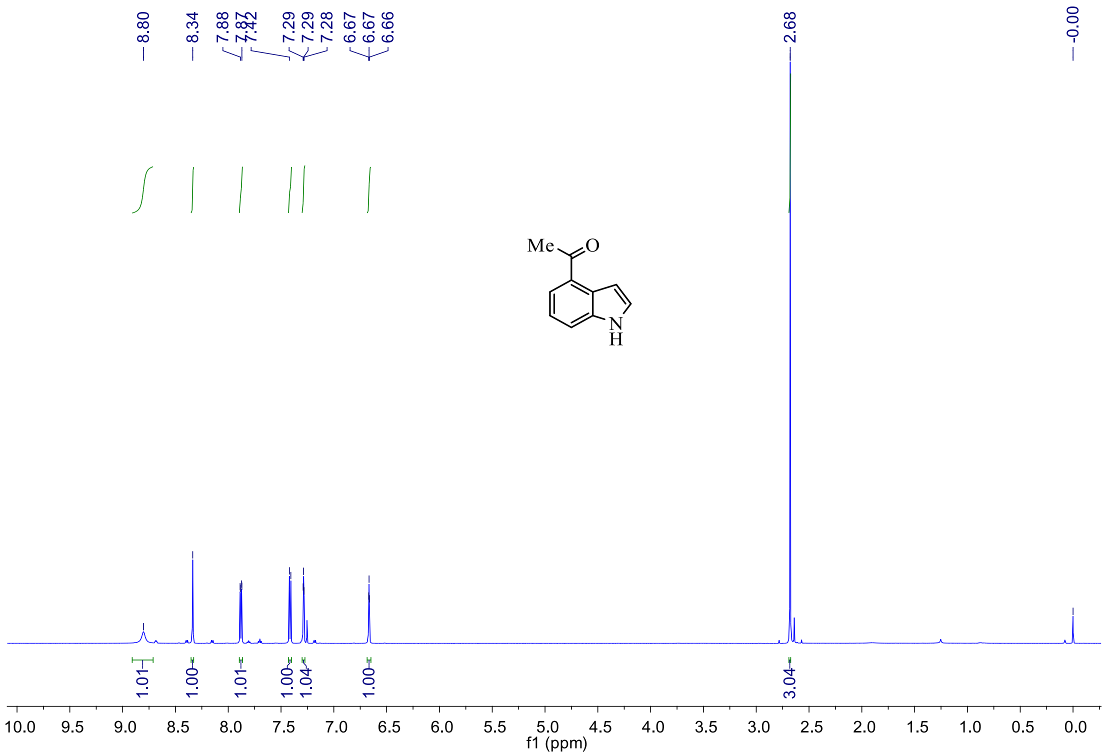

^{13}C NMR of 1-(1H-indol-4-yl)ethan-1-one **2p**

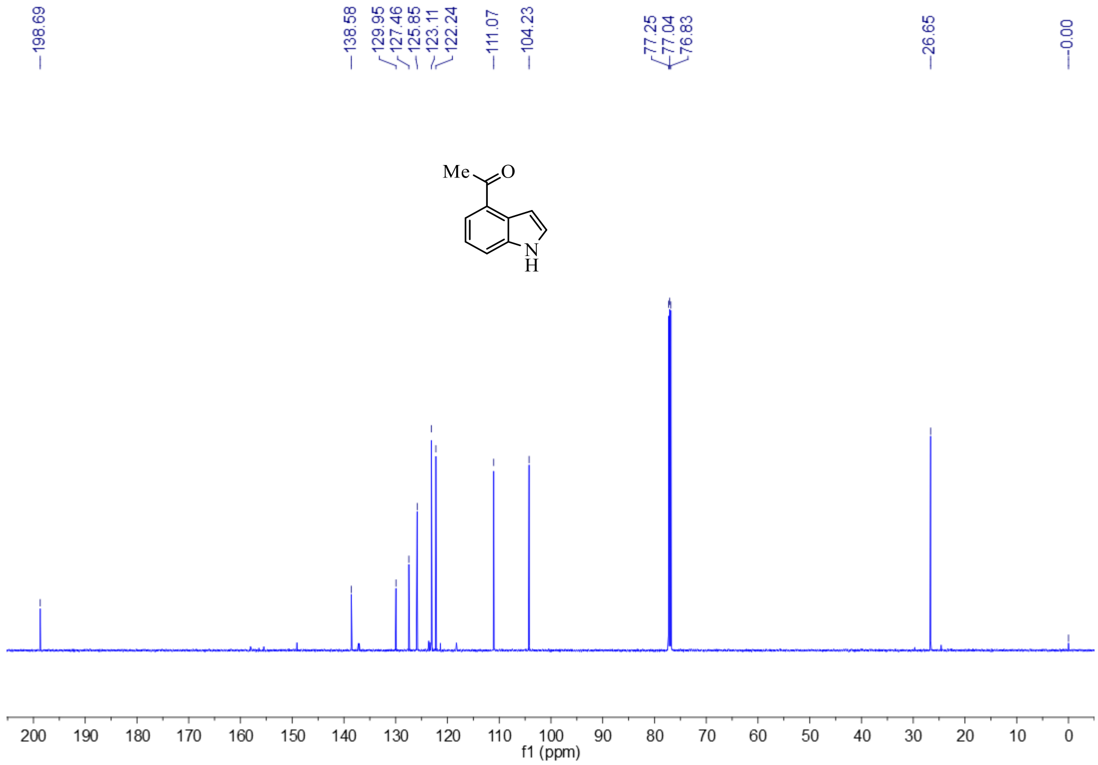

^{1}H NMR of 1-(4-(tert-butyl)phenyl)ethan-1-one **2q**

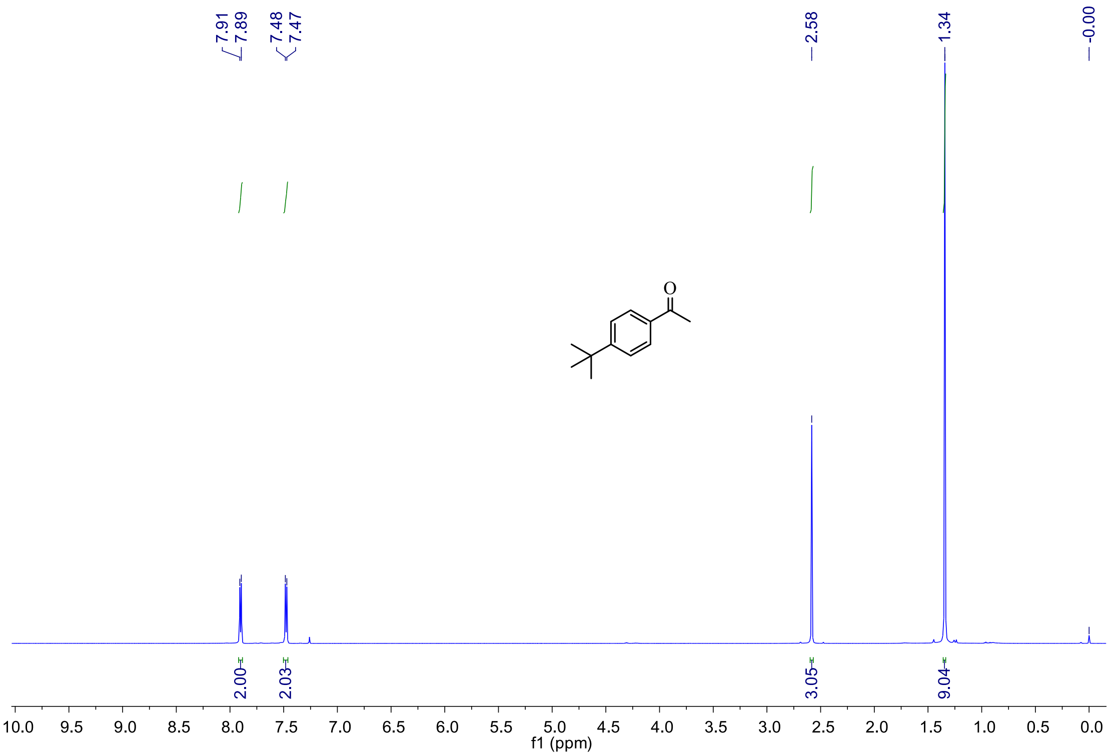

^{13}C NMR of 1-(4-(tert-butyl)phenyl)ethan-1-one **2q**

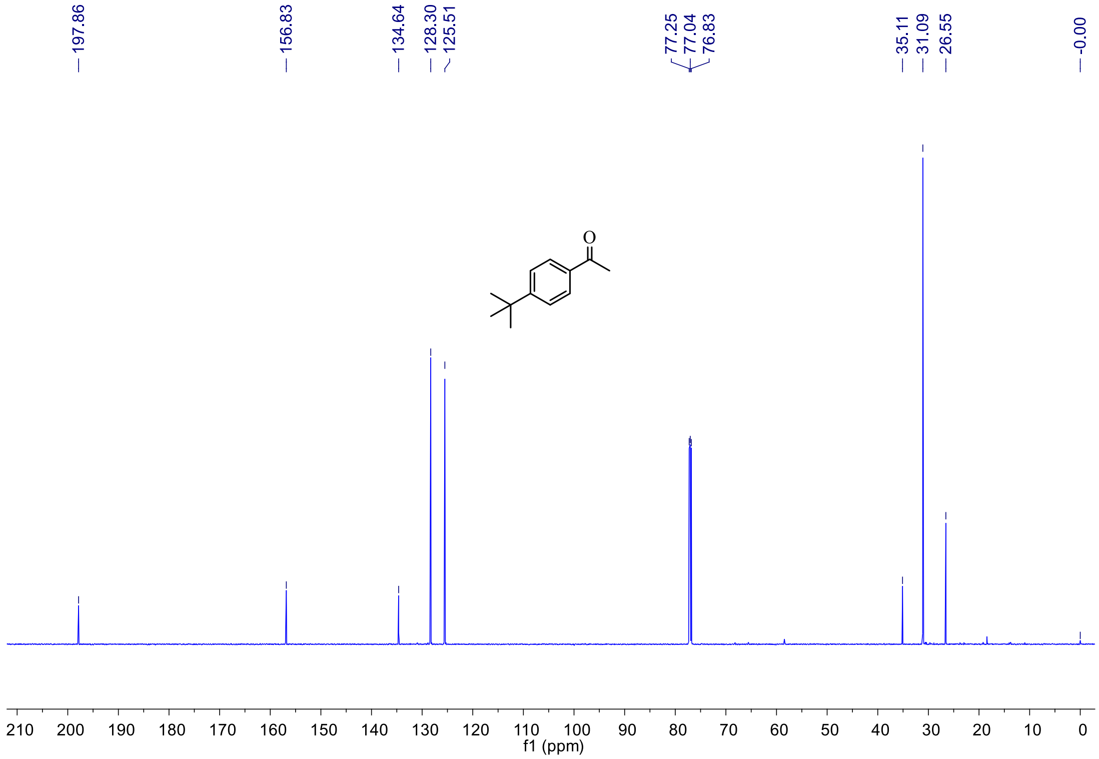

^{1}H NMR of 1-(4-methoxyphenyl)ethan-1-one **2r**

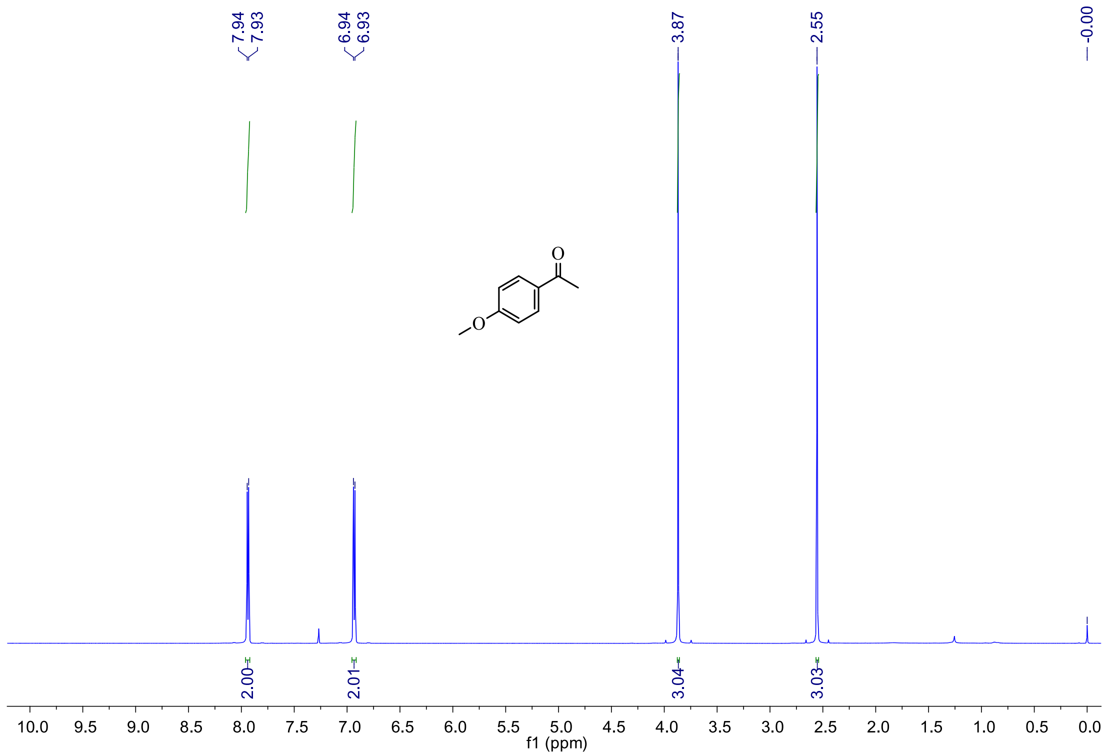

^{13}C NMR of 1-(4-methoxyphenyl)ethan-1-one **2r**

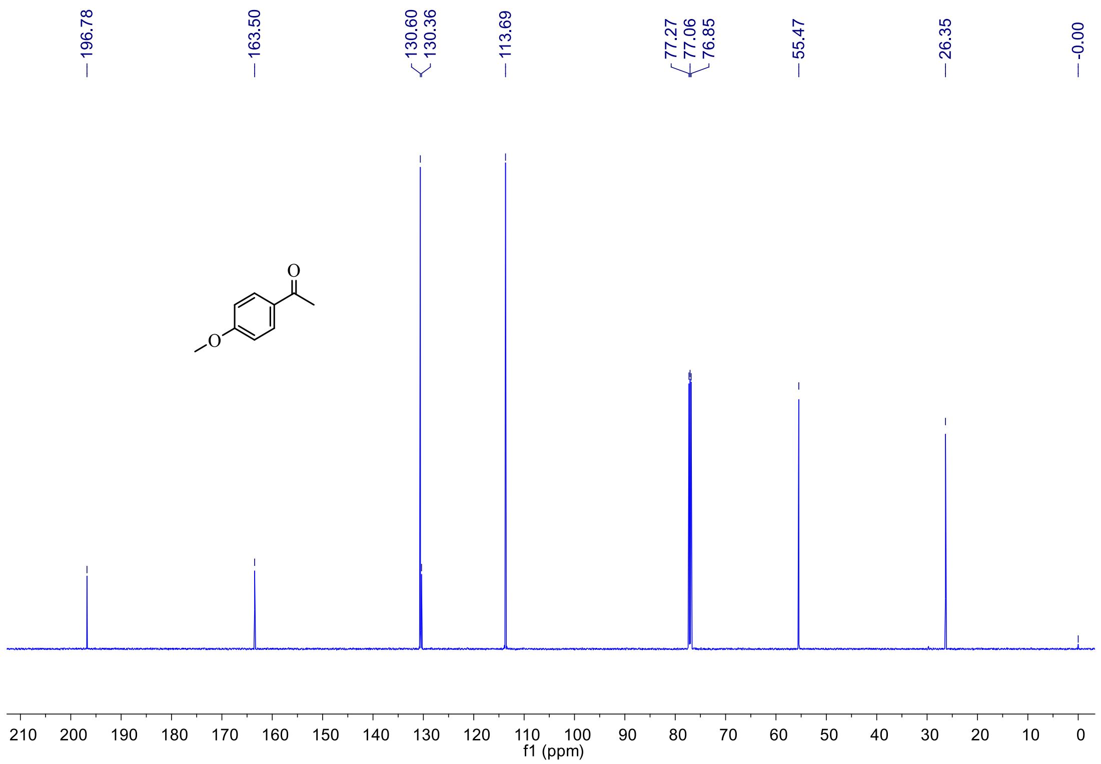

^{1}H NMR of 1-(2-methoxyphenyl)ethan-1-one **2s**

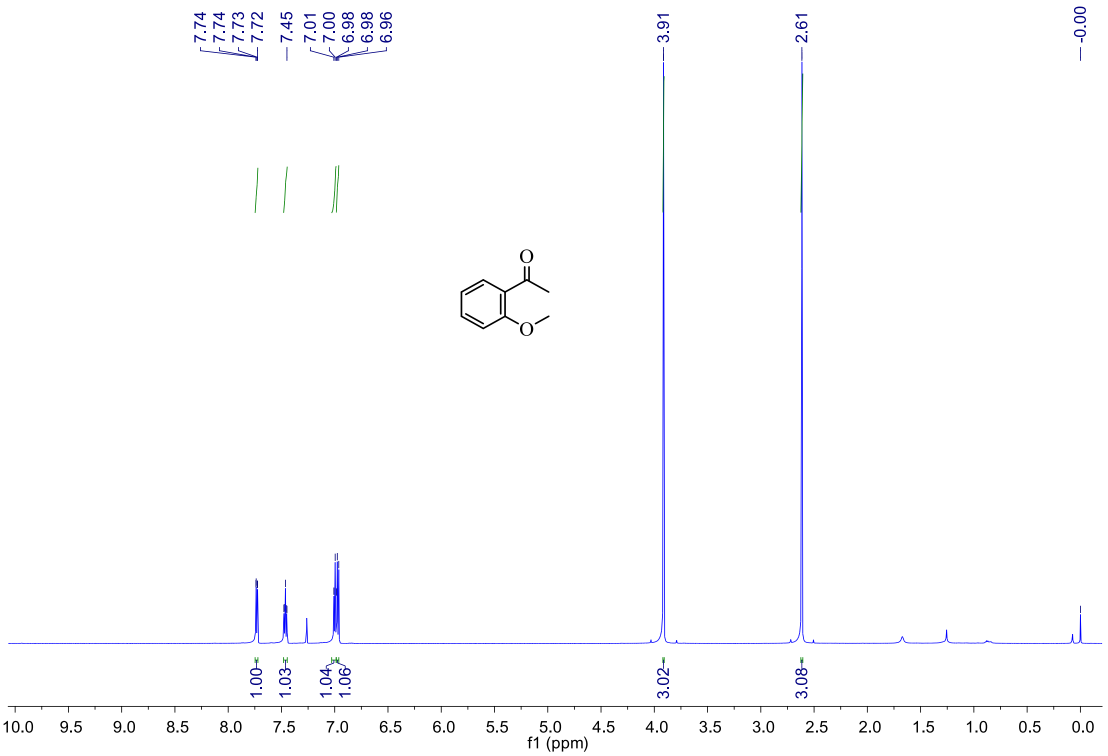

^{13}C NMR of 1-(2-methoxyphenyl)ethan-1-one **2s**

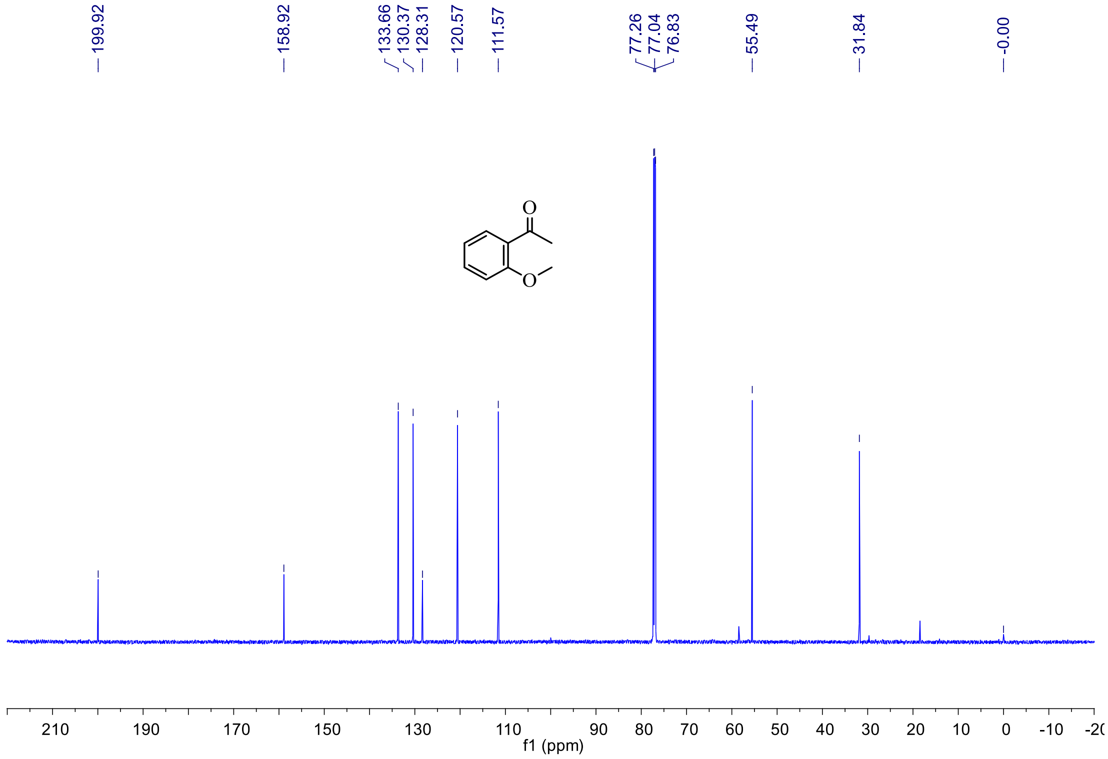

^{1}H NMR of 1-(3-benzylphenyl)ethan-1-one **2t**

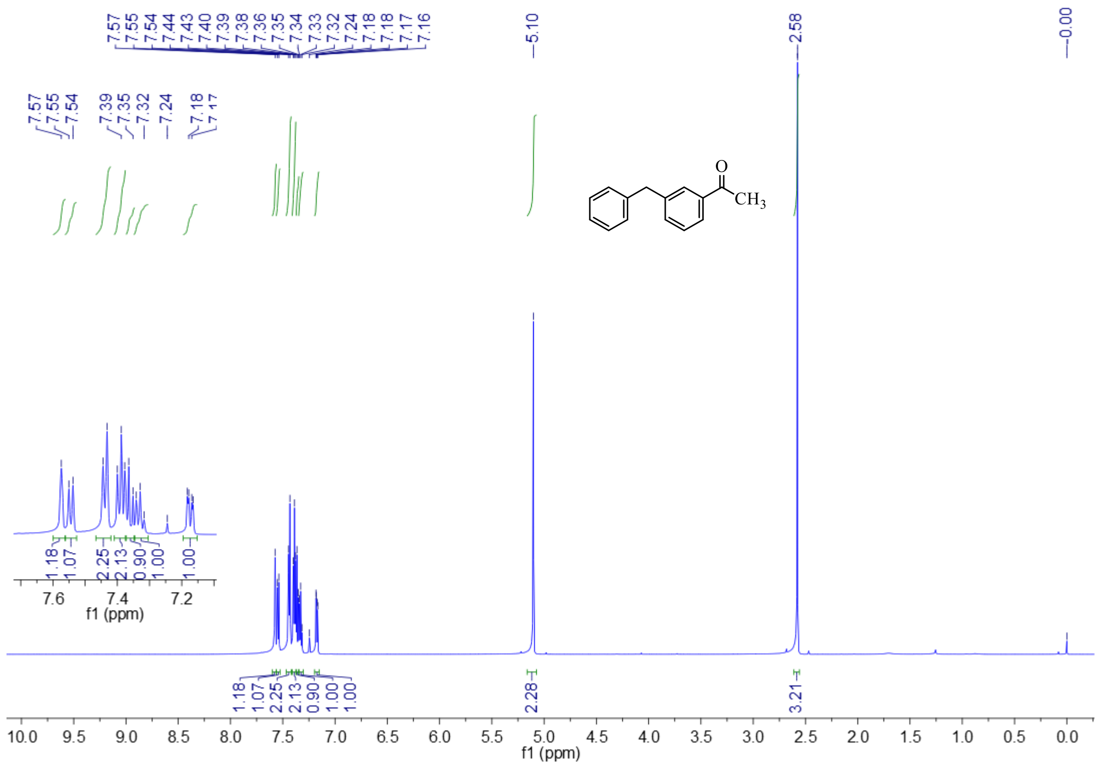

^{13}C NMR of 1-(3-benzylphenyl)ethan-1-one **2t**

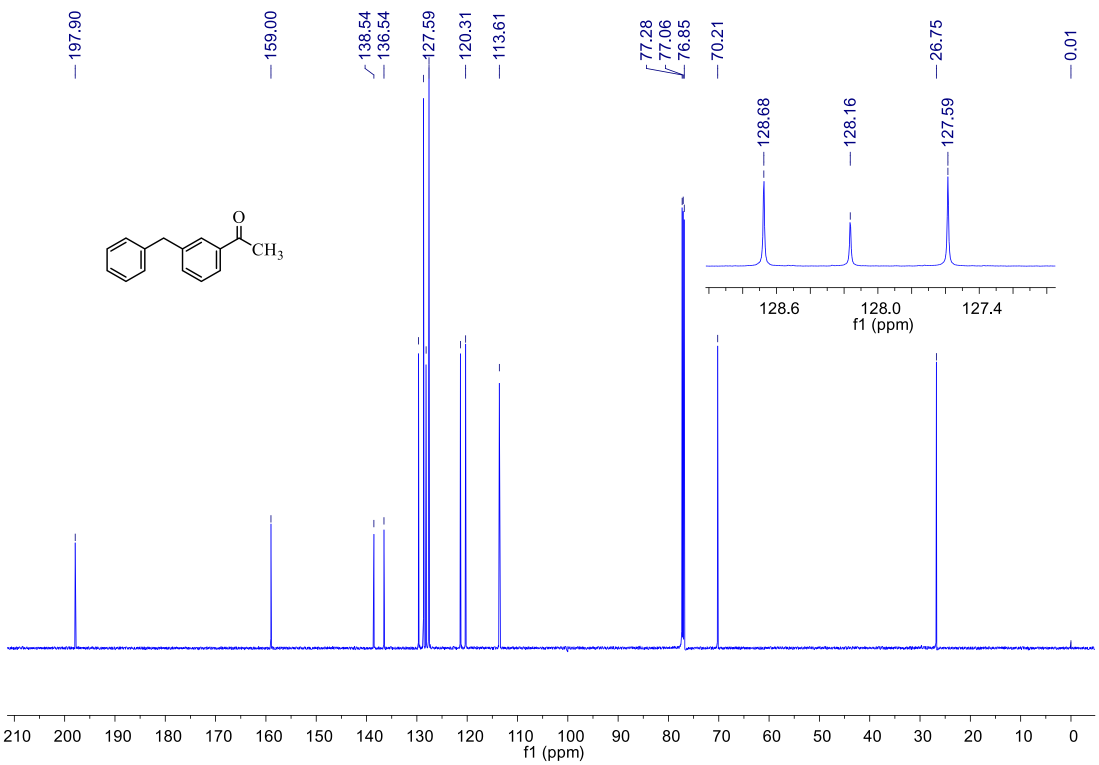

^{1}H NMR of 1-(3-methoxyphenyl)ethan-1-one **2u**

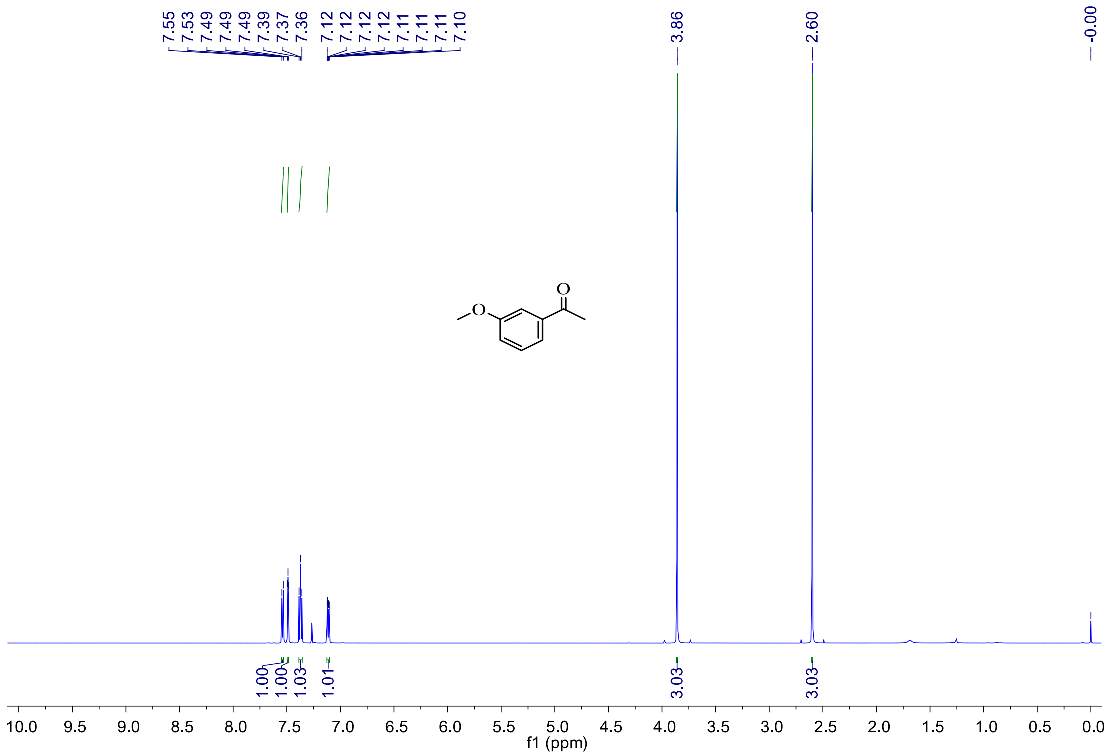

^{13}C NMR of 1-(3-methoxyphenyl)ethan-1-one **2u**

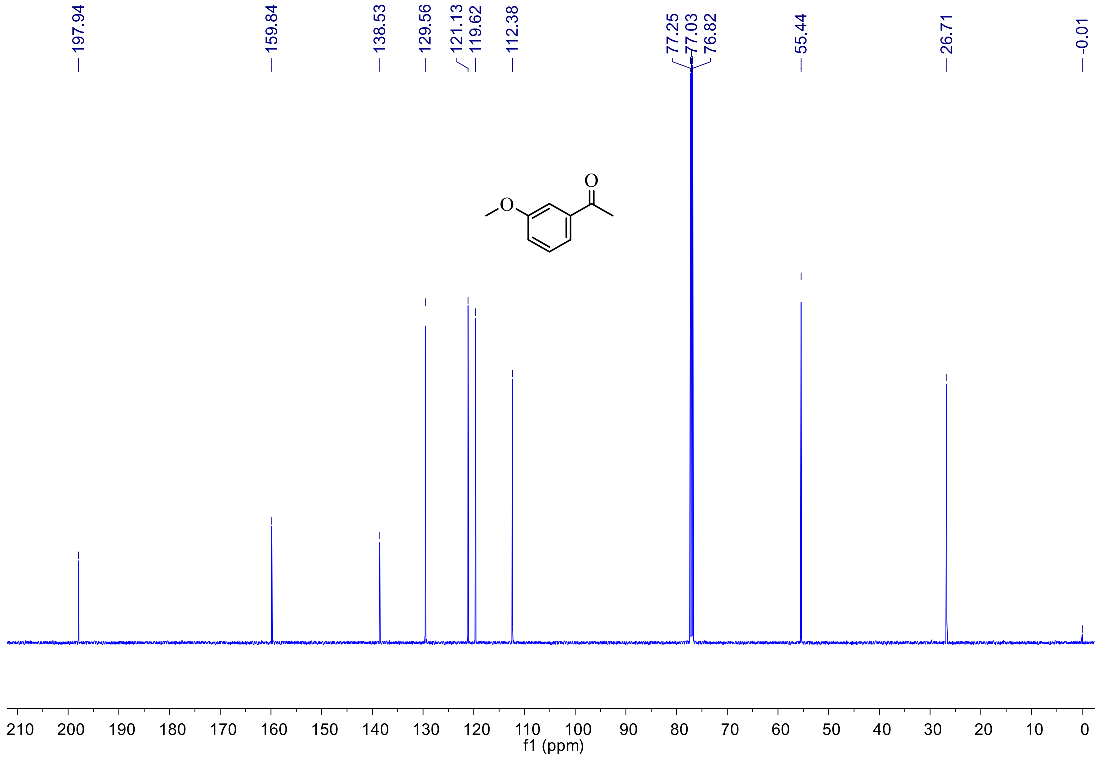

^{1}H NMR of 1-(3-hydroxyphenyl)ethan-1-one **2v**

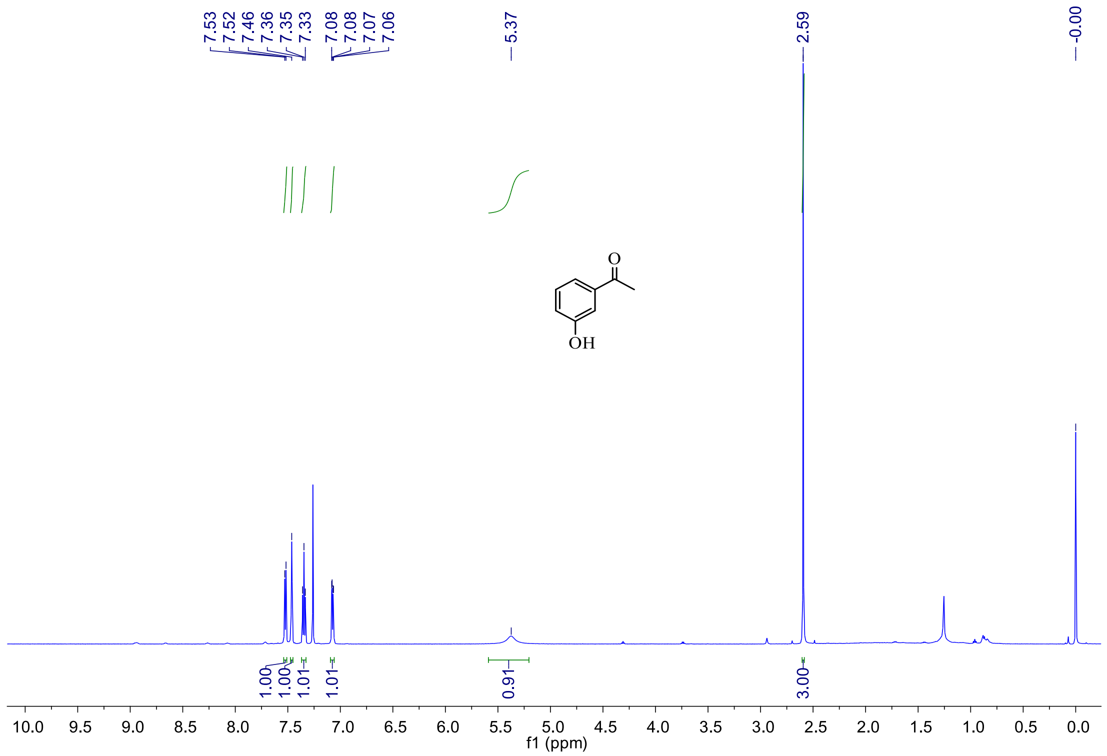

^{13}C NMR of 1-(3-hydroxyphenyl)ethan-1-one **2v**

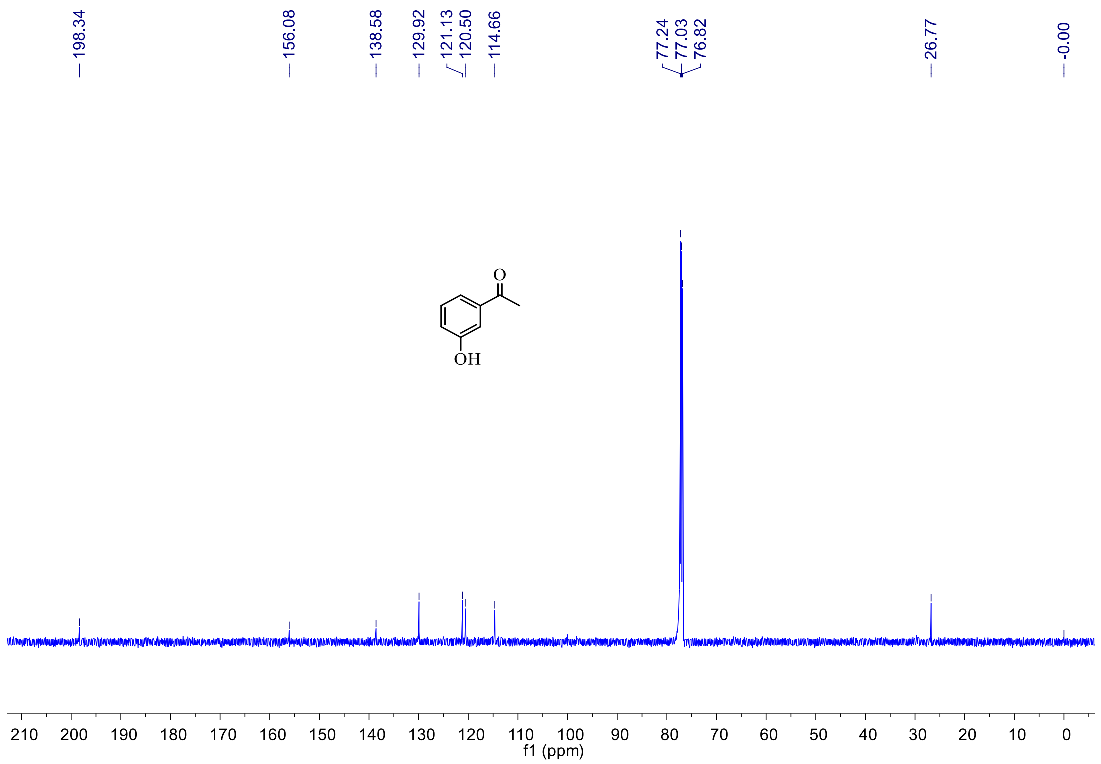

^{1}H NMR of 1-(4-fluorophenyl)ethan-1-one **2w**

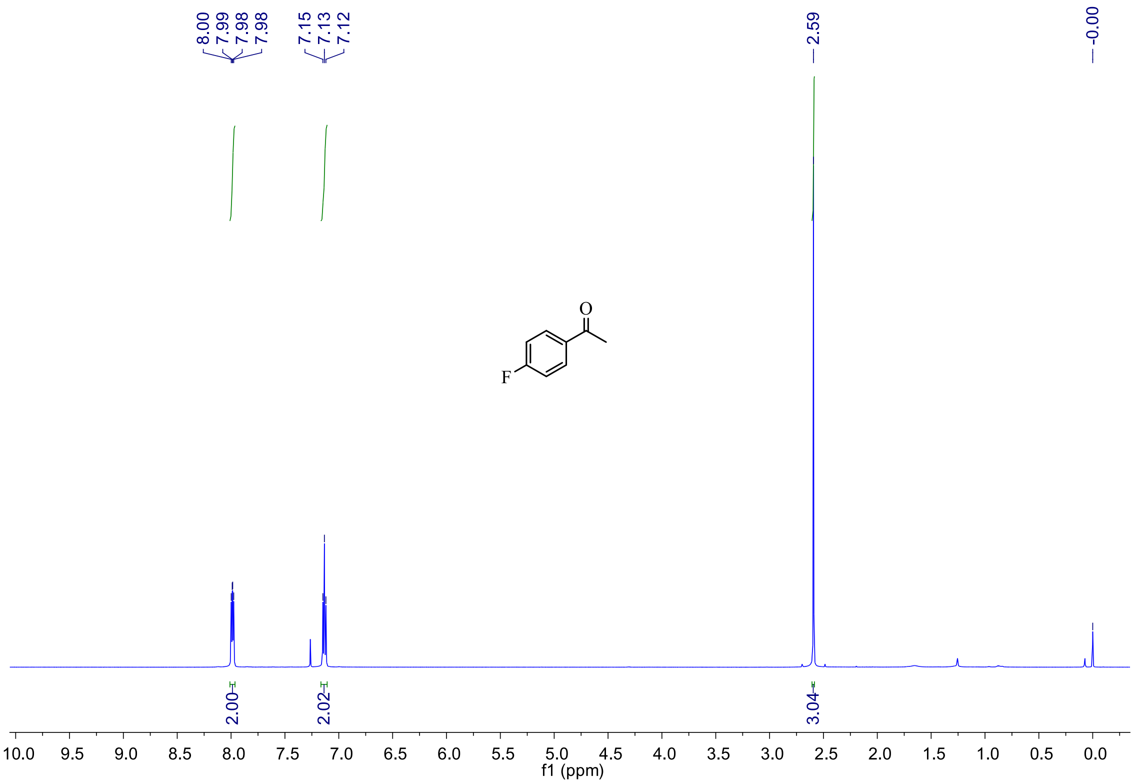

^{13}C NMR of 1-(4-fluorophenyl)ethan-1-one **2w**

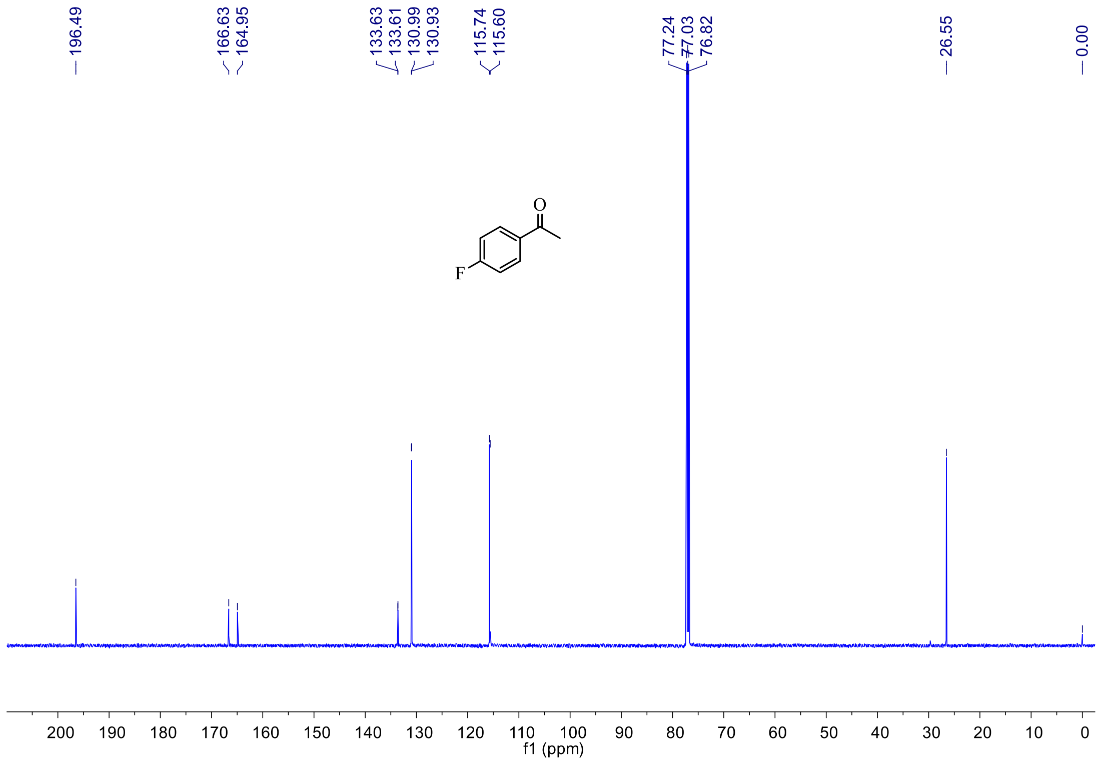

^{1}H NMR of 1-(4-chlorophenyl)ethan-1-one **2x**

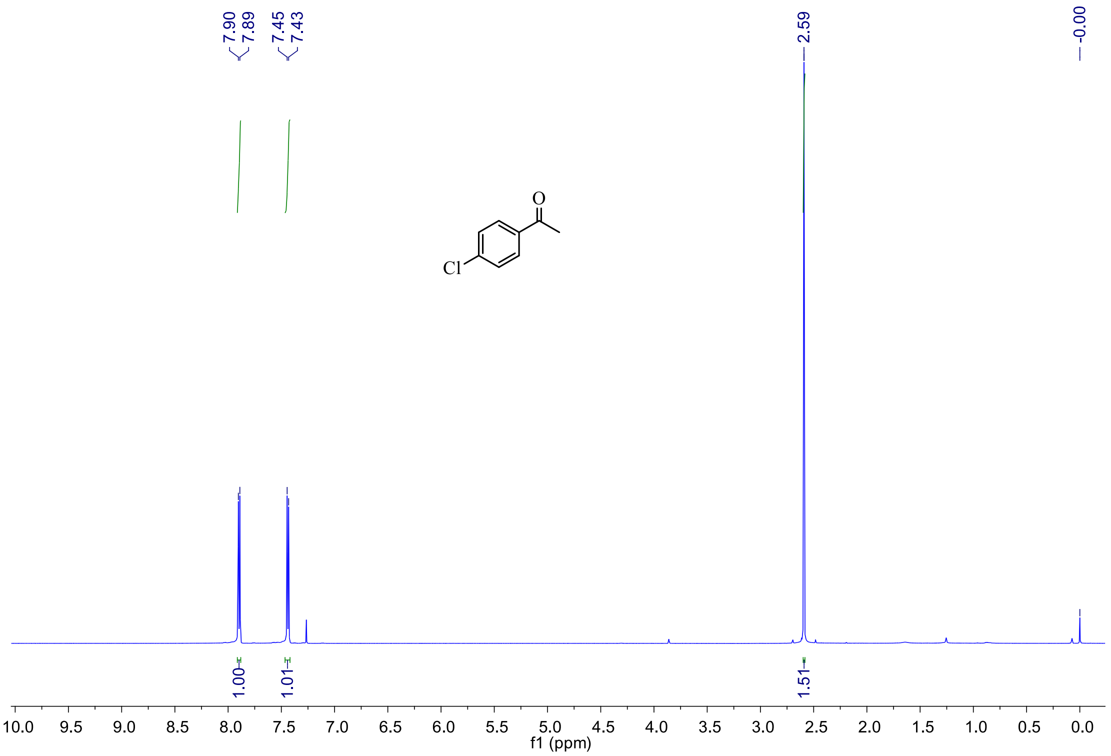

^{13}C NMR of 1-(4-chlorophenyl)ethan-1-one **2x**

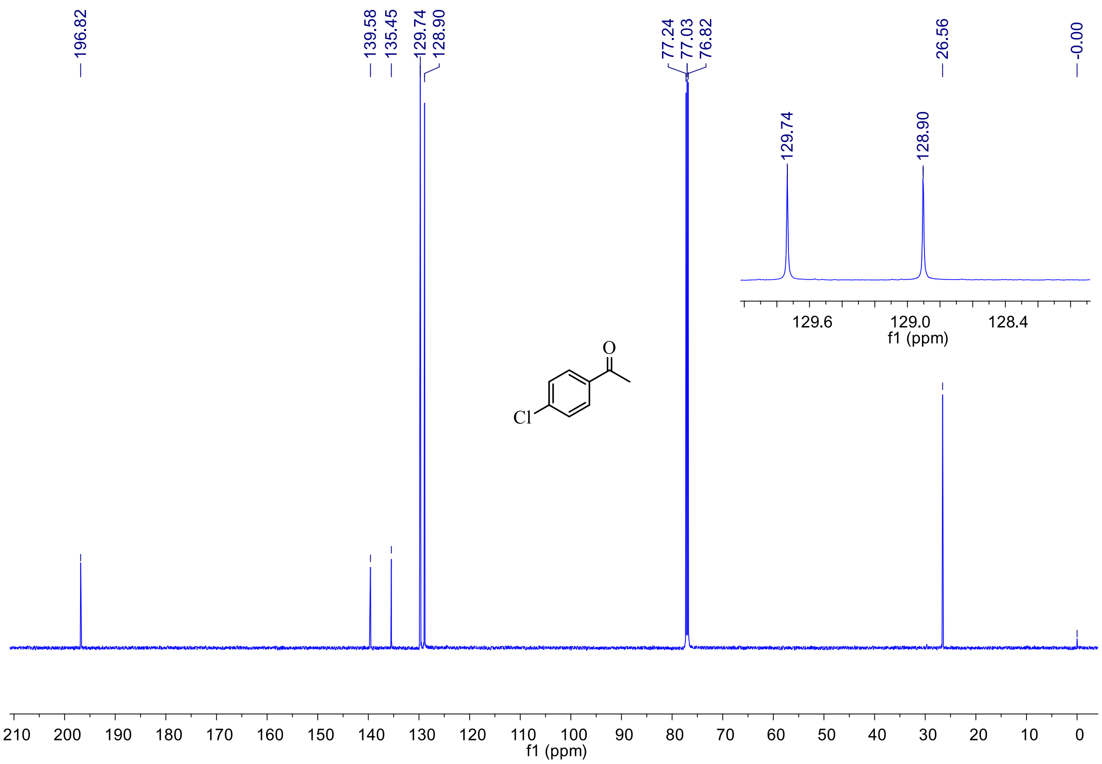

^{1}H NMR of 1-(naphthalen-2-yl)ethan-1-one **2y**

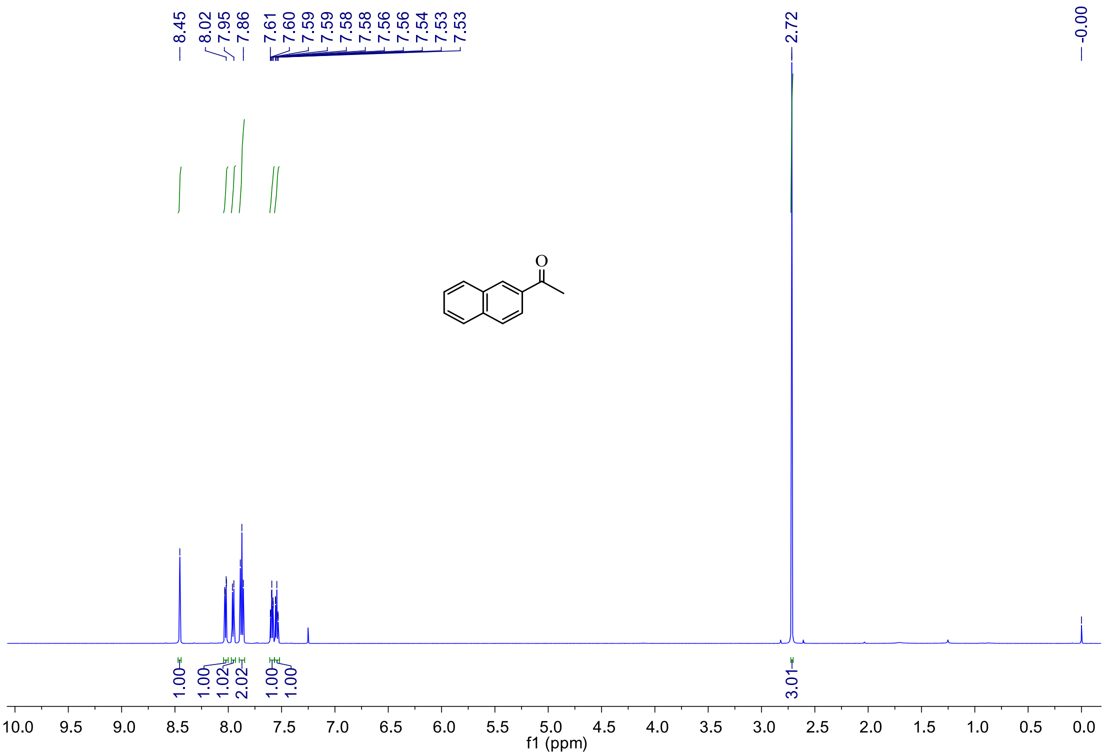

^{13}C NMR of 1-(naphthalen-2-yl)ethan-1-one **2y**

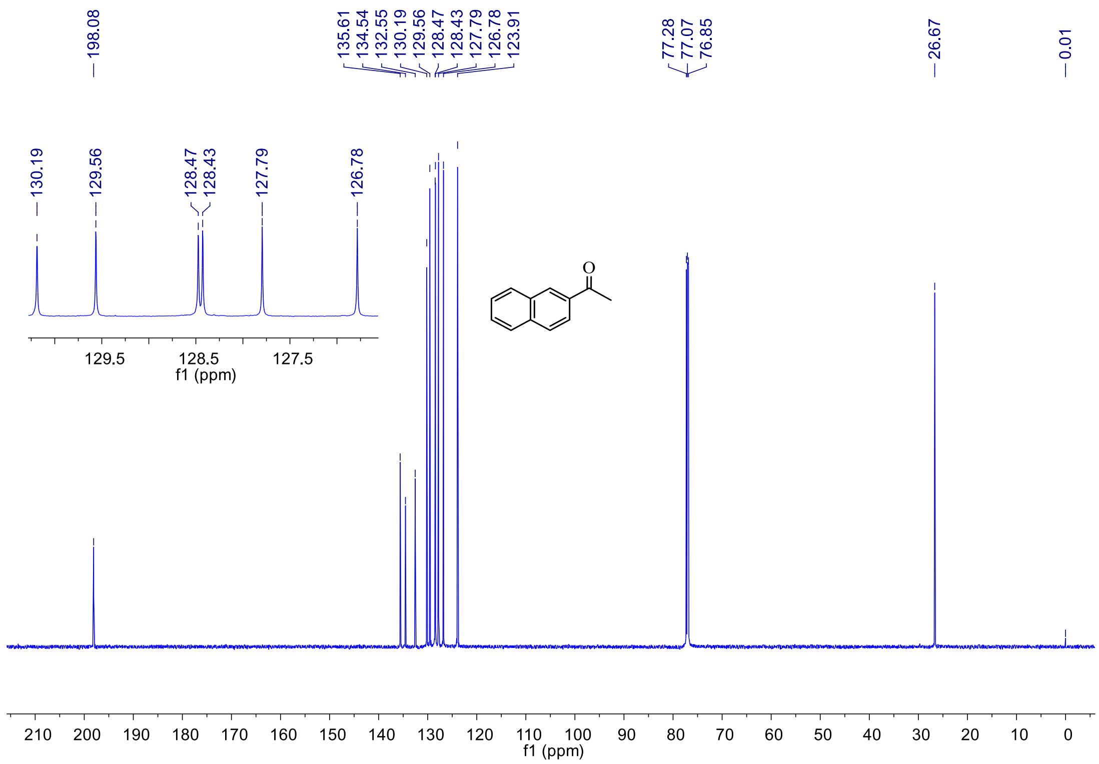

^{1}H NMR of 1-(p-tolyl)pentan-1-one **2A**

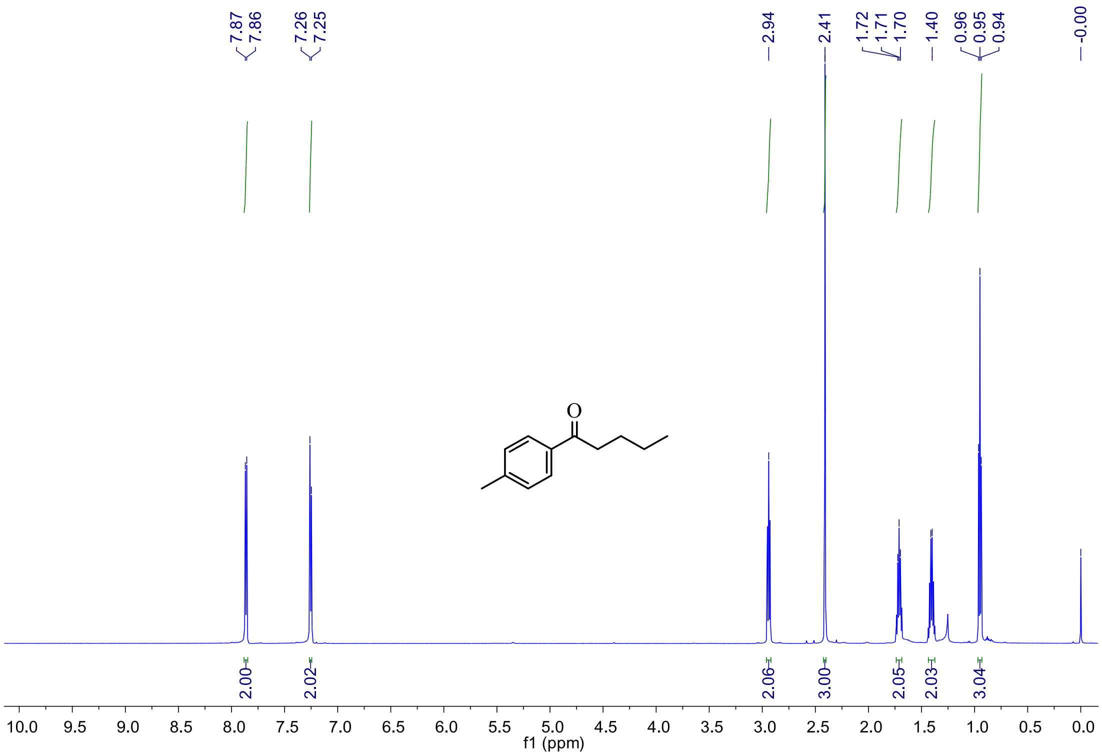

^{13}C NMR of 1-(p-tolyl)pentan-1-one **2A**

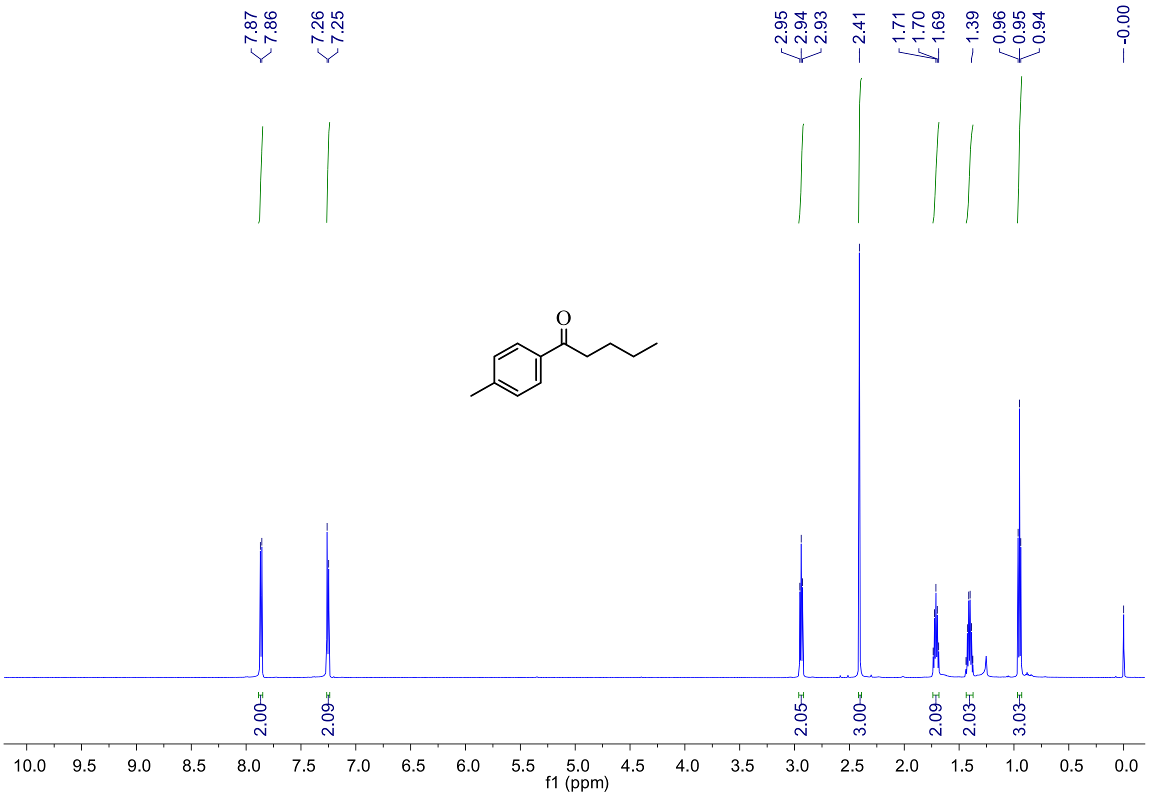

^{1}H NMR of 1-(2,6-dimethoxyphenyl)ethan-1-one **2B**

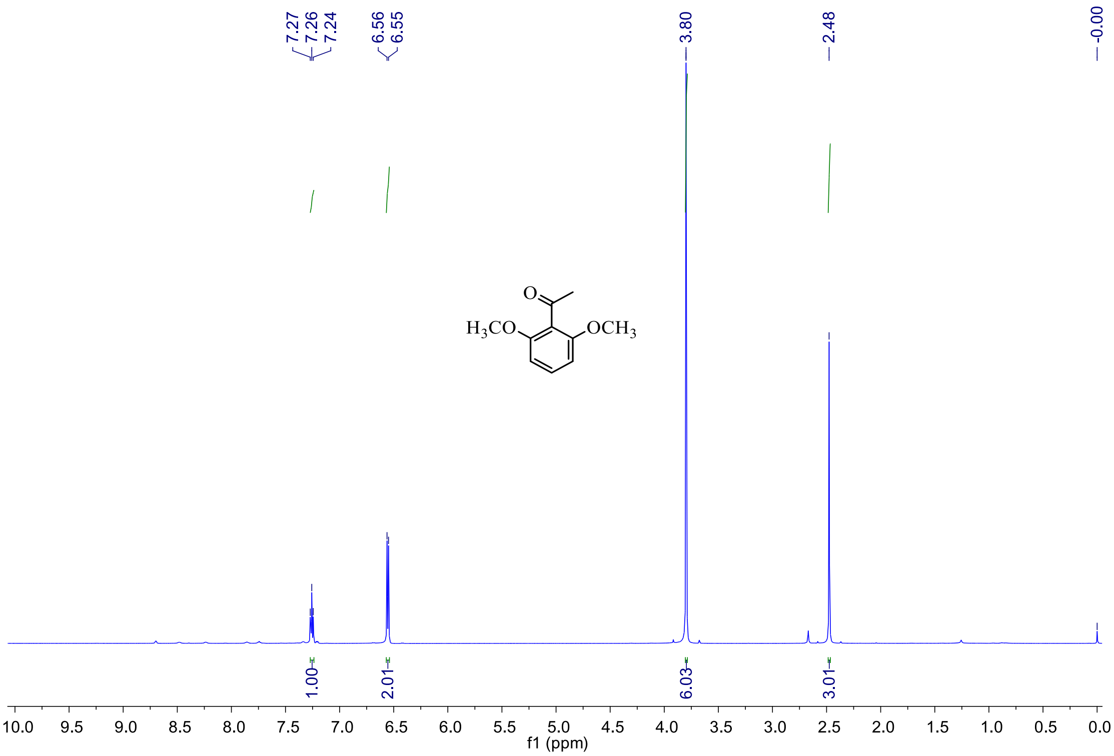

^{13}C NMR of 1-(2,6-dimethoxyphenyl)ethan-1-one **2B**

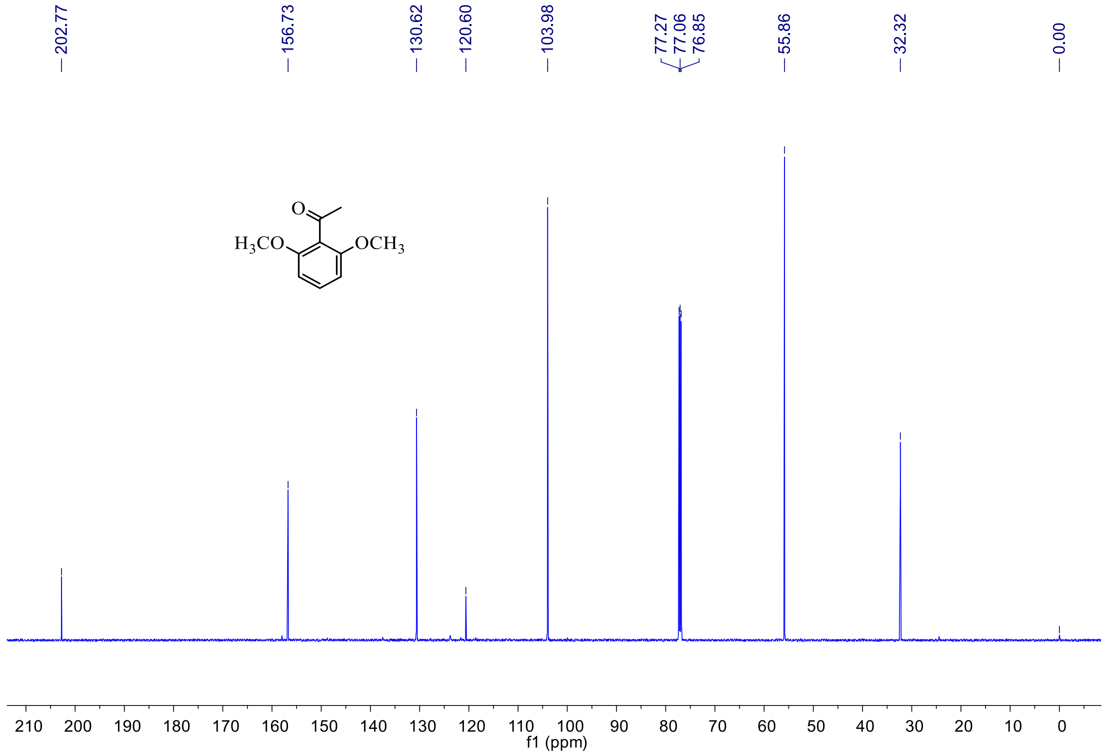

Supplement: Supplementary file 1 [file DataSheet1.PDF]
